# Molecule database framework: a framework for creating database applications with chemical structure search capability

**DOI:** 10.1186/1758-2946-5-48

**Published:** 2013-12-11

**Authors:** Joos Kiener

**Affiliations:** 1 Givaudan Schweiz AG, Fragrances S&T, Überlandstrasse 138, 8600 Dübendorf, Switzerland

**Keywords:** Chemical structure search, Database, Framework, Open-source

## Abstract

**Background:**

Research in organic chemistry generates samples of novel chemicals together with their properties and other related data. The involved scientists must be able to store this data and search it by chemical structure. There are commercial solutions for common needs like chemical registration systems or electronic lab notebooks. However for specific requirements of in-house databases and processes no such solutions exist. Another issue is that commercial solutions have the risk of vendor lock-in and may require an expensive license of a proprietary relational database management system. To speed up and simplify the development for applications that require chemical structure search capabilities, I have developed Molecule Database Framework. The framework abstracts the storing and searching of chemical structures into method calls. Therefore software developers do not require extensive knowledge about chemistry and the underlying database cartridge. This decreases application development time.

**Results:**

Molecule Database Framework is written in Java and I created it by integrating existing free and open-source tools and frameworks. The core functionality includes:

• Support for multi-component compounds (mixtures)

• Import and export of SD-files

• Optional security (authorization)

For chemical structure searching Molecule Database Framework leverages the capabilities of the Bingo Cartridge for PostgreSQL and provides type-safe searching, caching, transactions and optional method level security. Molecule Database Framework supports multi-component chemical compounds (mixtures).

Furthermore the design of entity classes and the reasoning behind it are explained. By means of a simple web application I describe how the framework could be used. I then benchmarked this example application to create some basic performance expectations for chemical structure searches and import and export of SD-files.

**Conclusions:**

By using a simple web application it was shown that Molecule Database Framework successfully abstracts chemical structure searches and SD-File import and export to simple method calls. The framework offers good search performance on a standard laptop without any database tuning. This is also due to the fact that chemical structure searches are paged and cached. Molecule Database Framework is available for download on the projects web page on bitbucket: https://bitbucket.org/kienerj/moleculedatabaseframework.

## Background

In organic chemistry research the chemists synthesize novel compounds that exhibit the desired properties and perform better than existing compounds. The commercial objective is to sell these new and superior compounds directly or in new products containing them. During the process from the initial creation of a new compound until it is commercialized, a lot of data related to it is generated such as physiochemical properties, analytical- and toxicological data. All this data needs to be stored and scientists must be able to search it by chemical structure.

The chemist performs a chemical reaction and enters the relevant chemical structures, the exact procedure and outcome into his/her electronic lab notebook (ELN) so that other chemists can find it and repeat the reaction if necessary. After this new compound is synthesized, a new entry for it is generated in the chemical registration system. Unfortunately there is not much scientific literature concerning how and what such registration systems do [1]. The main purpose is to assign each compound a unique in-house reference number that then can be used to cross-reference it. There are many commercial software solutions for ELNs and chemical registrations such as those from PerkinElmer (CambridgeSoft), Accelrys, ChemAxon or Dotmatics to name a few. However these solutions may be costly especially if they also require a commercial relational database management system (RDBMS). An exception is ChemAxon Compound Registration [2] which runs on MySQL. Another issue is that they are closed-source and if such systems are used in the context of scientific experiments, the experiment is not fully reproducible by other scientists unless they also have access to a license for the same system. Because these systems need to be highly configurable, they are also very complex. So configuring and administrating such a system requires a lot of very specific expertise. Consequentially it can be advantageous to build your own solution especially for organisations that already have an in-house software development department. A custom solution does not have to be highly configurable as it can be tailored to your needs. This includes administrative interfaces for user management and specific tasks like informing all users about new features. Such tools can greatly reduce administrative overhead.

Another common requirement is in-house compound databases and processes for which no commercial solutions are available. These databases might not store information on novel compounds but are still important in the discovery process. For example, an organisation wishes to gather and store information on commercially available products from competitors or other suppliers such as purity, pricing and other properties. With PerkinElmer’s BioSAR application you can search for a chemical structure in any database table in oracle that was indexed by their cartridge. However it offers no easy solution for data entry. Either a trained expert needs to enter the data or a custom web interface for easy data entry must be developed. Dotmatics Browser application is very similar to BioSAR in this regard. In general there seems to be a need to create custom chemical structure searchable database applications [3,4].

As a solution for these issues Molecule Database Framework (MDF) was created. MDF can be used to create chemical structure search enabled database applications. One of the advantages compared to directly using a chemical database cartridge is, that all code to connect, access and query the database is provided by MDF. It abstracts the storing and querying of chemical structures into method calls. The software developers using MDF do not require extensive knowledge of either chemistry or the underlying database cartridge.

MDF has been designed with a flexible domain model. An application can have many types of chemical compounds like a registration compound and an inventory compound which can have different properties. Samples of these different types of chemical compounds can be associated with a container identified by a unique barcode. Such containers can then be searched by chemical structure.

The design and functionality of MDF makes it possible to build many different types of systems like registration systems, inventory systems and simple compound databases or all of these together in one single integrated solution.

## Implementation

### Open-Source software components

Molecule Database Framework was developed by integrating free, open-source software components (Table 1). These components are backed by a commercial organisation offering services or are wide-spread and have a large base of contributors like PostgreSQL. This means all these components are well maintained and should be future proof.

**Table 1:** Main software components

**Component**	**Supplier**
PostgreSQL	http://www.postgresql.org/
Bingo PostgreSQL Cartridge	GGA Software Services
Indigo Toolkit	GGA Software Services
Spring	Pivotal (owned by VMware and EMC)
Hibernate	JBoss (Red Hat)
QueryDSL	Mysema

PostgreSQL is the RDBMS used for MDF. It is Open-Source and available for different platforms (Linux, BSD, Solaris, Windows, OS X).

Bingo PostgreSQL Cartridge is an extension to PostgreSQL that enables chemical structure search within PostgreSQL. Bingo offers functionality that not all commercial cartridges have like advanced tautomer search and resonance substructure search [5]. It is also available for Oracle and SQL Server. Bingo is responsible for the chemical structure searches in MDF.

The Indigo Toolkit is a chemistry toolkit written in C++ with application programming interfaces (API) for various other languages like Python, Java or .Net. Indigo is used for format conversion, import and export of SD-Files and for generating chemical structure depictions including highlighted substructures.

The Spring Framework is a framework for creating a diverse range of Java applications. It can be seen as an alternative to Java EE. Its core module is an inversion of control container for injecting dependencies at runtime. For example this means that the behaviour of an application can be changed by using a different implementation of an interface. Which implementation to use can be configured in an Extensible Markup Language (XML) file and does not require any code changes. To activate such a configuration change, the application must be restarted.

MDF also uses the Spring Data JPA and Spring Security modules. Spring Data JPA is for fast creation of Java Persistence API (JPA) based data access layers. Spring Security is used for method level security and security-aware database queries (results are limited to content that a user is allowed to see on the database level).

### Software layers

**Figure 1:**
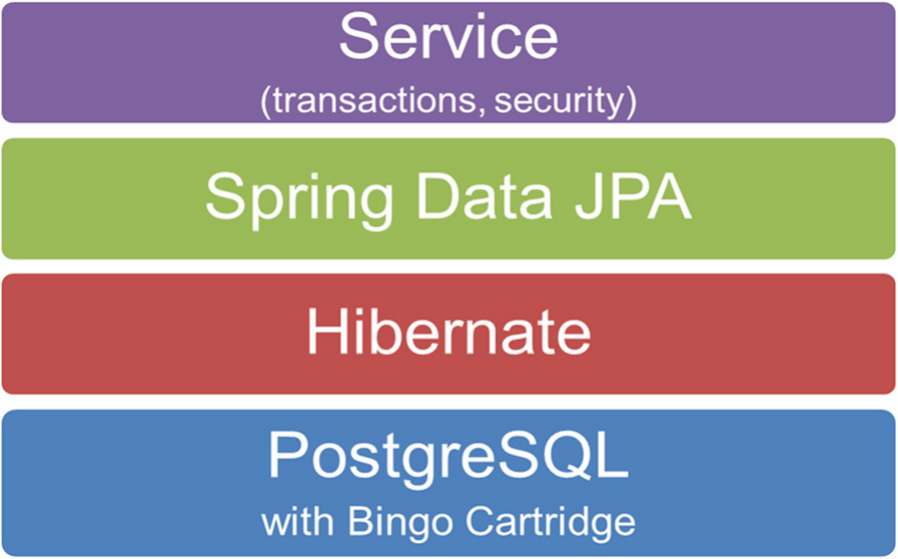
**A schematic representation of the 4 software layers in MDF.** The 4 layers are: the database using PostgreSQL (blue), Object Relational Mapping (ORM) provided by Hibernate using JPA (red), the data access layer implemented with Spring Data JPA (green) and a service layer for transaction support and security (purple).

MDF consists of the 4 layers: the database using PostgreSQL, Object Relational Mapping (ORM) provided by Hibernate using JPA, the data access layer implemented with Spring Data JPA and a service layer for transaction support and security (Figure 1).

### Entity classes

**Figure 2:**
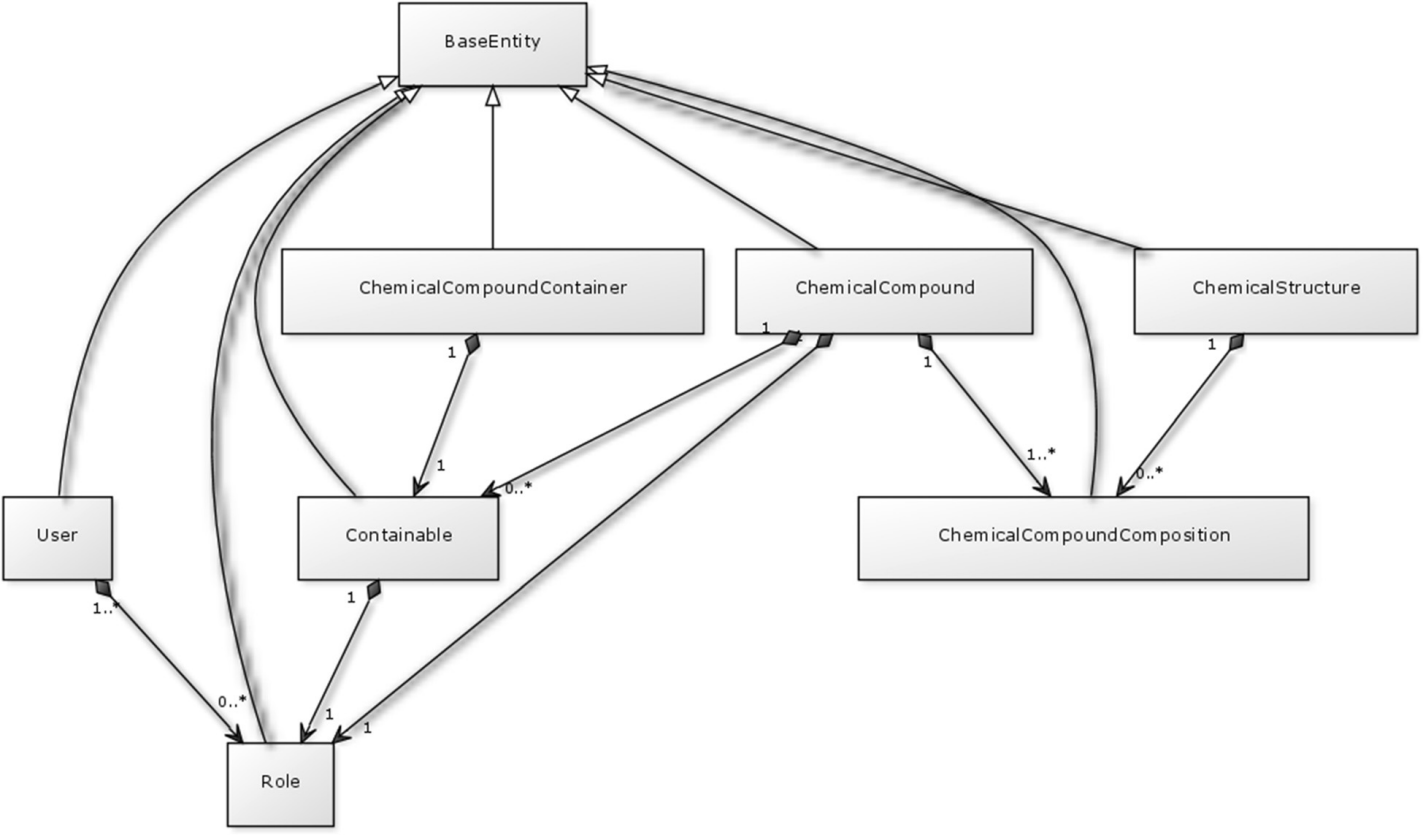
**UML class diagram.** The image shows the UML class diagram of the entity classes of MDF. Fields and methods are omitted. The focus is on the relationships between the entity classes.

MDF uses JPA with Hibernate as its implementation. All entity classes are therefore annotated with JPA annotations. The entity classes are shown as an UML Class diagram in Figure 2.

### Base entity

BaseEntity is an abstract class that manages metadata like creation and last modified date and who created and modified the entity. It contains the abstract getId() method to retrieve the unique database id. BaseEntity is further responsible for the optimistic locking feature of JPA. It contains a column annotated with @Version. If an entity is updated, the numeric version column is incremented by 1. Update statements always contain the version in the where-clause and if a concurrent update occurs, they do not match and the concurrent modification is detected.

### Chemical structure

ChemicalStructure entity holds the chemical structure data (SMILES [6] or molfile) and the structure key (InChikey). A ChemicalStructure is unique and immutable and managed by MDF. Users operate on ChemicalCompounds and not on ChemicalStructures directly. Unique means that when a new ChemicalCompound is saved, the framework checks if the referenced ChemicalStructures already exist and if yes, reuses them. The existence check is done by using the standard InChikey which has a unique constraint in the database. Immutable means that if a ChemicalCompound is updated and one of the ChemicalStructures was changed, MDF will automatically check if the updated ChemicalStructure already exists and will use it or create a new ChemicalStructure. The old one will remain unchanged.

While the Bingo cartridge offers a molecular weight search without explicitly storing it in the database, this cannot yet be combined with a chemical structure search. Therefore MDF automatically calculates the molecular weight of each ChemicalStructure and saves it to the database. This allows structure searches within a specified molecular weight range.

### Chemical compound composition

ChemicalCompoundComposition links together ChemicalStructure and ChemicalCompound and defines the relative occurrence of the ChemicalStructure within the ChemicalCompound.

### Chemical compound

The ChemicalCompound entity is an abstract model of a chemical compound. A ChemicalCompound is a “descriptive entity”. It is not a concrete object that physically exists. It consists of ChemicalCompoundCompositions. A ChemicalCompound is therefore a mixture if it consists of multiple ChemicalCompoundCompositions (see Figure 2).

The class contains some basic fields like compoundName and cas. ChemicalCompounds can also be associated with Containables. Developers using MDF must create concrete implementations of this abstract class. An application can have multiple implementations of ChemicalCompound and each implementation is stored and searched separately (Table per Concrete class Inheritance). Note that due to better usability it was decided to make CAS-Number column nullable and it is not unique. This behavior can be changed by manually creating the according constraints in the database.

### Containable

A Containable is a set of a ChemicalCompounds that have a common source. For example they were produced in the same way. In a Chemical Registration System this would be a Batch and in an Inventory System a Lot. The important part is that ChemicalCompound and Containable are generic classes and must form a pair:

### Chemical compound container

A ChemicalCompoundContainer holds exactly one Containable of any type. An application should only have one implementation of ChemicalCompoundContainer. A ChemicalCompoundContainer instance represents a concrete physically available object containing a ChemicalCompound linked by a Containable. ChemicalCompoundContainer has a barcode field which is unique and not nullable. The barcode hence uniquely identifies a physically available sample of a ChemicalCompound.

### Role and user

Role and User are only relevant if MDF is used with Spring Security. ChemicalCompound and Containable hold a reference to their read-role. This is used to filter ChemicalCompoundContainers in the database based on the current users’ privileges. Example:

An application has two ChemicalCompound-Implementations, DefaultCompound and SecretCompound. Current user has the privilege to read DefaultCompounds but not for reading (viewing) SecretCompounds. So if this user searches for ChemicalCompoundContainers, only ChemicalCompoundContainers that contain a DefaultCompound must be returned by the search. To achieve that, the query’s WHERE-clause is extended and the filter based on the Role is added automatically. The main advantage of doing this filtering in the database compared to filtering the results within the applications is that you get pageable results which would not be easily possible (if at all) with application-side filtering.

### Data access layer

#### *Spring data JPA*

The data access layer was created with Spring Data JPA. Spring Data JPA is a module for fast creation of data access layers. It provides methods for create, read, update and delete (CRUD) operations. The developer does not need to write any code for this.

When using Spring Data you create a new interface that extends from generic interfaces provided by Spring Data JPA and represents the repository for an entity. There are different kinds of repository interfaces but the repositories in MDF all extend JpaRepository. JpaRepository provides CRUD-methods and basic retrieval methods for your entity. All these methods are implemented automatically by Spring Data. The interface itself can remain completely empty.

Repositories in MDF also extend QueryDslPredicateExecutor. This adds findOne(predicate) and findAll(predicate) methods. Predicates are basically type-safe WHERE-Clauses provided by QueryDSL.

Repositories can also contain custom find-methods. The convention is that the methods starts with “findBy” and is then followed by a path. An example would be the method T findByCas(String cas) in ChemicalCompoundRepository. You only need to write the method signature into your interface. The implementation is created automatically by Spring Data.

Another option is to annotate a repository interface method with @Query. The value of the annotation is either a JPQL Query or native SQL.

### Chemical structure search

The main challenge was how to make Bingo Cartridge methods usable as Spring Data Repository methods. This involved two steps: First the Bingo Cartridge methods were exposed to Hibernate by extending PostgreSQL82Dialect and then were used by creating custom Repository implementations.

Spring Data JPA allows you to create method implementations and to use them in your repository interface. In MDF a generic interface for chemical structure search was created.

A repository offering chemical structure search extends above interface.

As a last step the methods from ChemicalStructureSearchRepository must be implemented. This can be done in a custom repository implementation. The convention is to name the implementation the same as the interface and add “Impl” at the end of the name.

Spring Data JPA will merge the interface ChemicalCompoundRepository and its implementation ChemicalCompoundRepositoryImpl together under the interfaces type.

See Additional file 1 for the full source code of ChemicalCompoundRepositoryImpl and AbstractStructureSearchRepositoryImpl which it extends.

As can be deduced from above source code, the chemical structure search methods support paging. Paging of search results has one main benefit: only the requested data needs to be loaded from the database. This can have a huge impact on performance especially if the structures are stored as molfiles. Paging requires that the total number of records is known. The need to count the total hits of a query for each page will greatly reduce performance. MDF solves this issue by caching the count of a query. Hence the count is only determined when the query is executed the first time. Subsequent requests for the next page use the cached value. In the event that relevant data is edited or added, the cache is cleared.

MDF contains a Spring data repository for each entity (ChemicalCompound, Containable, and ChemicalCompoundContainer) with methods for chemical structure searching. A developer must extend the provided repositories for each of his entity implementations and may add his own custom search methods to them.

### Service layer

The service layer is responsible for transaction management and security. It also offers methods for importing and exporting SD-Files. Transactions and security are implemented declaratively by using according annotations from Spring framework. Security is entirely optional. If you do not need it, MDF remains fully operational and every user can see everything.

### Service for ChemicalStructure entity

This service manages entities of type ChemicalStructure. This service is provided by the framework and should be used as-is. Any access of ChemicalStructures should be through this service. ChemicalStructureService contains the logic that makes ChemicalStructures immutable. For convenience ChemicalStructureService has a method that allows changing an existing ChemicalStructure. This will then affect all ChemicalCompoundCompositions that contain the changed ChemicalStructure.

### Services for ChemicalCompound and Containable

The services for ChemicalCompound and Containable are very similar. They consist of an interface which contains the security annotations and an implementation containing the @Transactional annotations for declarative transactions. They also offer methods with optional loading of lazy collections.

When an entity is saved, the service automatically sets the correct ChemicalStructures by either selecting an existing one from the database or creating a new one.

Before the entity is passed to Hibernate, the method preSave(entity) is executed. This method is empty in the provided abstract services like ChemicalCompoundServiceImpl but can be overridden in subclasses. For example one could set all non-nullable properties to a default or a sequence value if it is null.

For every ChemicalCompound and Containable implementation the application uses, a service interface and an implementation must be created. The services must implement the getRepository(), checkUniqueness() and getExistingCompound() methods.

### Service for ChemicalCompoundContainer

The main difference to services for ChemicalCompound and Containable is that an application should only have 1 implementation of ChemicalCompoundContainer and hence only one such service. The service can be used as provided or in some cases must be extended. When the ChemicalCompoundContainer implementation adds additional unique constraints, checkUniqueness() and getExistingContainer() must be overridden.

## Results and discussion

### Functionality

Below is a listing of the core functionality of MDF:

• Chemical structure search: Full, Sub, SMARTS, Similarity, Formula

• Chemical structure searches can be combined with property searches

• Chemical structure searches are paged and cached

• Support for multi-component compounds (mixtures)

• 3 chemical structure searchable entities: ChemicalCompound, Containable and ChemicalCompoundContainer

• Import and Export of SD-Files for above 3 entities

• Transactional database access

• Optional security (authorization)

With the design and functionality of MDF it is possible to build many different types of system such as registration systems, inventory systems or just a simple compound database. While you could also create your own ELN, there also exists the free Indigo ELN. This ELN was create by GGA Software Services and is used at Pfizer [7].

In contrast to MolDB5R [3] and to MyMolDB [4], MDF is not a fully functional standalone web application with chemical structure search. As the name implies it’s a framework to simplify creating such an application. MDF may also be used to create local or client-server desktop applications. MDF is targeted at software developers and not intended for use by scientists themselves. However MDF features are very robust. Chemical structure searching is done in the database and not the application code. Hence you can search by chemical structure and other properties at the same time, the results can be sorted by multiple properties and can be paged (SQL OFFSET and LIMIT clauses). Note that if you do the chemical structure search in application code, any query will require at least two trips to the database, namely the structure search and subsequently the filtering by other properties, sorting and/or limiting. Both need to happen in the same transaction. It was not determined if MolDB5R [3] and MyMolDB [4] actually do this in the same transaction.

In MDF the chemical compounds can be associated with a containable, which in registration systems would be a batch or in an inventory system a lot. A specific physically available sample in a bar-coded bottle can then be associated with a container. These containers can also be searched by chemical structure. This is the foundation for creating an inventory system. Developers may add as many additional properties as they want to each of the entities and all of them are searchable together with the chemical structure.

All data access in MDF is transactional to prevent data inconsistencies. MDF can be configured to use a database connection pool. When querying a RDBMS creating a connection often takes more time than the query itself and hence if you already have open connections response times can be reduced.

For similarity searching MDF exposed the algorithms provided by the Bingo Cartridge which are Tanimoto, Tversky and Euclidean metric for substructures.

MDF is ready to be used with Spring security. Security is optional. MDF offers method level security (authorization). It does not offer any authentication features.

### Mixture handling

MDF supports multi-component chemical compounds. Searching by substructure will return all compounds that have at least one component (chemical structure) matching the query structure. This is important because reaction products that may be entered into a chemical registration system are almost always mixtures unless extensive purification is done.

If an entry in an imported SD-File consists of multiple disconnected structures it is assumed that this entry is a mixture and each structure is stored as a separate chemical structure.

### Structure normalization

By default MDF stores the chemical structures as they are submitted. MDF does not do any standardization/normalization of chemical structures. It is up to the developer using MDF to ensure that chemical structures are correctly normalized prior to saving them to the database. It is currently suggested that developers implement such a feature by overriding the preSave() method of ChemicalCompoundServiceImpl. This method is called before any chemical compound is created or updated. Within this method the chemical compound and all the chemical structures it consists of can be freely manipulated as desired. Note that every compound being saved will be processed by this method.

### Salts, solvates and solutions

MDF current version 1.0.1 has no special handling for salts, solvates or solutions. MDF will store separate components in a chemical structure file as a separate chemical structure. Therefore saving a salt like [NH+]1 = CC = CC = C1.[Br-] will be represented as a mixture of the two ions without any percentages set. An exact structure search for either ion would return this salt. If the salt has a charge greater than 1 and multiple ions associated to it like [NH+]1 = CC = [NH+]C = C1.[Br-].[Br-] the salt will be stored as a mixture of [NH+]1 = CC = [NH+]C = C1 and [Br-] without any percentages set. If the chemical structure is a single ion it will be stored and searchable like any other chemical structure. If this behaviour is unsuitable in a specific case, developers may implement salt and solvate handling functionality in the preSave() method.

Some commercial systems also appear to have no way of handling solutions. It is recommended to create the compound as if it were pure and add the solution information as separate fields on the compound level.

### Example web application

**Figure 3:**
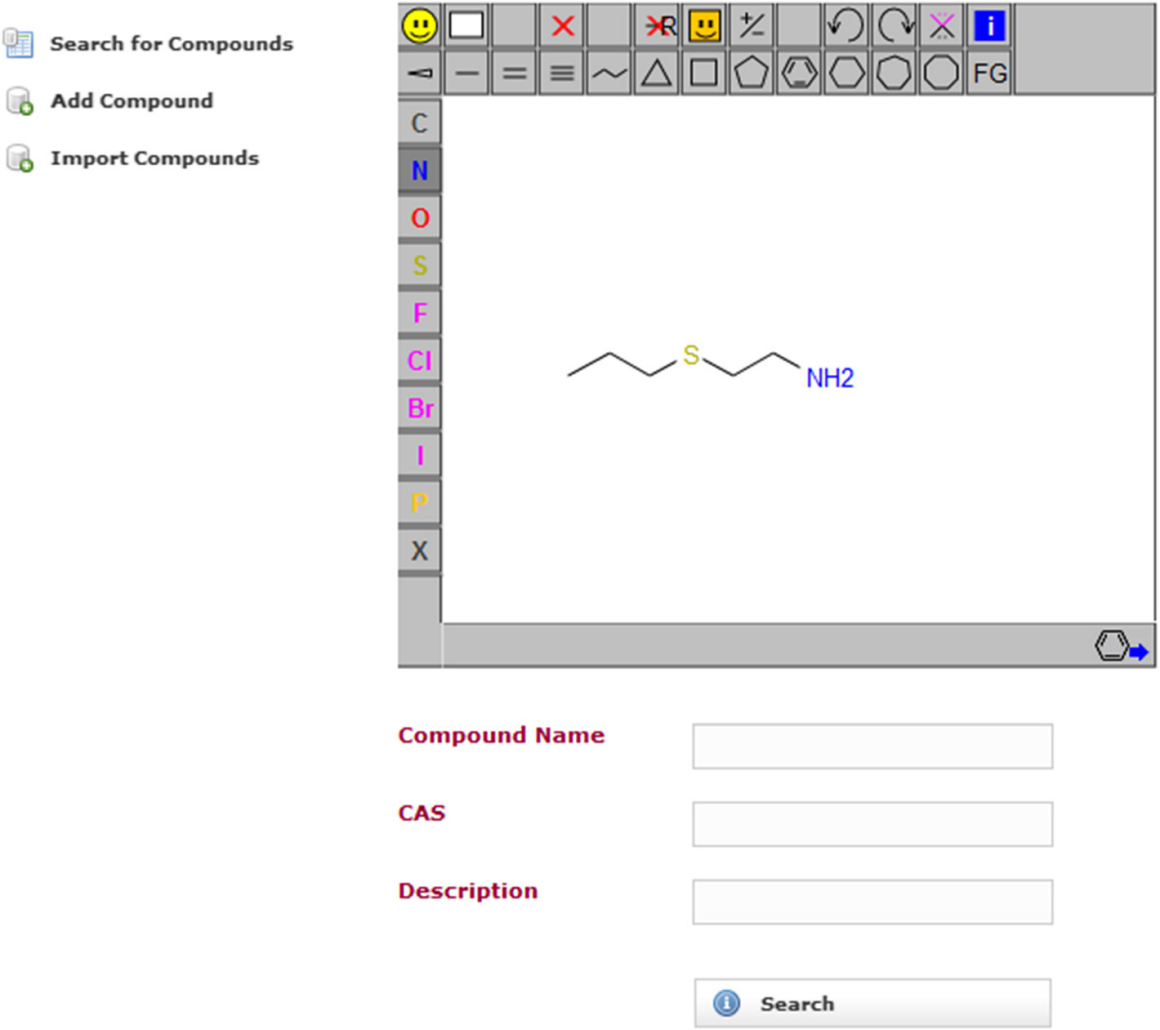
**Search page of the example web application using MDF.** A user can search the compound database by chemical substructure and/or properties like compound name or CAS number.

**Figure 4:**
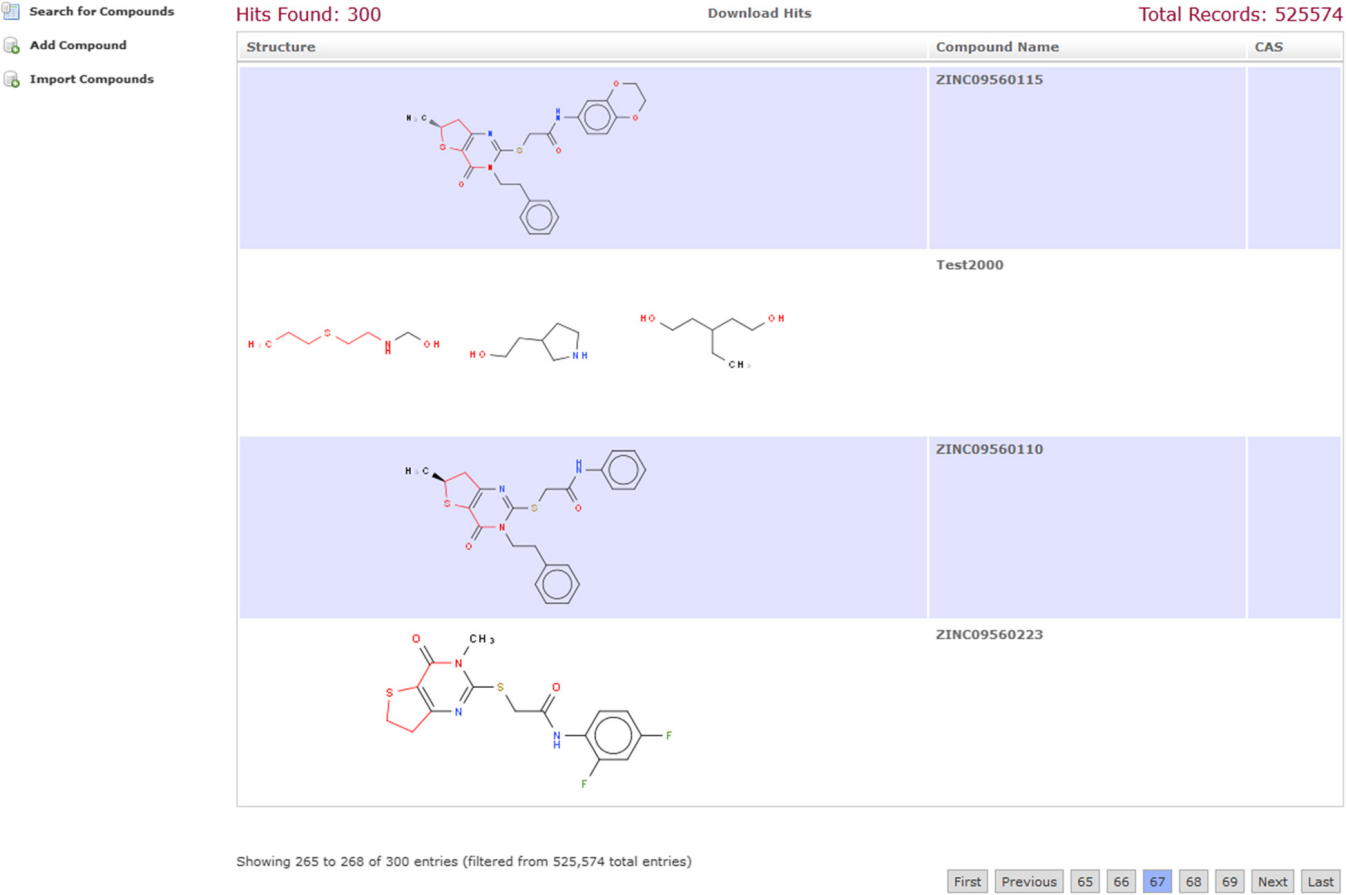
**Result page of a substructure search.** The results are displayed in a paged table generated by the JQuery plugin datatables [9]. The chemical structure images have the matching substructure highlighted in red. Clicking in the chemical structure image will display the SMILES for it.

**Figure 5:**
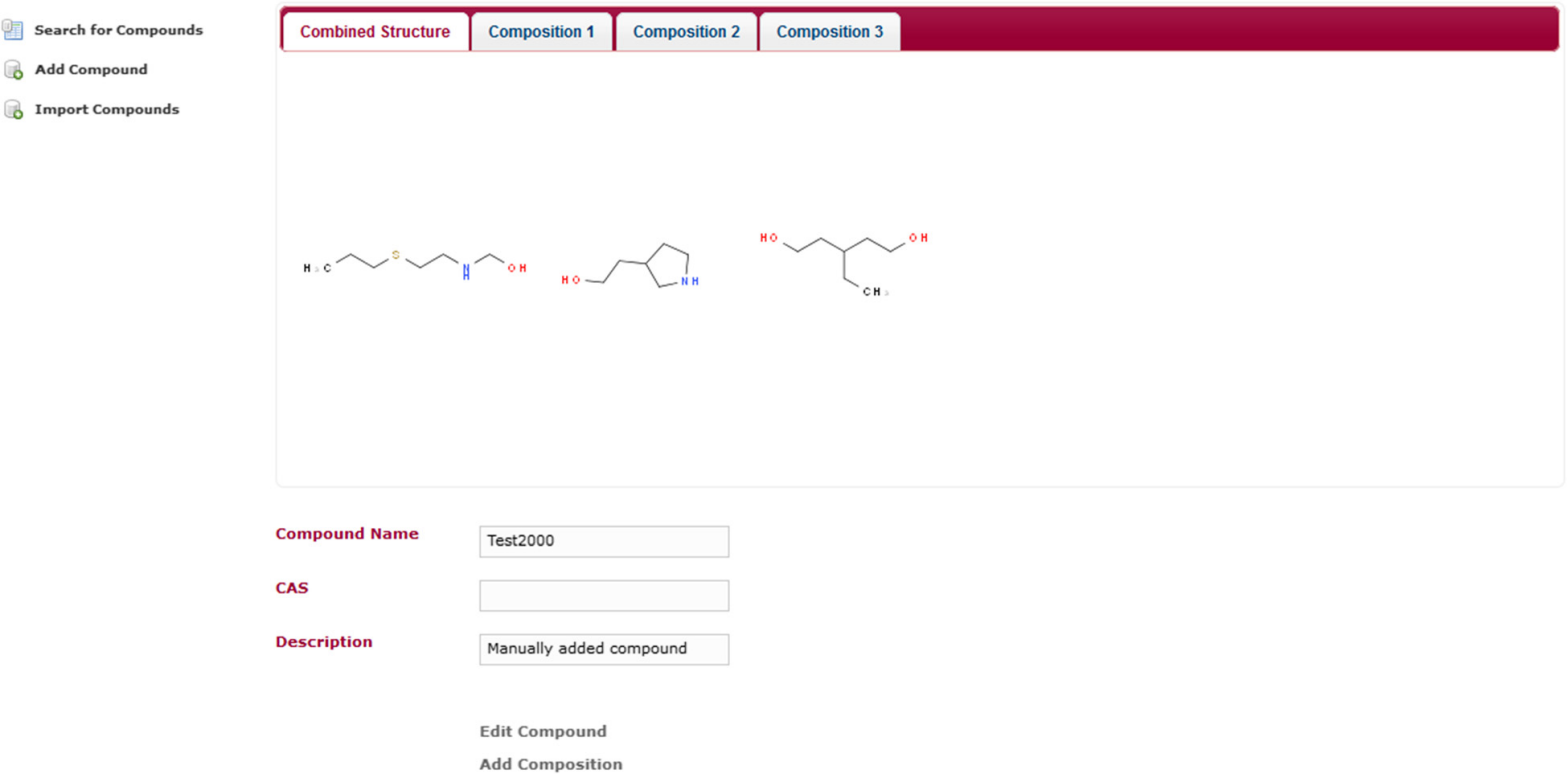
**Individual compound view.** This web page displays a single compound. The compound can be edited or deleted by clicking on the according link within the page. There is a tab displaying all contained chemical structures and a tab for each individual composition the compound consists of.

**Figure 6:**
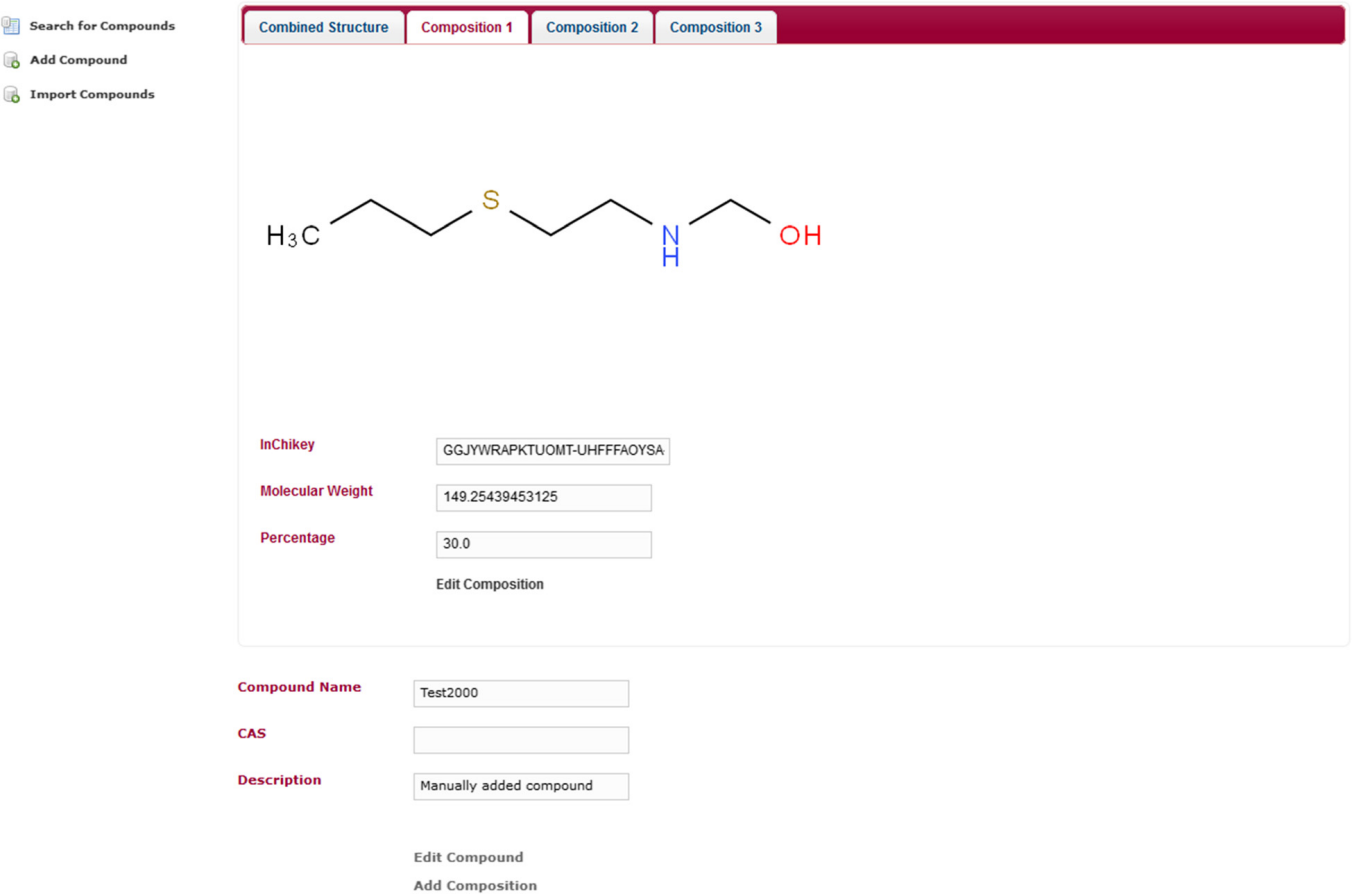
**Single composition.** Shows the same page as Figure 5 but instead of the Combined Structure tab, the tab of the first composition is selected. The composition can be edited by clicking on the according link within the page.

A simple web application using MDF was created. The web application makes use of Spring MVC. The application does not make use of the security integration and it does not use the entities Containable and ChemicalCompoundContainer. It only uses ChemicalCompound entity. The application is a compound database for multi-component compounds. It has a page for importing the chemical structures in an SD-file into the compound database. The database can be searched by substructure and properties. It uses JSME [8] for drawing the chemical structures (Figure 3). The search results page displays the search hits in a tabular and paged fashion. When a substructure search is done, the substructure will be highlighted in the search results (Figure 4). The hits of a search can be exported as an SD-file. The search results contain a link to a single compound view. The properties of the compound may be edited and compositions can be added, edited and deleted (Figures 5, 6). When editing a compound or a composition the application deals with concurrent modifications transparently and conflict resolution dialog is shown on which the user can select which values to use for each property and then save that new version.

### Performance

MDF has one main performance issue when handling mixtures. If an application uses mixtures, i.e. compounds with multiple components, a chemical structure query will return one row for every component in a compound matching the query. This is undesirable because the end users want to see each compound that matches the query only once. The solution to the problem is to use a distinct query and this is where the performance issue happens. If you perform a distinct query, the whole database needs to be searched regardless of limit clause which greatly increases execution time. Note that sorting has the same effect. So sorting can have a huge performance penalty too and when paging you should always sort to get a predictable outcome. To make this even worse Bingo Cartridge for PostgreSQL does not yet have a proper implementation for cost estimation and the cost for using the chemical structure index is hard coded and underestimated. This misleads the PostgreSQL query planer to always use a full index scan on structure search index even when the query has an additional where-clause that greatly limits the amount of results. In these cases it would, for example, be faster to use the index for the CAS number and use the Bingo matchsub function for filtering. The matchsub function does substructure matching without index. This is of course slower than with an index but if it only has to be done for a small number of structures it is a lot faster than the full index scan. To fix the cost estimation issue, MDF does some internal calculations to explicitly decide whether the structure index or the matchsub function is used. This can improve performance by an order of magnitude. Note that the supplier of the Bingo Cartridge is aware of this issue and the timeline for the fix was end of 2013. The main issue of distinct queries and sorting however is inherent to how relational databases work and cannot be solved except with a better substructure search index or better hardware. MDF also offers a setting to disable distinct queries application-wide for single-component compound databases.

To benchmark MDF the earlier outlined web application was used. The database contains 525573 unique compounds. The compounds are from Zinc [10] subset 13 at reference pH 7 the SD-files 13_p0.0.sdf, 13_p0.1.sdf, 13_p0.10.sdf and 13_p0.11.sdf. The structures are stored in the database as SMILES. Importing each of the SD-files, which contain approximately 131’000 chemical structures, took 12 min with the chemical structure search index disabled. Rebuilding the index after all SD-files were imported took 22 min on a laptop with 4 GB RAM, Core i5-3220M CPU and a 512GB Samsung 830 SSD. That totals to 1 h 10 min to setup a fully indexed database with half a million compounds. As an additional reference the same import was done on a desktop PC with 12 GB of RAM, i7-875K @ 3.4 GHz and the database running on a Western Digital Green Drive (5400 RPM). Here the import took 8 min and the conclusion is that the import is CPU limited rather than being limited by storage-speed. The index generation took approximately 22 min on the laptop and 20 min on the desktop. The conclusion here is that it is limited more by CPU but drive speed also matters. The import and index generation performance when storing the structures as molfiles was not benchmarked.

The substructure search performance was benchmarked with different configuration settings. Substructure search is done by Bingo PostgreSQL cartridge [5] and this benchmark therefore reflects its performance plus any overhead caused by MDF. With the exception of c1ccccc1 the author drew chemical structures with no specific significance and tested the search speed. The search speed was determined by logbacks [11] implementation of org.slf4j.profiler.Profiler.

The first benchmark is a reference. This benchmark used the option to disable all distinct queries and no sorting was done. MDF performs a count of total hits on first occurrence of a chemical structure search and the count is cached which causes the first page to load slower than subsequent pages. Each page contains 4 records. The results are shown in Table 2 ordered ascending by number of hits.

**Table 2:** Substructure search performance

**SMILES**	**First page (s)**	**Second page (s)**	**Hits**
OCC1CCN1	0.5	0.5	21
CCCC1CCOC1	0.6	0.5	815
CCNCCOCC	2.1	0.5	31802
CCCCCC	4.8	0.5	92788
c1ccccc1	19	0.5	420299

The benchmark was repeated but this time with distinct queries enabled. First page load time is doubled because the count query is run and then the actual query is run which takes about the same amount of time as the count query due to the distinct clause. The second page then always takes half the time to load compared to the first page for the same reason (Table 3). The number of hits is identical to the ones in Table 2 because all compounds in the database consist of only one component.

**Table 3:** Substructure search performance with distinct results

**SMILES**	**First page (s)**	**Second page (s)**	**Hits**
OCC1CCN1	0.6	0.4	21
CCCC1CCOC1	2.7	0.5	815
CCNCCOCC	6.2	2.1	31802
CCCCCC	9.3	4.8	92788
c1ccccc1	38	19	420299

The results show that Bingo does no optimizations for a common substructure query like a benzene ring and hence searching for c1ccccc1 in a database in which almost all molecules have this feature is very slow. To improve search speed in such a scenario filtering by additional properties is recommended. Hence the benchmark was repeated with an additional filter of compound name starting with “ZINC34”.

Table 4 shows the benefit of the MDF optimization as a workaround of the cost estimation issue in Bingo PostgreSQL cartridge. Without this optimization the benchmark would have the same performance as shown in Table 3.

**Table 4:** Substructure search with distinct query and additional filter ‘ZINC34%’ on compound name

**SMILES**	**First page (s)**	**Second page (s)**	**Hits**
OCC1CCN1	1.2	-	0
CCCC1CCOC1	1.3	-	1
CCNCCOCC	1.0	0.7	49
CCCCCC	1.2	0.6	198
c1ccccc1	4.2	0.6	981

MDF also uses Bingo Cartridges similarity searching functionality. Its performance was tested by searching for compounds with a similarity score of 0.9 using the Tanimoto similarity measure also known as Jaccard Index [12]. The results are shown in Table 5.

**Table 5:** Tanimoto similarity search performance: hits with at least 90% similarity

**SMILES**	**First page (s)**	**Second page (s)**	**Hits**
CC1(C)Cc2ccccc2/C(=C/C(=O)NC2CCCCC2)/N1	0.40	-	3
COc1ccc(NC([C@H]2CC(O[C@H]2C) = O) = O)cc1	0.40	0.32	11
C[C@H](CCC)C[C@@H](C[NH3+])c1cc[n]cc1	0.41	0.20	17

### Outlook

For generating the chemical structure depictions the Indigo toolkit is used. The toolkit can be configured to generate the structures in many ways including the coloring of heteroatoms, bond length and width and many more. Currently this is hard coded and cannot be adjusted by the user. A next step would be to expose these configurations options so they can be set through a Java properties file. Also the handling of salts and solvates must be implemented to make MDF usable in areas were such compounds are important.

To make use of MDF you must be able to program in Java and you will need basic knowledge about Spring framework and how to configure it. This limits the target audience. When using MDF you need to write some boiler-plate code and hence the next step would be to create additional tools to facilitate the usage of MDF like the automatic generation of entity classes and their repositories and services. These tools would need to be configurable so that a user can define the desired properties for each of the entities and the desired search methods. An option would be a maven plugin. Maven plugins can generate code like the metamodel creation done by the QueryDSL-maven plugin. Another option would be annotations that generate code on compile like Project Lombok does [13].

The final step would be to create a web application platform that lets administrators create new web applications with chemical structure search capability by simply entering the desired properties for the entities on a web form and clicking on a button. It is obvious that this would require a significant development effort.

## Conclusions

MDF successfully abstracts chemical structure data maintenance and chemical structure searching and lets software developers focus their efforts on other aspects of the application like the graphical user interface and usability. This was achieved by integrating well maintained and widely used open-source software components which offer features like dependency injection, declarative transactions, security or advanced chemical structure search features like tautomer or resonance structure search. MDF therefore offers a robust basis for creating database applications with chemical structure search capability.

## Availability and requirements

MDF source code is available as mercurial source control repository on MDF’s project page on bitbucket [14] or as Additional file 2. Besides the source code the project page also contains a Wiki [15] with much more detailed information and usage instructions for MDF. MDF is also available as a maven artefact on maven central [16] or for a jar file without dependencies see Additional file 3. Note that this is a framework and not a runnable application!

MDF is licensed under AGPL 3 [17].

MDF is written in Java 7 and was successfully tested in 32 and 64-Bit Windows 7. However all components are cross-platform and it should run on other operating systems too.

The source code for the example web application is also available as mercurial source control repository [18] or as Additional file 4. In the download section there is also a 7-zip archive of a standalone version [19] of this simple web application for Windows 64-bit. No installation is needed as the standalone version already contains a configured PostgreSQL database and the tomcat servlet container.

## Abbreviations

AGPL: Affero General Public License; API: Application programming interface; CRUD: Create read update delete; ELN: Electronic lab notebook; JPA: Java persistence API; JPQL: Java persistence query language; MDF: Molecule database framework; ORM: Object relational mapping; RDBMS: Relational database management system; SQL: Structured query language; UML: Unified modelling language; XML: Extensible markup language.

## Competing interests

The author declares that he has no competing interests.

## Authors’ information

JK has a M.Sc. in Biology from ETHZ and a MAS in Computer Science from FFHS. He is employed by Givaudan Schweiz AG as Research Associate.

## Supplementary Material

Additional file 1 Highlighted Source Code of AbstractStructureSearchRepositoryImpl and ChemicalCompoundRepositoryImpl. Click here for file

Additional file 2 Molecule Database Framework source code. Click here for file

Additional file 3 Molecule Database Framework jar file without dependencies. Click here for file

Additional file 4 MDF simple web application source code of the mercurial changeset 16f39f4e447b. Click here for file

## References

[B1] Martin E Monge A Duret JA Gualandi F Peitsch MC Pospisil P Building an R&D chemical registration system J Cheminf 2012 5 11 10.1186/1758-2946-4-11 PMC3430593 22650418

[B2] ChemAxon Compound Registrationhttp://www.chemaxon.com/products/compound-registration/

[B3] Haider N Functionality pattern matching as an efficient complementary structure/reaction search tool: an open-source approach Molecules 2010 5 5079 5092 10.3390/molecules15085079 20714286 PMC6257694

[B4] Xia B Tai ZF Gu YC Li BJ Ding LS Zhou Y MyMolDB: a micromolecular database solution with open source and free components J Comput Chem 2011 5 2942 2948 10.1002/jcc.21874 21728180

[B5] Pavlov D Rybalkin M Karulin B Bingo from SciTouch LLC: chemistry cartridge for Oracle database J Cheminf 2010 5 Suppl 1 F1 10.1186/1758-2946-2-S1-F1

[B6] Weininger D SMILES, a chemical language and information system. 1. Introduction to methodology and encoding rules J Chem Inf Model 1988 5 31 36 10.1021/ci00057a005

[B7] Indigo ELN: The Open-Source Chemistry Electronic Lab Notebookhttp://www.ggasoftware.com/opensource/indigo/eln

[B8] Bienfait B Ertl P JSME: a free molecule editor in JavaScript J Cheminf 2013 5 24 10.1186/1758-2946-5-24 PMC3662632 23694746

[B9] DataTables (table plug-in for jQuery) https://datatables.net/

[B10] Irwin JJ Shoichet BK ZINC - A free database of commercially available compounds for virtual screening J Chem Inf Model 2005 5 177 182 10.1021/ci049714+ 15667143 PMC1360656

[B11] Logbackhttp://logback.qos.ch

[B12] Jaccard P Distribution de la flore alpine dans le bassin des Dranses et dans quelques régions voisines Bull Soc Vaud Sci Nat 1901 5 241 272

[B13] Custom AST transformations with Project Lombokhttp://www.ibm.com/developerworks/library/j-lombok/

[B14] MDF – Source Codehttps://bitbucket.org/kienerj/moleculedatabaseframework/src

[B15] MDF – Wiki https://bitbucket.org/kienerj/moleculedatabaseframework/wiki/Home

[B16] Maven Central Repositoryhttp://search.maven.org/

[B17] GNU Affero General Public Licensehttp://www.gnu.org/licenses/agpl-3.0.html

[B18] MDFSimpleWebApphttps://bitbucket.org/kienerj/mdfsimplewebapp

[B19] MDFSimpleWebApp Standalone Downloadhttps://bitbucket.org/kienerj/mdfsimplewebapp/downloads

