# Supplementary material for: Molecule database framework: a framework for creating database applications with chemical structure search capability
Source: J Cheminform. 2013 Dec 11;5:48. doi: 10.1186/1758-2946-5-48 (PMC3892073; doi:10.1186/1758-2946-5-48)
Supplement: Additional file 1 — Highlighted Source Code of AbstractStructureSearchRepositoryImpl and ChemicalCompoundRepositoryImpl. [file 1758-2946-5-48-S1.html]

ChemicalCompoundRepositoryImpl Source Code
