# Supplementary material for: Molecule database framework: a framework for creating database applications with chemical structure search capability
Source: J Cheminform. 2013 Dec 11;5:48. doi: 10.1186/1758-2946-5-48 (PMC3892073; doi:10.1186/1758-2946-5-48)
Supplement: Additional file 4 — MDF simple web application source code of the mercurial changeset 16f39f4e447b. [file 1758-2946-5-48-S4.zip › src/main/webapp/resources/js/datatables/AutoFill/inputs.html]

AutoFill example


AutoFill example with input elements

# Preamble

AutoFill works with Input elements and Select elements, as well as plain HTML cells. This
example shows all inputs cells, combined with DataTables' DOM sorting plug-in. You can
even combine input and plain HTML cells if you wanted (useful from something like jEditable).

# Live example

| Rendering engine | Browser | Platform(s) | Engine version | CSS grade |
| --- | --- | --- | --- | --- |
| Rendering engine | Browser | Platform(s) | Engine version | CSS grade |
| --- | --- | --- | --- | --- |
|  |  |  |  |  |
|  |  |  |  |  |
|  |  |  |  |  |
|  |  |  |  |  |
|  |  |  |  |  |
|  |  |  |  |  |
|  |  |  |  |  |
|  |  |  |  |  |
|  |  |  |  |  |
|  |  |  |  |  |
|  |  |  |  |  |
|  |  |  |  |  |
|  |  |  |  |  |
|  |  |  |  |  |
|  |  |  |  |  |
|  |  |  |  |  |
|  |  |  |  |  |
|  |  |  |  |  |
|  |  |  |  |  |
|  |  |  |  |  |
|  |  |  |  |  |
|  |  |  |  |  |
|  |  |  |  |  |
|  |  |  |  |  |
|  |  |  |  |  |
|  |  |  |  |  |
|  |  |  |  |  |
|  |  |  |  |  |
|  |  |  |  |  |
|  |  |  |  |  |
|  |  |  |  |  |
|  |  |  |  |  |
|  |  |  |  |  |
|  |  |  |  |  |
|  |  |  |  |  |
|  |  |  |  |  |
|  |  |  |  |  |
|  |  |  |  |  |
|  |  |  |  |  |
|  |  |  |  |  |
|  |  |  |  |  |
|  |  |  |  |  |
|  |  |  |  |  |
|  |  |  |  |  |
|  |  |  |  |  |
|  |  |  |  |  |
|  |  |  |  |  |
|  |  |  |  |  |
|  |  |  |  |  |
|  |  |  |  |  |
|  |  |  |  |  |
|  |  |  |  |  |
|  |  |  |  |  |
|  |  |  |  |  |
|  |  |  |  |  |
|  |  |  |  |  |
|  |  |  |  |  |

# Examples

- Basic initialisation
- Selecting which columns to provide AutoFill on
- Scrolling a DataTable using fill
- Using with input elements
- Customisation using callback functions

# Initialisation code

```
$.fn.dataTableExt.afnSortData['dom-text'] = function ( oSettings, iColumn )
{
	var aData = [];
	$( 'td:eq('+iColumn+') input', oSettings.oApi._fnGetTrNodes(oSettings) ).each( function () {
		aData.push( this.value );
	} );
	return aData;
}

$(document).ready( function () {
	var oTable = $('#example').dataTable( {
		"aoColumnDefs": [
			{ "sSortDataType": "dom-text", "aTargets": [ "_all" ] },
			{ "sType": "numeric", "aTargets": [ -2 ] }
		]
	} );
	new AutoFill( oTable );
} );
```

AutoFill and DataTables © Allan Jardine 2009-2010.
