# Supplementary material for: Molecule database framework: a framework for creating database applications with chemical structure search capability
Source: J Cheminform. 2013 Dec 11;5:48. doi: 10.1186/1758-2946-5-48 (PMC3892073; doi:10.1186/1758-2946-5-48)
Supplement: Additional file 4 — MDF simple web application source code of the mercurial changeset 16f39f4e447b. [file 1758-2946-5-48-S4.zip › src/main/webapp/resources/js/datatables/AutoFill/media/docs/global.html]

Global - documentation


# Global

## Navigation

- Overview
- Summary

  Properties | Methods
- Details

  Properties | Methods

Hiding private elements
(toggle)

Showing extended elements
(toggle)

## Summary

### Properties

<constant> CLASS :String
:   Name of this class

<constant> VERSION :String
:   AutoFill version

### Methods

fnSettings() → {object}
:   Retreieve the settings object from an instance

\_fnFillerDisplay(e)
:   Display the drag handle on mouse over cell

\_fnFillerDragMove(e)
:   Mouse move event handler for during a move. See if we want to update the display based on the
    new cursor position

\_fnFillerDragStart(e)
:   Mouse down event handler for starting a drag

\_fnFillerFinish(e)
:   Mouse release handler - end the drag and take action to update the cells with the needed values

\_fnFillerPosition(nTd)
:   Position the filler icon over a cell

\_fnInit(oDT, oConfig)
:   Initialisation

\_fnPrep(sStr) → {Object}
:   Chunk a string such that it can be filled in by the stepper function

\_fnReadCell(nTd) → {String}
:   Read informaiton from a cell, possibly using live DOM elements if suitable

\_fnStep(nTd, oPrepped, iDiff, bIncrement, sToken) → {String}
:   Render a string for it's position in the table after the drag (incrememt numbers)

\_fnTargetCoords(nTd) → {Object}
:   Find out the coordinates of a given TD cell in a table

\_fnUpdateBorder(nStart, nEnd)
:   Display the border around one or more cells (from start to end)

\_fnWriteCell(nTd, sVal, bLast)
:   Write informaiton to a cell, possibly using live DOM elements if suitable

## Details

### Properties

<constant> CLASS :String
:   Name of this class

<constant> VERSION :String
:   AutoFill version

### Methods

fnSettings() → {object}
:   Retreieve the settings object from an instance

    ##### Returns:

    AutoFill settings object

    \_fnFillerDisplay(e)
    :   Display the drag handle on mouse over cell

        ##### Parameters:

        |  | Name | Type | Attributes | Default | Description |
        | --- | --- | --- | --- | --- | --- |
        | 1 | e | Object |  |  | Event object |

        ##### Returns:

        void

        \_fnFillerDragMove(e)
        :   Mouse move event handler for during a move. See if we want to update the display based on the
            new cursor position

            ##### Parameters:

            |  | Name | Type | Attributes | Default | Description |
            | --- | --- | --- | --- | --- | --- |
            | 1 | e | Object |  |  | Event object |

            ##### Returns:

            void

            \_fnFillerDragStart(e)
            :   Mouse down event handler for starting a drag

                ##### Parameters:

                |  | Name | Type | Attributes | Default | Description |
                | --- | --- | --- | --- | --- | --- |
                | 1 | e | Object |  |  | Event object |

                ##### Returns:

                void

                \_fnFillerFinish(e)
                :   Mouse release handler - end the drag and take action to update the cells with the needed values

                    ##### Parameters:

                    |  | Name | Type | Attributes | Default | Description |
                    | --- | --- | --- | --- | --- | --- |
                    | 1 | e | Object |  |  | Event object |

                    ##### Returns:

                    void

                    \_fnFillerPosition(nTd)
                    :   Position the filler icon over a cell

                        ##### Parameters:

                        |  | Name | Type | Attributes | Default | Description |
                        | --- | --- | --- | --- | --- | --- |
                        | 1 | nTd | Node |  |  | Cell to position filler icon over |

                        ##### Returns:

                        void

                        \_fnInit(oDT, oConfig)
                        :   Initialisation

                            ##### Parameters:

                            |  | Name | Type | Attributes | Default | Description |
                            | --- | --- | --- | --- | --- | --- |
                            | 1 | oDT | object |  |  | DataTables settings object |
                            | 2 | oConfig | object |  |  | Configuration object for AutoFill |

                            ##### Returns:

                            void

                            \_fnPrep(sStr) → {Object}
                            :   Chunk a string such that it can be filled in by the stepper function

                                ##### Parameters:

                                |  | Name | Type | Attributes | Default | Description |
                                | --- | --- | --- | --- | --- | --- |
                                | 1 | sStr | String |  |  | String to prep |

                                ##### Returns:

                                with parameters, iStart, sStr and sPostFix

                                \_fnReadCell(nTd) → {String}
                                :   Read informaiton from a cell, possibly using live DOM elements if suitable

                                    ##### Parameters:

                                    |  | Name | Type | Attributes | Default | Description |
                                    | --- | --- | --- | --- | --- | --- |
                                    | 1 | nTd | Node |  |  | Cell to read |

                                    ##### Returns:

                                    Read value

                                    \_fnStep(nTd, oPrepped, iDiff, bIncrement, sToken) → {String}
                                    :   Render a string for it's position in the table after the drag (incrememt numbers)

                                        ##### Parameters:

                                        |  | Name | Type | Attributes | Default | Description |
                                        | --- | --- | --- | --- | --- | --- |
                                        | 1 | nTd | Node |  |  | Cell being written to |
                                        | 2 | oPrepped | Object |  |  | Prepared object for the stepper (from \_fnPrep) |
                                        | 3 | iDiff | Int |  |  | Step difference |
                                        | 4 | bIncrement | Boolean |  |  | Increment (true) or decriment (false) |
                                        | 5 | sToken | String |  |  | Token to replace |

                                        ##### Returns:

                                        Rendered information

                                        \_fnTargetCoords(nTd) → {Object}
                                        :   Find out the coordinates of a given TD cell in a table

                                            ##### Parameters:

                                            |  | Name | Type | Attributes | Default | Description |
                                            | --- | --- | --- | --- | --- | --- |
                                            | 1 | nTd | Node |  |  | undefined |

                                            ##### Returns:

                                            x and y properties, for the position of the cell in the tables DOM

                                            \_fnUpdateBorder(nStart, nEnd)
                                            :   Display the border around one or more cells (from start to end)

                                                ##### Parameters:

                                                |  | Name | Type | Attributes | Default | Description |
                                                | --- | --- | --- | --- | --- | --- |
                                                | 1 | nStart | Node |  |  | Starting cell |
                                                | 2 | nEnd | Node |  |  | Ending cell |

                                                ##### Returns:

                                                void

                                                \_fnWriteCell(nTd, sVal, bLast)
                                                :   Write informaiton to a cell, possibly using live DOM elements if suitable

                                                    ##### Parameters:

                                                    |  | Name | Type | Attributes | Default | Description |
                                                    | --- | --- | --- | --- | --- | --- |
                                                    | 1 | nTd | Node |  |  | Cell to write |
                                                    | 2 | sVal | String |  |  | Value to write |
                                                    | 3 | bLast | Boolean |  |  | Flag to show if this is that last update |

                                                    ##### Returns:

                                                    void

Documentation generated by JSDoc 3 on
22th Jun 2012 - 08:22
with the DataTables template.
