# Supplementary material for: Molecule database framework: a framework for creating database applications with chemical structure search capability
Source: J Cheminform. 2013 Dec 11;5:48. doi: 10.1186/1758-2946-5-48 (PMC3892073; doi:10.1186/1758-2946-5-48)
Supplement: Additional file 4 — MDF simple web application source code of the mercurial changeset 16f39f4e447b. [file 1758-2946-5-48-S4.zip › src/main/webapp/resources/js/datatables/AutoFill/scrolling.html]

AutoFill example


AutoFill example with scrolling

# Preamble

When dragging an AutoFill handle, the table (if DataTables scrolling is enabled) or the window will be automatically scrolled, as you approach the edge of the scrolling component. The example below shows the effect with DataTables scrolling (and also window if needed).

# Live example

| Rendering engine | Browser | Platform(s) | Engine version | CSS grade |
| --- | --- | --- | --- | --- |
| Rendering engine | Browser | Platform(s) | Engine version | CSS grade |
| --- | --- | --- | --- | --- |
| Trident | Internet Explorer 4.0 | Win 95+ (Entity: &) | 4 | X |
| Trident | Internet Explorer 5.0 | Win 95+ | 5 | C |
| Trident | Internet Explorer 5.5 | Win 95+ | 5.5 | A |
| Trident | Internet Explorer 6 | Win 98+ | 6 | A |
| Trident | Internet Explorer 7 | Win XP SP2+ | 7 | A |
| Trident | AOL browser (AOL desktop) | Win XP | 6 | A |
| Gecko (UTF-8: $¢€) | Firefox 1.0 | Win 98+ / OSX.2+ | 1.7 | A |
| Gecko | Firefox 1.5 | Win 98+ / OSX.2+ | 1.8 | A |
| Gecko | Firefox 2.0 | Win 98+ / OSX.2+ | 1.8 | A |
| Gecko | Firefox 3.0 | Win 2k+ / OSX.3+ | 1.9 | A |
| Gecko | Camino 1.0 | OSX.2+ | 1.8 | A |
| Gecko | Camino 1.5 | OSX.3+ | 1.8 | A |
| Gecko | Netscape 7.2 | Win 95+ / Mac OS 8.6-9.2 | 1.7 | A |
| Gecko | Netscape Browser 8 | Win 98SE+ | 1.7 | A |
| Gecko | Netscape Navigator 9 | Win 98+ / OSX.2+ | 1.8 | A |
| Gecko | Mozilla 1.0 | Win 95+ / OSX.1+ | 1 | A |
| Gecko | Mozilla 1.1 | Win 95+ / OSX.1+ | 1.1 | A |
| Gecko | Mozilla 1.2 | Win 95+ / OSX.1+ | 1.2 | A |
| Gecko | Mozilla 1.3 | Win 95+ / OSX.1+ | 1.3 | A |
| Gecko | Mozilla 1.4 | Win 95+ / OSX.1+ | 1.4 | A |
| Gecko | Mozilla 1.5 | Win 95+ / OSX.1+ | 1.5 | A |
| Gecko | Mozilla 1.6 | Win 95+ / OSX.1+ | 1.6 | A |
| Gecko | Mozilla 1.7 | Win 98+ / OSX.1+ | 1.7 | A |
| Gecko | Mozilla 1.8 | Win 98+ / OSX.1+ | 1.8 | A |
| Gecko | Seamonkey 1.1 | Win 98+ / OSX.2+ | 1.8 | A |
| Gecko | Epiphany 2.20 | Gnome | 1.8 | A |
| Webkit | Safari 1.2 | OSX.3 | 125.5 | A |
| Webkit | Safari 1.3 | OSX.3 | 312.8 | A |
| Webkit | Safari 2.0 | OSX.4+ | 419.3 | A |
| Webkit | Safari 3.0 | OSX.4+ | 522.1 | A |
| Webkit | OmniWeb 5.5 | OSX.4+ | 420 | A |
| Webkit | iPod Touch / iPhone | iPod | 420.1 | A |
| Webkit | S60 | S60 | 413 | A |
| Presto | Opera 7.0 | Win 95+ / OSX.1+ | - | A |
| Presto | Opera 7.5 | Win 95+ / OSX.2+ | - | A |
| Presto | Opera 8.0 | Win 95+ / OSX.2+ | - | A |
| Presto | Opera 8.5 | Win 95+ / OSX.2+ | - | A |
| Presto | Opera 9.0 | Win 95+ / OSX.3+ | - | A |
| Presto | Opera 9.2 | Win 88+ / OSX.3+ | - | A |
| Presto | Opera 9.5 | Win 88+ / OSX.3+ | - | A |
| Presto | Opera for Wii | Wii | - | A |
| Presto | Nokia N800 | N800 | - | A |
| Presto | Nintendo DS browser | Nintendo DS | 8.5 | C/A1 |
| KHTML | Konqureror 3.1 | KDE 3.1 | 3.1 | C |
| KHTML | Konqureror 3.3 | KDE 3.3 | 3.3 | A |
| KHTML | Konqureror 3.5 | KDE 3.5 | 3.5 | A |
| Tasman | Internet Explorer 4.5 | Mac OS 8-9 | - | X |
| Tasman | Internet Explorer 5.1 | Mac OS 7.6-9 | 1 | C |
| Tasman | Internet Explorer 5.2 | Mac OS 8-X | 1 | C |
| Misc | NetFront 3.1 | Embedded devices | - | C |
| Misc | NetFront 3.4 | Embedded devices | - | A |
| Misc | Dillo 0.8 | Embedded devices | - | X |
| Misc | Links | Text only | - | X |
| Misc | Lynx | Text only | - | X |
| Misc | IE Mobile | Windows Mobile 6 | - | C |
| Misc | PSP browser | PSP | - | C |
| Other browsers | All others | - | - | U |

# Examples

- Basic initialisation
- Selecting which columns to provide AutoFill on
- Scrolling a DataTable using fill
- Using with input elements
- Customisation using callback functions

# Initialisation code

```
$(document).ready( function () {
	var oTable = $('#example').dataTable({
		"sScrollY": 200,
		"bScrollCollapse": true,
		"bPaginate": false
	});
	new AutoFill( oTable );
} );
```

AutoFill and DataTables © Allan Jardine 2009-2010.
