# Supplementary material for: Molecule database framework: a framework for creating database applications with chemical structure search capability
Source: J Cheminform. 2013 Dec 11;5:48. doi: 10.1186/1758-2946-5-48 (PMC3892073; doi:10.1186/1758-2946-5-48)
Supplement: Additional file 4 — MDF simple web application source code of the mercurial changeset 16f39f4e447b. [file 1758-2946-5-48-S4.zip › src/main/webapp/resources/js/datatables/ColReorder/fixedcolumns.html]

ColReorder example


ColReorder example with FixedColumns

# Preamble

While ColReorder works great with scrolling in DataTables (and thus FixedColumns),
it also presents an additional option called 'iFixedColumns' which allows you to not
let the user reorder certain columns (specific the number given, counting left to
right). So in the case of FixedColumns this is useful because you typically won't want
to let your fixed column be reordered. This is shown below in the FixedColumns index column
example.

# Live example

|  | Rendering engine | Browser | Platform(s) | Engine version | CSS grade |
| --- | --- | --- | --- | --- | --- |
| 1 | Trident | Internet Explorer 4.0 | Win 95+ | 4 | X |
| 2 | Trident | Internet Explorer 5.0 | Win 95+ | 5 | C |
| 3 | Trident | Internet Explorer 5.5 | Win 95+ | 5.5 | A |
| 4 | Trident | Internet Explorer 6 | Win 98+ | 6 | A |
| 5 | Trident | Internet Explorer 7 | Win XP SP2+ | 7 | A |
| 6 | Trident | AOL browser (AOL desktop) | Win XP | 6 | A |
| 7 | Gecko | Firefox 1.0 | Win 98+ / OSX.2+ | 1.7 | A |
| 8 | Gecko | Firefox 1.5 | Win 98+ / OSX.2+ | 1.8 | A |
| 9 | Gecko | Firefox 2.0 | Win 98+ / OSX.2+ | 1.8 | A |
| 10 | Gecko | Firefox 3.0 | Win 2k+ / OSX.3+ | 1.9 | A |
| 11 | Gecko | Camino 1.0 | OSX.2+ | 1.8 | A |
| 12 | Gecko | Camino 1.5 | OSX.3+ | 1.8 | A |
| 13 | Gecko | Netscape 7.2 | Win 95+ / Mac OS 8.6-9.2 | 1.7 | A |
| 14 | Gecko | Netscape Browser 8 | Win 98SE+ | 1.7 | A |
| 15 | Gecko | Netscape Navigator 9 | Win 98+ / OSX.2+ | 1.8 | A |
| 16 | Gecko | Mozilla 1.0 | Win 95+ / OSX.1+ | 1 | A |
| 17 | Gecko | Mozilla 1.1 | Win 95+ / OSX.1+ | 1.1 | A |
| 18 | Gecko | Mozilla 1.2 | Win 95+ / OSX.1+ | 1.2 | A |
| 19 | Gecko | Mozilla 1.3 | Win 95+ / OSX.1+ | 1.3 | A |
| 20 | Gecko | Mozilla 1.4 | Win 95+ / OSX.1+ | 1.4 | A |
| 21 | Gecko | Mozilla 1.5 | Win 95+ / OSX.1+ | 1.5 | A |
| 22 | Gecko | Mozilla 1.6 | Win 95+ / OSX.1+ | 1.6 | A |
| 23 | Gecko | Mozilla 1.7 | Win 98+ / OSX.1+ | 1.7 | A |
| 24 | Gecko | Mozilla 1.8 | Win 98+ / OSX.1+ | 1.8 | A |
| 25 | Gecko | Seamonkey 1.1 | Win 98+ / OSX.2+ | 1.8 | A |
| 26 | Gecko | Epiphany 2.20 | Gnome | 1.8 | A |
| 27 | Webkit | Safari 1.2 | OSX.3 | 125.5 | A |
| 28 | Webkit | Safari 1.3 | OSX.3 | 312.8 | A |
| 29 | Webkit | Safari 2.0 | OSX.4+ | 419.3 | A |
| 30 | Webkit | Safari 3.0 | OSX.4+ | 522.1 | A |
| 31 | Webkit | OmniWeb 5.5 | OSX.4+ | 420 | A |
| 32 | Webkit | iPod Touch / iPhone | iPod | 420.1 | A |
| 33 | Webkit | S60 | S60 | 413 | A |
| 34 | Presto | Opera 7.0 | Win 95+ / OSX.1+ | - | A |
| 35 | Presto | Opera 7.5 | Win 95+ / OSX.2+ | - | A |
| 36 | Presto | Opera 8.0 | Win 95+ / OSX.2+ | - | A |
| 37 | Presto | Opera 8.5 | Win 95+ / OSX.2+ | - | A |
| 38 | Presto | Opera 9.0 | Win 95+ / OSX.3+ | - | A |
| 39 | Presto | Opera 9.2 | Win 88+ / OSX.3+ | - | A |
| 40 | Presto | Opera 9.5 | Win 88+ / OSX.3+ | - | A |
| 41 | Presto | Opera for Wii | Wii | - | A |
| 42 | Presto | Nokia N800 | N800 | - | A |
| 43 | Presto | Nintendo DS browser | Nintendo DS | 8.5 | C/A1 |
| 44 | KHTML | Konqureror 3.1 | KDE 3.1 | 3.1 | C |
| 45 | KHTML | Konqureror 3.3 | KDE 3.3 | 3.3 | A |
| 46 | KHTML | Konqureror 3.5 | KDE 3.5 | 3.5 | A |
| 47 | Tasman | Internet Explorer 4.5 | Mac OS 8-9 | - | X |
| 48 | Tasman | Internet Explorer 5.1 | Mac OS 7.6-9 | 1 | C |
| 49 | Tasman | Internet Explorer 5.2 | Mac OS 8-X | 1 | C |
| 50 | Misc | NetFront 3.1 | Embedded devices | - | C |
| 51 | Misc | NetFront 3.4 | Embedded devices | - | A |
| 52 | Misc | Dillo 0.8 | Embedded devices | - | X |
| 53 | Misc | Links | Text only | - | X |
| 54 | Misc | Lynx | Text only | - | X |
| 55 | Misc | IE Mobile | Windows Mobile 6 | - | C |
| 56 | Misc | PSP browser | PSP | - | C |
| 57 | Other browsers | All others | - | - | U |
|  | Rendering engine | Browser | Platform(s) | Engine version | CSS grade |
| --- | --- | --- | --- | --- | --- |

# Examples

- Basic initialisation
- Styling the insert cursor
- Individual column filtering
- Integration with DataTables' ColVis plug-in
- Integration with DataTables' FixedColumns plug-in
- Integration with DataTables' FixedHeader plug-in
- Using a predefined column order set
- Providing a user control to reset the column order
- Column reordering shown with scrolling in DataTables
- Server-side processing support
- State saving of the column position
- jQuery UI theme integration

# Initialisation code

```
$(document).ready( function () {
	var oTable = $('#example').dataTable( {
		"sDom": 'Rlfrtip',
		"sScrollX": "100%",
		"sScrollXInner": "150%",
		"bScrollCollapse": true,
		"fnDrawCallback": function ( oSettings ) {
			/* Need to redo the counters if filtered or sorted */
			if ( oSettings.bSorted || oSettings.bFiltered ) {
				for ( var i=0, iLen=oSettings.aiDisplay.length ; i<iLen ; i++ ) {
					$('td:eq(0)', oSettings.aoData[ oSettings.aiDisplay[i] ].nTr ).html( i+1 );
				}
			}
		},
		"aoColumnDefs": [
			{ "bSortable": false, "sClass": "index", "aTargets": [ 0 ] }
		],
		"aaSorting": [[ 1, 'asc' ]],
		"oColReorder": {
			"iFixedColumns": 1
		}
	} );
	new FixedColumns( oTable );
} );
```

ColReorder and DataTables © Allan Jardine 2010
