# Supplementary material for: Molecule database framework: a framework for creating database applications with chemical structure search capability
Source: J Cheminform. 2013 Dec 11;5:48. doi: 10.1186/1758-2946-5-48 (PMC3892073; doi:10.1186/1758-2946-5-48)
Supplement: Additional file 4 — MDF simple web application source code of the mercurial changeset 16f39f4e447b. [file 1758-2946-5-48-S4.zip › src/main/webapp/resources/js/datatables/ColReorder/index.html]

ColReorder example


ColReorder example

# Preamble

This example shows the basic use case of the ColReorder plug-in. With ColReorder enabled
for a table, the user has the ability to click and drag any table header cell, and drop
it where they wish the column to be inserted. The insert point is shown visually, and
the column reordering is done as soon as the mouse button is released.

# Live example

| Rendering engine | Browser | Platform(s) | Engine version | CSS grade |
| --- | --- | --- | --- | --- |
| Rendering engine | Browser | Platform(s) | Engine version | CSS grade |
| --- | --- | --- | --- | --- |
| Trident | Internet Explorer 4.0 | Win 95+ (Entity: &) | 4 | X |
| Trident | Internet Explorer 5.0 | Win 95+ | 5 | C |
| Trident | Internet Explorer 5.5 | Win 95+ | 5.5 | A |
| Trident | Internet Explorer 6 | Win 98+ | 6 | A |
| Trident | Internet Explorer 7 | Win XP SP2+ | 7 | A |
| Trident | AOL browser (AOL desktop) | Win XP | 6 | A |
| Gecko (UTF-8: $¢€) | Firefox 1.0 | Win 98+ / OSX.2+ | 1.7 | A |
| Gecko | Firefox 1.5 | Win 98+ / OSX.2+ | 1.8 | A |
| Gecko | Firefox 2.0 | Win 98+ / OSX.2+ | 1.8 | A |
| Gecko | Firefox 3.0 | Win 2k+ / OSX.3+ | 1.9 | A |
| Gecko | Camino 1.0 | OSX.2+ | 1.8 | A |
| Gecko | Camino 1.5 | OSX.3+ | 1.8 | A |
| Gecko | Netscape 7.2 | Win 95+ / Mac OS 8.6-9.2 | 1.7 | A |
| Gecko | Netscape Browser 8 | Win 98SE+ | 1.7 | A |
| Gecko | Netscape Navigator 9 | Win 98+ / OSX.2+ | 1.8 | A |
| Gecko | Mozilla 1.0 | Win 95+ / OSX.1+ | 1 | A |
| Gecko | Mozilla 1.1 | Win 95+ / OSX.1+ | 1.1 | A |
| Gecko | Mozilla 1.2 | Win 95+ / OSX.1+ | 1.2 | A |
| Gecko | Mozilla 1.3 | Win 95+ / OSX.1+ | 1.3 | A |
| Gecko | Mozilla 1.4 | Win 95+ / OSX.1+ | 1.4 | A |
| Gecko | Mozilla 1.5 | Win 95+ / OSX.1+ | 1.5 | A |
| Gecko | Mozilla 1.6 | Win 95+ / OSX.1+ | 1.6 | A |
| Gecko | Mozilla 1.7 | Win 98+ / OSX.1+ | 1.7 | A |
| Gecko | Mozilla 1.8 | Win 98+ / OSX.1+ | 1.8 | A |
| Gecko | Seamonkey 1.1 | Win 98+ / OSX.2+ | 1.8 | A |
| Gecko | Epiphany 2.20 | Gnome | 1.8 | A |
| Webkit | Safari 1.2 | OSX.3 | 125.5 | A |
| Webkit | Safari 1.3 | OSX.3 | 312.8 | A |
| Webkit | Safari 2.0 | OSX.4+ | 419.3 | A |
| Webkit | Safari 3.0 | OSX.4+ | 522.1 | A |
| Webkit | OmniWeb 5.5 | OSX.4+ | 420 | A |
| Webkit | iPod Touch / iPhone | iPod | 420.1 | A |
| Webkit | S60 | S60 | 413 | A |
| Presto | Opera 7.0 | Win 95+ / OSX.1+ | - | A |
| Presto | Opera 7.5 | Win 95+ / OSX.2+ | - | A |
| Presto | Opera 8.0 | Win 95+ / OSX.2+ | - | A |
| Presto | Opera 8.5 | Win 95+ / OSX.2+ | - | A |
| Presto | Opera 9.0 | Win 95+ / OSX.3+ | - | A |
| Presto | Opera 9.2 | Win 88+ / OSX.3+ | - | A |
| Presto | Opera 9.5 | Win 88+ / OSX.3+ | - | A |
| Presto | Opera for Wii | Wii | - | A |
| Presto | Nokia N800 | N800 | - | A |
| Presto | Nintendo DS browser | Nintendo DS | 8.5 | C/A1 |
| KHTML | Konqureror 3.1 | KDE 3.1 | 3.1 | C |
| KHTML | Konqureror 3.3 | KDE 3.3 | 3.3 | A |
| KHTML | Konqureror 3.5 | KDE 3.5 | 3.5 | A |
| Tasman | Internet Explorer 4.5 | Mac OS 8-9 | - | X |
| Tasman | Internet Explorer 5.1 | Mac OS 7.6-9 | 1 | C |
| Tasman | Internet Explorer 5.2 | Mac OS 8-X | 1 | C |
| Misc | NetFront 3.1 | Embedded devices | - | C |
| Misc | NetFront 3.4 | Embedded devices | - | A |
| Misc | Dillo 0.8 | Embedded devices | - | X |
| Misc | Links | Text only | - | X |
| Misc | Lynx | Text only | - | X |
| Misc | IE Mobile | Windows Mobile 6 | - | C |
| Misc | PSP browser | PSP | - | C |
| Other browsers | All others | - | - | U |

# Examples

- Basic initialisation
- Styling the insert cursor
- Individual column filtering
- Integration with DataTables' ColVis plug-in
- Integration with DataTables' FixedColumns plug-in
- Integration with DataTables' FixedHeader plug-in
- Using a predefined column order set
- Providing a user control to reset the column order
- Column reordering shown with scrolling in DataTables
- Server-side processing support
- State saving of the column position
- jQuery UI theme integration

# Initialisation code

```
$(document).ready( function () {
	var oTable = $('#example').dataTable( {
		"sDom": 'Rlfrtip'
	} );
} );
```

ColReorder and DataTables © Allan Jardine 2010
