# Supplementary material for: Molecule database framework: a framework for creating database applications with chemical structure search capability
Source: J Cheminform. 2013 Dec 11;5:48. doi: 10.1186/1758-2946-5-48 (PMC3892073; doi:10.1186/1758-2946-5-48)
Supplement: Additional file 4 — MDF simple web application source code of the mercurial changeset 16f39f4e447b. [file 1758-2946-5-48-S4.zip › src/main/webapp/resources/js/datatables/ColReorder/media/docs/ColReorder.html]

Class: ColReorder - documentation


# Class: ColReorder

ColReorder

## Navigation

- Overview
- Summary

  Properties | Methods
- Details

  Properties | Methods

Hiding private elements
(toggle)

Showing extended elements
(toggle)

new ColReorder(DataTables, ColReorder)
:   ColReorder provides column visiblity control for DataTables

    ### Constructor

    ##### Parameters:

    |  | Name | Type | Attributes | Default | Description |
    | --- | --- | --- | --- | --- | --- |
    | 1 | DataTables | object |  |  | object |
    | 2 | ColReorder | object |  |  | options |

## Summary

### Properties

<static> aoInstances :array
:   Array of all ColReorder instances for later reference

### Methods

<static> fnReset(object)
:   Reset the column ordering for a DataTables instance

## Details

### Properties

<static> aoInstances :array
:   Array of all ColReorder instances for later reference

### Methods

<static> fnReset(object)
:   Reset the column ordering for a DataTables instance

    ##### Parameters:

    |  | Name | Type | Attributes | Default | Description |
    | --- | --- | --- | --- | --- | --- |
    | 1 | object |  |  |  | oTable DataTables instance to consider |

    ##### Returns:

    void

Documentation generated by JSDoc 3 on
22th Jun 2012 - 08:22
with the DataTables template.
