# Supplementary material for: Molecule database framework: a framework for creating database applications with chemical structure search capability
Source: J Cheminform. 2013 Dec 11;5:48. doi: 10.1186/1758-2946-5-48 (PMC3892073; doi:10.1186/1758-2946-5-48)
Supplement: Additional file 4 — MDF simple web application source code of the mercurial changeset 16f39f4e447b. [file 1758-2946-5-48-S4.zip › src/main/webapp/resources/js/datatables/ColReorder/media/docs/global.html]

Global - documentation


# Global

## Navigation

- Overview
- Summary

  Properties | Methods
- Details

  Properties | Methods

Hiding private elements
(toggle)

Showing extended elements
(toggle)

## Summary

### Properties

<constant> CLASS :String
:   Name of this class

<constant> VERSION :String
:   ColReorder version

### Methods

fnArraySwitch(array, int, int)
:   Modify an array by switching the position of two elements

fnDomSwitch(string, int, int)
:   Switch the positions of nodes in a parent node (note this is specifically designed for
    table rows). Note this function considers all element nodes under the parent!

fnInvertKeyValues(array)
:   Switch the key value pairing of an index array to be value key (i.e. the old value is now the
    key). For example consider [ 2, 0, 1 ] this would be returned as [ 1, 2, 0 ].

## Details

### Properties

<constant> CLASS :String
:   Name of this class

<constant> VERSION :String
:   ColReorder version

### Methods

fnArraySwitch(array, int, int)
:   Modify an array by switching the position of two elements

    ##### Parameters:

    |  | Name | Type | Attributes | Default | Description |
    | --- | --- | --- | --- | --- | --- |
    | 1 | array |  |  |  | aArray Array to consider, will be modified by reference (i.e. no return) |
    | 2 | int |  |  |  | iFrom From point |
    | 3 | int |  |  |  | iTo Insert point |

    ##### Returns:

    void

    fnDomSwitch(string, int, int)
    :   Switch the positions of nodes in a parent node (note this is specifically designed for
        table rows). Note this function considers all element nodes under the parent!

        ##### Parameters:

        |  | Name | Type | Attributes | Default | Description |
        | --- | --- | --- | --- | --- | --- |
        | 1 | string |  |  |  | sTag Tag to consider |
        | 2 | int |  |  |  | iFrom Element to move |
        | 3 | int |  |  |  | Point to element the element to (before this point), can be null for append |

        ##### Returns:

        void

        fnInvertKeyValues(array)
        :   Switch the key value pairing of an index array to be value key (i.e. the old value is now the
            key). For example consider [ 2, 0, 1 ] this would be returned as [ 1, 2, 0 ].

            ##### Parameters:

            |  | Name | Type | Attributes | Default | Description |
            | --- | --- | --- | --- | --- | --- |
            | 1 | array |  |  |  | aIn Array to switch around |

            ##### Returns:

            array

Documentation generated by JSDoc 3 on
22th Jun 2012 - 08:22
with the DataTables template.
