# Supplementary material for: Molecule database framework: a framework for creating database applications with chemical structure search capability
Source: J Cheminform. 2013 Dec 11;5:48. doi: 10.1186/1758-2946-5-48 (PMC3892073; doi:10.1186/1758-2946-5-48)
Supplement: Additional file 4 — MDF simple web application source code of the mercurial changeset 16f39f4e447b. [file 1758-2946-5-48-S4.zip › src/main/webapp/resources/js/datatables/ColReorder/server_side.html]

ColReorder example


ColReorder example with server-side processing

# Preamble

Server-side processing can be exceptionally useful in DataTables when dealing with
massive data sets, and ColReorder works with this as would be expected. There must be
special consideration for the column ordering on the server-side script since the
columns can be in an unexpected order. For this you can either choose to use the
*sName* parameter for each column and take this into account in the server-side
script (the parameter 'sColumns' is a comma separated string of these sName parameters).

Alternatively use the more flexible mDataProp
option for each column. This allows you to use JSON objects which DataTables, so order doesn't
matter like it would do in an array. Again the server-side script must take this into account
through the *mDataProp\_{i}* which is sent for each column (so the server knows which
column is to be sorted on).

# Live example

| Rendering engine | Browser | Platform(s) | Engine version | CSS grade |
| --- | --- | --- | --- | --- |
| Rendering engine | Browser | Platform(s) | Engine version | CSS grade |
| --- | --- | --- | --- | --- |

# Examples

- Basic initialisation
- Styling the insert cursor
- Individual column filtering
- Integration with DataTables' ColVis plug-in
- Integration with DataTables' FixedColumns plug-in
- Integration with DataTables' FixedHeader plug-in
- Using a predefined column order set
- Providing a user control to reset the column order
- Column reordering shown with scrolling in DataTables
- Server-side processing support
- State saving of the column position
- jQuery UI theme integration

# Initialisation code

```
$(document).ready( function () {
	var oTable = $('#example').dataTable( {
		"sDom": 'Rlfrtip',
		"bProcessing": true,
		"bServerSide": true,
		"sAjaxSource": "../../examples/server_side/scripts/objects.php",
		"aoColumns": [
			{ "mDataProp": "engine" },
			{ "mDataProp": "browser" },
			{ "mDataProp": "platform" },
			{ "mDataProp": "version" },
			{ "mDataProp": "grade" }
		]
	} );
} );
```

# Example JSON return from the server

```
{
"sEcho": 1,
"iTotalRecords": "57",
"iTotalDisplayRecords": "57",
"aaData": [
    {
        "engine": "Gecko",
        "browser": "Firefox 1.0",
        "platform": "Win 98+ / OSX.2+",
        "version": "1.7",
        "grade": "A"
    },
    {
        "engine": "Gecko",
        "browser": "Firefox 1.5",
        "platform": "Win 98+ / OSX.2+",
        "version": "1.8",
        "grade": "A"
    },
    ...
  ]
}
```

ColReorder and DataTables © Allan Jardine 2010
