# Supplementary material for: Molecule database framework: a framework for creating database applications with chemical structure search capability
Source: J Cheminform. 2013 Dec 11;5:48. doi: 10.1186/1758-2946-5-48 (PMC3892073; doi:10.1186/1758-2946-5-48)
Supplement: Additional file 4 — MDF simple web application source code of the mercurial changeset 16f39f4e447b. [file 1758-2946-5-48-S4.zip › src/main/webapp/resources/js/datatables/ColVis/media/docs/ccb5a49865.html]

Namespace: Settings object which contains customisable information for ColVis instance - documentation


# Namespace: Settings object which contains customisable information for ColVis instance

## Navigation

- Overview
- Summary
- Details

Hiding private elements
(toggle)

Showing extended elements
(toggle)

Documentation generated by JSDoc 3 on
22th Jun 2012 - 08:21
with the DataTables template.
