# Supplementary material for: Molecule database framework: a framework for creating database applications with chemical structure search capability
Source: J Cheminform. 2013 Dec 11;5:48. doi: 10.1186/1758-2946-5-48 (PMC3892073; doi:10.1186/1758-2946-5-48)
Supplement: Additional file 4 — MDF simple web application source code of the mercurial changeset 16f39f4e447b. [file 1758-2946-5-48-S4.zip › src/main/webapp/resources/js/datatables/ColVis/media/docs/global.html]

Global - documentation


# Global

## Navigation

- Overview
- Summary

  Properties | Methods
- Details

  Properties | Methods

Hiding private elements
(toggle)

Showing extended elements
(toggle)

## Summary

### Properties

<constant> CLASS :String
:   Name of this class

<constant> VERSION :String
:   ColVis version

### Methods

fnRebuild()
:   Rebuild the list of buttons for this instance (i.e. if there is a column header update)

## Details

### Properties

<constant> CLASS :String
:   Name of this class

<constant> VERSION :String
:   ColVis version

### Methods

fnRebuild()
:   Rebuild the list of buttons for this instance (i.e. if there is a column header update)

    ##### Returns:

    void

Documentation generated by JSDoc 3 on
22th Jun 2012 - 08:21
with the DataTables template.
