# Supplementary material for: Molecule database framework: a framework for creating database applications with chemical structure search capability
Source: J Cheminform. 2013 Dec 11;5:48. doi: 10.1186/1758-2946-5-48 (PMC3892073; doi:10.1186/1758-2946-5-48)
Supplement: Additional file 4 — MDF simple web application source code of the mercurial changeset 16f39f4e447b. [file 1758-2946-5-48-S4.zip › src/main/webapp/resources/js/datatables/ColVis/mouseover.html]

ColVis example


ColVis example - mouse over activation

# Preamble

The default activation (showing the columns list) for ColVis is for the user to click
the button. This can be altered to a 'mouseover' activation by making use of the
oColVis.activate DataTables initialisation value and setting it to "mouseover". This is
shown in the example below.

# Live example

| Rendering engine | Browser | Platform(s) | Engine version | CSS grade |
| --- | --- | --- | --- | --- |
| Rendering engine | Browser | Platform(s) | Engine version | CSS grade |
| --- | --- | --- | --- | --- |
| Trident | Internet Explorer 4.0 | Win 95+ (Entity: &) | 4 | X |
| Trident | Internet Explorer 5.0 | Win 95+ | 5 | C |
| Trident | Internet Explorer 5.5 | Win 95+ | 5.5 | A |
| Trident | Internet Explorer 6 | Win 98+ | 6 | A |
| Trident | Internet Explorer 7 | Win XP SP2+ | 7 | A |
| Trident | AOL browser (AOL desktop) | Win XP | 6 | A |
| Gecko (UTF-8: $¢€) | Firefox 1.0 | Win 98+ / OSX.2+ | 1.7 | A |
| Gecko | Firefox 1.5 | Win 98+ / OSX.2+ | 1.8 | A |
| Gecko | Firefox 2.0 | Win 98+ / OSX.2+ | 1.8 | A |
| Gecko | Firefox 3.0 | Win 2k+ / OSX.3+ | 1.9 | A |
| Gecko | Camino 1.0 | OSX.2+ | 1.8 | A |
| Gecko | Camino 1.5 | OSX.3+ | 1.8 | A |
| Gecko | Netscape 7.2 | Win 95+ / Mac OS 8.6-9.2 | 1.7 | A |
| Gecko | Netscape Browser 8 | Win 98SE+ | 1.7 | A |
| Gecko | Netscape Navigator 9 | Win 98+ / OSX.2+ | 1.8 | A |
| Gecko | Mozilla 1.0 | Win 95+ / OSX.1+ | 1 | A |
| Gecko | Mozilla 1.1 | Win 95+ / OSX.1+ | 1.1 | A |
| Gecko | Mozilla 1.2 | Win 95+ / OSX.1+ | 1.2 | A |
| Gecko | Mozilla 1.3 | Win 95+ / OSX.1+ | 1.3 | A |
| Gecko | Mozilla 1.4 | Win 95+ / OSX.1+ | 1.4 | A |
| Gecko | Mozilla 1.5 | Win 95+ / OSX.1+ | 1.5 | A |
| Gecko | Mozilla 1.6 | Win 95+ / OSX.1+ | 1.6 | A |
| Gecko | Mozilla 1.7 | Win 98+ / OSX.1+ | 1.7 | A |
| Gecko | Mozilla 1.8 | Win 98+ / OSX.1+ | 1.8 | A |
| Gecko | Seamonkey 1.1 | Win 98+ / OSX.2+ | 1.8 | A |
| Gecko | Epiphany 2.20 | Gnome | 1.8 | A |
| Webkit | Safari 1.2 | OSX.3 | 125.5 | A |
| Webkit | Safari 1.3 | OSX.3 | 312.8 | A |
| Webkit | Safari 2.0 | OSX.4+ | 419.3 | A |
| Webkit | Safari 3.0 | OSX.4+ | 522.1 | A |
| Webkit | OmniWeb 5.5 | OSX.4+ | 420 | A |
| Webkit | iPod Touch / iPhone | iPod | 420.1 | A |
| Webkit | S60 | S60 | 413 | A |
| Presto | Opera 7.0 | Win 95+ / OSX.1+ | - | A |
| Presto | Opera 7.5 | Win 95+ / OSX.2+ | - | A |
| Presto | Opera 8.0 | Win 95+ / OSX.2+ | - | A |
| Presto | Opera 8.5 | Win 95+ / OSX.2+ | - | A |
| Presto | Opera 9.0 | Win 95+ / OSX.3+ | - | A |
| Presto | Opera 9.2 | Win 88+ / OSX.3+ | - | A |
| Presto | Opera 9.5 | Win 88+ / OSX.3+ | - | A |
| Presto | Opera for Wii | Wii | - | A |
| Presto | Nokia N800 | N800 | - | A |
| Presto | Nintendo DS browser | Nintendo DS | 8.5 | C/A1 |
| KHTML | Konqureror 3.1 | KDE 3.1 | 3.1 | C |
| KHTML | Konqureror 3.3 | KDE 3.3 | 3.3 | A |
| KHTML | Konqureror 3.5 | KDE 3.5 | 3.5 | A |
| Tasman | Internet Explorer 4.5 | Mac OS 8-9 | - | X |
| Tasman | Internet Explorer 5.1 | Mac OS 7.6-9 | 1 | C |
| Tasman | Internet Explorer 5.2 | Mac OS 8-X | 1 | C |
| Misc | NetFront 3.1 | Embedded devices | - | C |
| Misc | NetFront 3.4 | Embedded devices | - | A |
| Misc | Dillo 0.8 | Embedded devices | - | X |
| Misc | Links | Text only | - | X |
| Misc | Lynx | Text only | - | X |
| Misc | IE Mobile | Windows Mobile 6 | - | C |
| Misc | PSP browser | PSP | - | C |
| Other browsers | All others | - | - | U |

# Examples

- Basic initialisation
- Activate with a mouseover rather than click
- Custom text in the button
- Using with jQuery UI ThemeRoller
- Excluding columns from the show / hide list
- Two tables initialised together - individual controls
- Two tables initialised together - shared control
- Alternative styling and reset to original
- Customisation of the button label with a callback

# Initialisation code

```
$(document).ready( function () {
	$('#example').dataTable( {
		"sDom": 'C<"clear">lfrtip',
		"oColVis": {
			"activate": "mouseover"
		}
	} );
} );
```

ColVis and DataTables © Allan Jardine 2009-2010
