# Supplementary material for: Molecule database framework: a framework for creating database applications with chemical structure search capability
Source: J Cheminform. 2013 Dec 11;5:48. doi: 10.1186/1758-2946-5-48 (PMC3892073; doi:10.1186/1758-2946-5-48)
Supplement: Additional file 4 — MDF simple web application source code of the mercurial changeset 16f39f4e447b. [file 1758-2946-5-48-S4.zip › src/main/webapp/resources/js/datatables/FixedColumns/docs/526f872207.html]

Namespace: grid - documentation


# Namespace: grid

## Ancestry: FixedColumns » #dom. » grid

FixedColumns v2.0.3 documentation

## Navigation

- Overview
- Summary

  Namespaces | Properties
- Details

  Properties

Hiding private elements
(toggle)

Showing extended elements
(toggle)

Display grid elements

## Summary

### Namespaces

left
:   Left fixed column grid components

right
:   Right fixed column grid components

### Properties

<static> dt :node
:   DataTables scrolling element. This element is the DataTables
    component in the display grid (making up the main table - i.e.
    not the fixed columns).

<static> wrapper :node
:   Grid wrapper. This is the container element for the 3x3 grid

## Details

### Properties

<static> dt :node
:   DataTables scrolling element. This element is the DataTables
    component in the display grid (making up the main table - i.e.
    not the fixed columns).

<static> wrapper :node
:   Grid wrapper. This is the container element for the 3x3 grid

FixedColumns: Copyright 2010-2011 Allan Jardine, all rights reserved  
Documentation generated by JSDoc 3 on
22th Jun 2012 - 08:21
with the DataTables template.
