# Supplementary material for: Molecule database framework: a framework for creating database applications with chemical structure search capability
Source: J Cheminform. 2013 Dec 11;5:48. doi: 10.1186/1758-2946-5-48 (PMC3892073; doi:10.1186/1758-2946-5-48)
Supplement: Additional file 4 — MDF simple web application source code of the mercurial changeset 16f39f4e447b. [file 1758-2946-5-48-S4.zip › src/main/webapp/resources/js/datatables/FixedColumns/docs/73098af57c.html]

Namespace: right - documentation


# Namespace: right

## Ancestry: FixedColumns » #dom » .grid. » right

FixedColumns v2.0.3 documentation

## Navigation

- Overview
- Summary
- Details

Hiding private elements
(toggle)

Showing extended elements
(toggle)

Right fixed column grid components

FixedColumns: Copyright 2010-2011 Allan Jardine, all rights reserved  
Documentation generated by JSDoc 3 on
22th Jun 2012 - 08:21
with the DataTables template.
