# Supplementary material for: Molecule database framework: a framework for creating database applications with chemical structure search capability
Source: J Cheminform. 2013 Dec 11;5:48. doi: 10.1186/1758-2946-5-48 (PMC3892073; doi:10.1186/1758-2946-5-48)
Supplement: Additional file 4 — MDF simple web application source code of the mercurial changeset 16f39f4e447b. [file 1758-2946-5-48-S4.zip › src/main/webapp/resources/js/datatables/FixedColumns/docs/91bce7c4ad.html]

Namespace: s - documentation


# Namespace: s

## Ancestry: FixedColumns# » s

FixedColumns v2.0.3 documentation

## Navigation

- Overview
- Summary

  Properties
- Details

  Properties

Hiding private elements
(toggle)

Showing extended elements
(toggle)

Settings object which contains customisable information for FixedColumns instance

### Extends

- FixedColumns.defaults

## Summary

### Properties

<static> aiWidths :array.<int>
:   Original widths of the columns as rendered by DataTables

<static, readonly> bOldIE :boolean
:   Flag to indicate if we are dealing with IE6/7 as these browsers need a little hack
    in the odd place

<static> dt :object
:   DataTables settings objects

<static> fnDrawCallback :function(object, object):void
:   Draw callback function which is called when FixedColumns has redrawn the fixed assets

<static> iLeftColumns :int
:   Number of left hand columns to fix in position

<static> iLeftWidth :int
:   Width to set for the width of the left fixed column(s) - note that the behaviour of this
    property is directly effected by the sLeftWidth property. If not defined then this property
    is calculated automatically from what has been assigned by DataTables.

<static> iRightColumns :int
:   Number of right hand columns to fix in position

<static> iRightWidth :int
:   Width to set for the width of the right fixed column(s) - note that the behaviour of this
    property is directly effected by the sRightWidth property. If not defined then this property
    is calculated automatically from what has been assigned by DataTables.

<static> iTableColumns :int
:   Number of columns in the DataTable - stored for quick access

<static> sHeightMatch :string
:   Height matching algorthim to use. This can be "none" which will result in no height
    matching being applied by FixedColumns (height matching could be forced by CSS in this
    case), "semiauto" whereby the height calculation will be performed once, and the result
    cached to be used again (fnRecalculateHeight can be used to force recalculation), or
    "auto" when height matching is performed on every draw (slowest but must accurate)

<static> sLeftWidth :string
:   Type of left column size calculation. Can take the values of "fixed", whereby the iLeftWidth
    value will be treated as a pixel value, or "relative" for which case iLeftWidth will be
    treated as a percentage value.

<static> sRightWidth :string
:   Type of right column size calculation. Can take the values of "fixed", whereby the
    iRightWidth value will be treated as a pixel value, or "relative" for which case
    iRightWidth will be treated as a percentage value.

## Details

### Properties

<static> aiWidths :array.<int>
:   Original widths of the columns as rendered by DataTables

<static, readonly> bOldIE :boolean
:   Flag to indicate if we are dealing with IE6/7 as these browsers need a little hack
    in the odd place

<static> dt :object
:   DataTables settings objects

<static> fnDrawCallback :function(object, object):void
:   Draw callback function which is called when FixedColumns has redrawn the fixed assets

    ##### Extended from:

    FixedColumns.defaults

    ##### Example

    ```
     	var oTable = $('#example').dataTable( {
     		"sScrollX": "100%"
     	} );
     	new FixedColumns( oTable, {
     		"fnDrawCallback": function () {
    				alert( "FixedColumns redraw" );
    			}
     	} );
    ```

<static> iLeftColumns :int
:   Number of left hand columns to fix in position

    ##### Extended from:

    FixedColumns.defaults

    ##### Example

    ```
     	var oTable = $('#example').dataTable( {
     		"sScrollX": "100%"
     	} );
     	new FixedColumns( oTable, {
     		"iLeftColumns": 2
     	} );
    ```

<static> iLeftWidth :int
:   Width to set for the width of the left fixed column(s) - note that the behaviour of this
    property is directly effected by the sLeftWidth property. If not defined then this property
    is calculated automatically from what has been assigned by DataTables.

    ##### Extended from:

    FixedColumns.defaults

    ##### Example

    ```
     	var oTable = $('#example').dataTable( {
     		"sScrollX": "100%"
     	} );
     	new FixedColumns( oTable, {
     		"iLeftWidth": 100 // pixels
     	} );
    ```

<static> iRightColumns :int
:   Number of right hand columns to fix in position

    ##### Extended from:

    FixedColumns.defaults

    ##### Example

    ```
     	var oTable = $('#example').dataTable( {
     		"sScrollX": "100%"
     	} );
     	new FixedColumns( oTable, {
     		"iRightColumns": 1
     	} );
    ```

<static> iRightWidth :int
:   Width to set for the width of the right fixed column(s) - note that the behaviour of this
    property is directly effected by the sRightWidth property. If not defined then this property
    is calculated automatically from what has been assigned by DataTables.

    ##### Extended from:

    FixedColumns.defaults

    ##### Example

    ```
     	var oTable = $('#example').dataTable( {
     		"sScrollX": "100%"
     	} );
     	new FixedColumns( oTable, {
     		"iRightWidth": 200 // pixels
     	} );
    ```

<static> iTableColumns :int
:   Number of columns in the DataTable - stored for quick access

<static> sHeightMatch :string
:   Height matching algorthim to use. This can be "none" which will result in no height
    matching being applied by FixedColumns (height matching could be forced by CSS in this
    case), "semiauto" whereby the height calculation will be performed once, and the result
    cached to be used again (fnRecalculateHeight can be used to force recalculation), or
    "auto" when height matching is performed on every draw (slowest but must accurate)

    ##### Extended from:

    FixedColumns.defaults

    ##### Example

    ```
     	var oTable = $('#example').dataTable( {
     		"sScrollX": "100%"
     	} );
     	new FixedColumns( oTable, {
     		"sHeightMatch": "auto"
     	} );
    ```

<static> sLeftWidth :string
:   Type of left column size calculation. Can take the values of "fixed", whereby the iLeftWidth
    value will be treated as a pixel value, or "relative" for which case iLeftWidth will be
    treated as a percentage value.

    ##### Extended from:

    FixedColumns.defaults

    ##### Example

    ```
     	var oTable = $('#example').dataTable( {
     		"sScrollX": "100%"
     	} );
     	new FixedColumns( oTable, {
     		"sLeftWidth": "relative",
     		"iLeftWidth": 10 // percentage
     	} );
    ```

<static> sRightWidth :string
:   Type of right column size calculation. Can take the values of "fixed", whereby the
    iRightWidth value will be treated as a pixel value, or "relative" for which case
    iRightWidth will be treated as a percentage value.

    ##### Extended from:

    FixedColumns.defaults

    ##### Example

    ```
     	var oTable = $('#example').dataTable( {
     		"sScrollX": "100%"
     	} );
     	new FixedColumns( oTable, {
     		"sRightWidth": "relative",
     		"iRightWidth": 10 // percentage
     	} );
    ```

FixedColumns: Copyright 2010-2011 Allan Jardine, all rights reserved  
Documentation generated by JSDoc 3 on
22th Jun 2012 - 08:21
with the DataTables template.
