# Supplementary material for: Molecule database framework: a framework for creating database applications with chemical structure search capability
Source: J Cheminform. 2013 Dec 11;5:48. doi: 10.1186/1758-2946-5-48 (PMC3892073; doi:10.1186/1758-2946-5-48)
Supplement: Additional file 4 — MDF simple web application source code of the mercurial changeset 16f39f4e447b. [file 1758-2946-5-48-S4.zip › src/main/webapp/resources/js/datatables/FixedColumns/docs/d3890ba7c4.html]

Namespace: dom - documentation


# Namespace: dom

## Ancestry: FixedColumns# » dom

FixedColumns v2.0.3 documentation

## Navigation

- Overview
- Summary

  Namespaces | Properties
- Details

  Properties

Hiding private elements
(toggle)

Showing extended elements
(toggle)

DOM elements used by the class instance

## Summary

### Namespaces

clone
:   Cloned table nodes

grid
:   Display grid elements

### Properties

<static> body :node
:   DataTables body table

<static> footer :node
:   DataTables footer table

<static> header :node
:   DataTables header table

<static> scroller :node
:   DataTables scrolling element

## Details

### Properties

<static> body :node
:   DataTables body table

<static> footer :node
:   DataTables footer table

<static> header :node
:   DataTables header table

<static> scroller :node
:   DataTables scrolling element

FixedColumns: Copyright 2010-2011 Allan Jardine, all rights reserved  
Documentation generated by JSDoc 3 on
22th Jun 2012 - 08:21
with the DataTables template.
