# Supplementary material for: Molecule database framework: a framework for creating database applications with chemical structure search capability
Source: J Cheminform. 2013 Dec 11;5:48. doi: 10.1186/1758-2946-5-48 (PMC3892073; doi:10.1186/1758-2946-5-48)
Supplement: Additional file 4 — MDF simple web application source code of the mercurial changeset 16f39f4e447b. [file 1758-2946-5-48-S4.zip › src/main/webapp/resources/js/datatables/FixedColumns/docs/FixedColumns.html]

Class: FixedColumns - documentation


# Class: FixedColumns

FixedColumns v2.0.3 documentation

## Navigation

- Overview
- Summary

  Namespaces | Properties | Methods | Events
- Details

  Properties | Methods | Events

Hiding private elements
(toggle)

Showing extended elements
(toggle)

new FixedColumns(oDT, oInit)
:   When making use of DataTables' x-axis scrolling feature, you may wish to
    fix the left most column in place. This plug-in for DataTables provides
    exactly this option (note for non-scrolling tables, please use the
    FixedHeader plug-in, which can fix headers, footers and columns). Key
    features include:

    - Freezes the left or right most columns to the side of the table
    - Option to freeze two or more columns
    - Full integration with DataTables' scrolling options
    - Speed - FixedColumns is fast in its operation

    ### Constructor

    ##### Parameters:

    |  | Name | Type | Attributes | Default | Description |
    | --- | --- | --- | --- | --- | --- |
    | 1 | oDT | object |  |  | DataTables instance |
    | 2 | oInit | object | Optional | {} | Configuration object for FixedColumns. Options are defined by FixedColumns.defaults |

    ##### Example:

    ```
     	var oTable = $('#example').dataTable( {
     		"sScrollX": "100%"
     	} );
     	new FixedColumns( oTable );
    ```

### Requires

- module:jQuery
- module:DataTables

## Summary

### Namespaces

defaults
:   FixedColumns default settings for initialisation

dom
:   DOM elements used by the class instance

s
:   Settings object which contains customisable information for FixedColumns instance

### Properties

<static, constant> VERSION :String
:   FixedColumns version

### Methods

fnRecalculateHeight(nTr) → {void}
:   Mark a row such that it's height should be recalculated when using 'semiauto' row
    height matching. This function will have no effect when 'none' or 'auto' row height
    matching is used.

fnRedrawLayout() → {void}
:   Recalculate the resizes of the 3x3 grid that FixedColumns uses for display of the table.
    This is useful if you update the width of the table container. Note that FixedColumns will
    perform this function automatically when the window.resize event is fired.

fnSetRowHeight(nTarget, iHeight) → {void}
:   Set the height of a given row - provides cross browser compatibility

fnUpdate() → {void}
:   Update the fixed columns - including headers and footers. Note that FixedColumns will
    automatically update the display whenever the host DataTable redraws.

### Events

draw
:   Event fired whenever FixedColumns redraws the fixed columns (i.e. clones the table elements from the main DataTable). This will occur whenever the DataTable that the FixedColumns instance is attached does its own draw.

## Details

### Properties

<static, constant> VERSION :String
:   FixedColumns version

### Methods

fnRecalculateHeight(nTr) → {void}
:   Mark a row such that it's height should be recalculated when using 'semiauto' row
    height matching. This function will have no effect when 'none' or 'auto' row height
    matching is used.

    ##### Parameters:

    |  | Name | Type | Attributes | Default | Description |
    | --- | --- | --- | --- | --- | --- |
    | 1 | nTr | Node |  |  | TR element that should have it's height recalculated |

    ##### Example:

    ```
     	var oTable = $('#example').dataTable( {
     		"sScrollX": "100%"
     	} );
     	var oFC = new FixedColumns( oTable );
     	
     	// manipulate the table - mark the row as needing an update then update the table
     	// this allows the redraw performed by DataTables fnUpdate to recalculate the row
     	// height
     	oFC.fnRecalculateHeight();
     	oTable.fnUpdate( $('#example tbody tr:eq(0)')[0], ["insert date", 1, 2, 3 ... ]);
    ```

    fnRedrawLayout() → {void}
    :   Recalculate the resizes of the 3x3 grid that FixedColumns uses for display of the table.
        This is useful if you update the width of the table container. Note that FixedColumns will
        perform this function automatically when the window.resize event is fired.

        ##### Example:

        ```
         	var oTable = $('#example').dataTable( {
         		"sScrollX": "100%"
         	} );
         	var oFC = new FixedColumns( oTable );
         	
         	// Resize the table container and then have FixedColumns adjust its layout....
             $('#content').width( 1200 );
         	oFC.fnRedrawLayout();
        ```

        fnSetRowHeight(nTarget, iHeight) → {void}
        :   Set the height of a given row - provides cross browser compatibility

            ##### Parameters:

            |  | Name | Type | Attributes | Default | Description |
            | --- | --- | --- | --- | --- | --- |
            | 1 | nTarget | Node |  |  | TR element that should have it's height recalculated |
            | 2 | iHeight | int |  |  | Height in pixels to set |

            ##### Example:

            ```
             	var oTable = $('#example').dataTable( {
             		"sScrollX": "100%"
             	} );
             	var oFC = new FixedColumns( oTable );
             	
             	// You may want to do this after manipulating a row in the fixed column
             	oFC.fnSetRowHeight( $('#example tbody tr:eq(0)')[0], 50 );
            ```

            fnUpdate() → {void}
            :   Update the fixed columns - including headers and footers. Note that FixedColumns will
                automatically update the display whenever the host DataTable redraws.

                ##### Example:

                ```
                 	var oTable = $('#example').dataTable( {
                 		"sScrollX": "100%"
                 	} );
                 	var oFC = new FixedColumns( oTable );
                 	
                 	// at some later point when the table has been manipulated....
                 	oFC.fnUpdate();
                ```

### Events

draw
:   Event fired whenever FixedColumns redraws the fixed columns (i.e. clones the table elements from the main DataTable). This will occur whenever the DataTable that the FixedColumns instance is attached does its own draw.

    ##### Parameters:

    |  | Name | Type | Attributes | Default | Description |
    | --- | --- | --- | --- | --- | --- |
    | 1 | e | event |  |  | jQuery event object |
    | 2 | o | object |  |  | Event parameters from FixedColumns |
    |  | o.leftClone | object |  |  | Instance's object dom.clone.left for easy reference. This object contains references to the left fixed clumn column's nodes |
    |  | o.rightClone | object |  |  | Instance's object dom.clone.right for easy reference. This object contains references to the right fixed clumn column's nodes |

FixedColumns: Copyright 2010-2011 Allan Jardine, all rights reserved  
Documentation generated by JSDoc 3 on
22th Jun 2012 - 08:21
with the DataTables template.
