# Supplementary material for: Molecule database framework: a framework for creating database applications with chemical structure search capability
Source: J Cheminform. 2013 Dec 11;5:48. doi: 10.1186/1758-2946-5-48 (PMC3892073; doi:10.1186/1758-2946-5-48)
Supplement: Additional file 4 — MDF simple web application source code of the mercurial changeset 16f39f4e447b. [file 1758-2946-5-48-S4.zip › src/main/webapp/resources/js/datatables/FixedColumns/docs/index.html]

Table of Contents - documentation


### Table of Contents

FixedColumns
:   When making use of DataTables' x-axis scrolling feature, you may wish to
    fix the left most column in place. This plug-in for DataTables provides
    exactly this option (note for non-scrolling tables, please use the
    FixedHeader plug-in, which can fix headers, footers and columns). Key
    features include:

    - Freezes the left or right most columns to the side of the table
    - Option to freeze two or more columns
    - Full integration with DataTables' scrolling options
    - Speed - FixedColumns is fast in its operation

FixedColumns: Copyright 2010-2011 Allan Jardine, all rights reserved  
Documentation generated by JSDoc 3 on
22th Jun 2012 - 08:21
with the DataTables template.
