# Supplementary material for: Molecule database framework: a framework for creating database applications with chemical structure search capability
Source: J Cheminform. 2013 Dec 11;5:48. doi: 10.1186/1758-2946-5-48 (PMC3892073; doi:10.1186/1758-2946-5-48)
Supplement: Additional file 4 — MDF simple web application source code of the mercurial changeset 16f39f4e447b. [file 1758-2946-5-48-S4.zip › src/main/webapp/resources/js/datatables/FixedColumns/index_column.html]

FixedColumns example


FixedColumns example - index column

# Preamble

A typical interaction to want to perform with a fixed column, is an index column. A method for how this can be achieved with FixedColumns is shown in this example, building on the index column example for DataTables. Also shown in this example is how the fixed column can be styled with CSS to show it more prominently.

# Live example

|  | Rendering engine | Browser | Platform(s) | Engine version | CSS grade |
| --- | --- | --- | --- | --- | --- |
| 1 | Trident | Internet Explorer 4.0 | Win 95+ | 4 | X |
| 2 | Trident | Internet Explorer 5.0 | Win 95+ | 5 | C |
| 3 | Trident | Internet Explorer 5.5 | Win 95+ | 5.5 | A |
| 4 | Trident | Internet Explorer 6 | Win 98+ | 6 | A |
| 5 | Trident | Internet Explorer 7 | Win XP SP2+ | 7 | A |
| 6 | Trident | AOL browser (AOL desktop) | Win XP | 6 | A |
| 7 | Gecko | Firefox 1.0 | Win 98+ / OSX.2+ | 1.7 | A |
| 8 | Gecko | Firefox 1.5 | Win 98+ / OSX.2+ | 1.8 | A |
| 9 | Gecko | Firefox 2.0 | Win 98+ / OSX.2+ | 1.8 | A |
| 10 | Gecko | Firefox 3.0 | Win 2k+ / OSX.3+ | 1.9 | A |
| 11 | Gecko | Camino 1.0 | OSX.2+ | 1.8 | A |
| 12 | Gecko | Camino 1.5 | OSX.3+ | 1.8 | A |
| 13 | Gecko | Netscape 7.2 | Win 95+ / Mac OS 8.6-9.2 | 1.7 | A |
| 14 | Gecko | Netscape Browser 8 | Win 98SE+ | 1.7 | A |
| 15 | Gecko | Netscape Navigator 9 | Win 98+ / OSX.2+ | 1.8 | A |
| 16 | Gecko | Mozilla 1.0 | Win 95+ / OSX.1+ | 1 | A |
| 17 | Gecko | Mozilla 1.1 | Win 95+ / OSX.1+ | 1.1 | A |
| 18 | Gecko | Mozilla 1.2 | Win 95+ / OSX.1+ | 1.2 | A |
| 19 | Gecko | Mozilla 1.3 | Win 95+ / OSX.1+ | 1.3 | A |
| 20 | Gecko | Mozilla 1.4 | Win 95+ / OSX.1+ | 1.4 | A |
| 21 | Gecko | Mozilla 1.5 | Win 95+ / OSX.1+ | 1.5 | A |
| 22 | Gecko | Mozilla 1.6 | Win 95+ / OSX.1+ | 1.6 | A |
| 23 | Gecko | Mozilla 1.7 | Win 98+ / OSX.1+ | 1.7 | A |
| 24 | Gecko | Mozilla 1.8 | Win 98+ / OSX.1+ | 1.8 | A |
| 25 | Gecko | Seamonkey 1.1 | Win 98+ / OSX.2+ | 1.8 | A |
| 26 | Gecko | Epiphany 2.20 | Gnome | 1.8 | A |
| 27 | Webkit | Safari 1.2 | OSX.3 | 125.5 | A |
| 28 | Webkit | Safari 1.3 | OSX.3 | 312.8 | A |
| 29 | Webkit | Safari 2.0 | OSX.4+ | 419.3 | A |
| 30 | Webkit | Safari 3.0 | OSX.4+ | 522.1 | A |
| 31 | Webkit | OmniWeb 5.5 | OSX.4+ | 420 | A |
| 32 | Webkit | iPod Touch / iPhone | iPod | 420.1 | A |
| 33 | Webkit | S60 | S60 | 413 | A |
| 34 | Presto | Opera 7.0 | Win 95+ / OSX.1+ | - | A |
| 35 | Presto | Opera 7.5 | Win 95+ / OSX.2+ | - | A |
| 36 | Presto | Opera 8.0 | Win 95+ / OSX.2+ | - | A |
| 37 | Presto | Opera 8.5 | Win 95+ / OSX.2+ | - | A |
| 38 | Presto | Opera 9.0 | Win 95+ / OSX.3+ | - | A |
| 39 | Presto | Opera 9.2 | Win 88+ / OSX.3+ | - | A |
| 40 | Presto | Opera 9.5 | Win 88+ / OSX.3+ | - | A |
| 41 | Presto | Opera for Wii | Wii | - | A |
| 42 | Presto | Nokia N800 | N800 | - | A |
| 43 | Presto | Nintendo DS browser | Nintendo DS | 8.5 | C/A |
| 44 | KHTML | Konqureror 3.1 | KDE 3.1 | 3.1 | C |
| 45 | KHTML | Konqureror 3.3 | KDE 3.3 | 3.3 | A |
| 46 | KHTML | Konqureror 3.5 | KDE 3.5 | 3.5 | A |
| 47 | Tasman | Internet Explorer 4.5 | Mac OS 8-9 | - | X |
| 48 | Tasman | Internet Explorer 5.1 | Mac OS 7.6-9 | 1 | C |
| 49 | Tasman | Internet Explorer 5.2 | Mac OS 8-X | 1 | C |
| 50 | Misc | NetFront 3.1 | Embedded devices | - | C |
| 51 | Misc | NetFront 3.4 | Embedded devices | - | A |
| 52 | Misc | Dillo 0.8 | Embedded devices | - | X |
| 53 | Misc | Links | Text only | - | X |
| 54 | Misc | Lynx | Text only | - | X |
| 55 | Misc | IE Mobile | Windows Mobile 6 | - | C |
| 56 | Misc | PSP browser | PSP | - | C |
| 57 | Other browsers | All others | - | - | U |
|  | Rendering engine | Browser | Platform(s) | Engine version | CSS grade |
| --- | --- | --- | --- | --- | --- |

# Initialisation code

```
$(document).ready( function () {
	var oTable = $('#example').dataTable( {
		"sScrollX": "100%",
		"sScrollXInner": "150%",
		"bScrollCollapse": true,
		"fnDrawCallback": function ( oSettings ) {
			/* Need to redo the counters if filtered or sorted */
			if ( oSettings.bSorted || oSettings.bFiltered ) {
				for ( var i=0, iLen=oSettings.aiDisplay.length ; i<iLen ; i++ ) {
					this.fnUpdate( i+1, oSettings.aiDisplay[i], 0, false, false );
				}
			}
		},
		"aoColumnDefs": [
			{ "bSortable": false, "sClass": "index", "aTargets": [ 0 ] }
		],
		"aaSorting": [[ 1, 'asc' ]]
	} );
	new FixedColumns( oTable );
} );
```

# Documentation

- Usage
- API / parameters

# Basic examples

- Basic demo of FixedColumns with zero initialisation
- Fix the left and right columns in place
- Index column attached to side of the table
- Index column, but with Y scrolling and no pagination
- Two columns fixed in place
- Fix the right column in place
- Speeding up row height matching with CSS
- jQuery UI themed table with FixedColumns

# Advanced examples

- Using ROWSPAN with FixedColumns
- Server-side processing with FixedColumns
- Individual column filtering
- Setting the fixed column width and resizing the table
- Setting the fixed column width with relative sizing
- Row grouping by grouping like rows in the fixed column
- Row grouping by inserting a grouping row

FixedColumns and DataTables © Allan Jardine 2011.
