# Supplementary material for: Molecule database framework: a framework for creating database applications with chemical structure search capability
Source: J Cheminform. 2013 Dec 11;5:48. doi: 10.1186/1758-2946-5-48 (PMC3892073; doi:10.1186/1758-2946-5-48)
Supplement: Additional file 4 — MDF simple web application source code of the mercurial changeset 16f39f4e447b. [file 1758-2946-5-48-S4.zip › src/main/webapp/resources/js/datatables/FixedColumns/row_grouping.html]

FixedColumns example


FixedColumns example - row grouping

# Preamble

This example is designed to re-implement the visual effect of the DataTables row grouping example with a fixed column. It is alternative row grouping style to that presented in the row grouping by height example.

# Live example

| Browser | Rendering engine | Platform(s) | Engine version | CSS grade |
| --- | --- | --- | --- | --- |
| Internet Explorer 4.0 | Trident | Win 95+ | 4 | X |
| Internet Explorer 5.0 | Trident | Win 95+ | 5 | C |
| Internet Explorer 5.5 | Trident | Win 95+ | 5.5 | A |
| Internet Explorer 6 | Trident | Win 98+ | 6 | A |
| Internet Explorer 7 | Trident | Win XP SP2+ | 7 | A |
| AOL browser (AOL desktop) | Trident | Win XP | 6 | A |
| Firefox 1.0 | Gecko | Win 98+ / OSX.2+ | 1.7 | A |
| Firefox 1.5 | Gecko | Win 98+ / OSX.2+ | 1.8 | A |
| Firefox 2.0 | Gecko | Win 98+ / OSX.2+ | 1.8 | A |
| Firefox 3.0 | Gecko | Win 2k+ / OSX.3+ | 1.9 | A |
| Camino 1.0 | Gecko | OSX.2+ | 1.8 | A |
| Camino 1.5 | Gecko | OSX.3+ | 1.8 | A |
| Netscape 7.2 | Gecko | Win 95+ / Mac OS 8.6-9.2 | 1.7 | A |
| Netscape Browser 8 | Gecko | Win 98SE+ | 1.7 | A |
| Netscape Navigator 9 | Gecko | Win 98+ / OSX.2+ | 1.8 | A |
| Mozilla 1.0 | Gecko | Win 95+ / OSX.1+ | 1 | A |
| Mozilla 1.1 | Gecko | Win 95+ / OSX.1+ | 1.1 | A |
| Mozilla 1.2 | Gecko | Win 95+ / OSX.1+ | 1.2 | A |
| Mozilla 1.3 | Gecko | Win 95+ / OSX.1+ | 1.3 | A |
| Mozilla 1.4 | Gecko | Win 95+ / OSX.1+ | 1.4 | A |
| Mozilla 1.5 | Gecko | Win 95+ / OSX.1+ | 1.5 | A |
| Mozilla 1.6 | Gecko | Win 95+ / OSX.1+ | 1.6 | A |
| Mozilla 1.7 | Gecko | Win 98+ / OSX.1+ | 1.7 | A |
| Mozilla 1.8 | Gecko | Win 98+ / OSX.1+ | 1.8 | A |
| Seamonkey 1.1 | Gecko | Win 98+ / OSX.2+ | 1.8 | A |
| Epiphany 2.20 | Gecko | Gnome | 1.8 | A |
| Safari 1.2 | Webkit | OSX.3 | 125.5 | A |
| Safari 1.3 | Webkit | OSX.3 | 312.8 | A |
| Safari 2.0 | Webkit | OSX.4+ | 419.3 | A |
| Safari 3.0 | Webkit | OSX.4+ | 522.1 | A |
| OmniWeb 5.5 | Webkit | OSX.4+ | 420 | A |
| iPod Touch / iPhone | Webkit | iPod | 420.1 | A |
| S60 | Webkit | S60 | 413 | A |
| Opera 7.0 | Presto | Win 95+ / OSX.1+ | - | A |
| Opera 7.5 | Presto | Win 95+ / OSX.2+ | - | A |
| Opera 8.0 | Presto | Win 95+ / OSX.2+ | - | A |
| Opera 8.5 | Presto | Win 95+ / OSX.2+ | - | A |
| Opera 9.0 | Presto | Win 95+ / OSX.3+ | - | A |
| Opera 9.2 | Presto | Win 88+ / OSX.3+ | - | A |
| Opera 9.5 | Presto | Win 88+ / OSX.3+ | - | A |
| Opera for Wii | Presto | Wii | - | A |
| Nokia N800 | Presto | N800 | - | A |
| Nintendo DS browser | Presto | Nintendo DS | 8.5 | C/A |
| Konqureror 3.1 | KHTML | KDE 3.1 | 3.1 | C |
| Konqureror 3.3 | KHTML | KDE 3.3 | 3.3 | A |
| Konqureror 3.5 | KHTML | KDE 3.5 | 3.5 | A |
| Internet Explorer 4.5 | Tasman | Mac OS 8-9 | - | X |
| Internet Explorer 5.1 | Tasman | Mac OS 7.6-9 | 1 | C |
| Internet Explorer 5.2 | Tasman | Mac OS 8-X | 1 | C |
| NetFront 3.1 | Misc | Embedded devices | - | C |
| NetFront 3.4 | Misc | Embedded devices | - | A |
| Dillo 0.8 | Misc | Embedded devices | - | X |
| Links | Misc | Text only | - | X |
| Lynx | Misc | Text only | - | X |
| IE Mobile | Misc | Windows Mobile 6 | - | C |
| PSP browser | Misc | PSP | - | C |
| All others | Other browsers | - | - | U |

# Initialisation code

```
$(document).ready( function () {
	var oTable = $('#example').dataTable( {
		"sScrollY": "300px",
		"sScrollX": "100%",
		"sScrollXInner": "150%",
		"bScrollCollapse": true,
		"bPaginate": false,
		"aaSortingFixed": [ [1, 'asc'] ],
		"aoColumnDefs": [
			{ "bVisible": false, "aTargets": [1] }
		]
	} );

	new FixedColumns( oTable, {
		"fnDrawCallback": function ( left, right ) {
			var oSettings = oTable.fnSettings();
			if ( oSettings.aiDisplay.length == 0 )
			{
				return;
			}

			var nGroup, nCell, iIndex, sGroup;
			var sLastGroup = "", iCorrector=0;
			var nTrs = $('#example tbody tr');
			var iColspan = nTrs[0].getElementsByTagName('td').length;

			for ( var i=0 ; i<nTrs.length ; i++ )
			{
				iIndex = oSettings._iDisplayStart + i;
				sGroup = oSettings.aoData[ oSettings.aiDisplay[iIndex] ]._aData[1]			;
				
				if ( sGroup != sLastGroup )
				{
					/* Cell to insert into main table */
					nGroup = document.createElement( 'tr' );
					nCell = document.createElement( 'td' );
					nCell.colSpan = iColspan;
					nCell.className = "group";
					nCell.innerHTML = "&nbsp;";
					nGroup.appendChild( nCell );
					nTrs[i].parentNode.insertBefore( nGroup, nTrs[i] );

					/* Cell to insert into the frozen columns */
					nGroup = document.createElement( 'tr' );
					nCell = document.createElement( 'td' );
					nCell.className = "group";
					nCell.innerHTML = sGroup;
					nGroup.appendChild( nCell );
					$(nGroup).insertBefore( $('tbody tr:eq('+(i+iCorrector)+')', left.body)[0] );

					iCorrector++;
					sLastGroup = sGroup;
				}
			}
		}
	} );
} );
```

# Documentation

- Usage
- API / parameters

# Basic examples

- Basic demo of FixedColumns with zero initialisation
- Fix the left and right columns in place
- Index column attached to side of the table
- Index column, but with Y scrolling and no pagination
- Two columns fixed in place
- Fix the right column in place
- Speeding up row height matching with CSS
- jQuery UI themed table with FixedColumns

# Advanced examples

- Using ROWSPAN with FixedColumns
- Server-side processing with FixedColumns
- Individual column filtering
- Setting the fixed column width and resizing the table
- Setting the fixed column width with relative sizing
- Row grouping by grouping like rows in the fixed column
- Row grouping by inserting a grouping row

FixedColumns and DataTables © Allan Jardine 2011.
