# Supplementary material for: Molecule database framework: a framework for creating database applications with chemical structure search capability
Source: J Cheminform. 2013 Dec 11;5:48. doi: 10.1186/1758-2946-5-48 (PMC3892073; doi:10.1186/1758-2946-5-48)
Supplement: Additional file 4 — MDF simple web application source code of the mercurial changeset 16f39f4e447b. [file 1758-2946-5-48-S4.zip › src/main/webapp/resources/js/datatables/FixedColumns/row_grouping_height.html]

FixedColumns example


FixedColumns example - row grouping using the fixed column

# Preamble

This example shows how it is possible to manipulate the DOM of the fixed column to create the effect of row grouping in the table (or more generally any manipulation you wish to make). When ordered by the first column it is possible to see the grouping in full effect - if you then change the ordering to one of the other columns the grouping is dynamically changed to combine like cells. It would be readily possible to extend this example to use two fixed columns and only 'group' rows in one of the columns, potentially achieving quite a powerful effect.

# Live example

| Rendering engine | Browser | Platform(s) | Engine version | CSS grade |
| --- | --- | --- | --- | --- |
| Trident | Internet Explorer 4.0 | Win 95+ | 4 | X |
| Trident | Internet Explorer 5.0 | Win 95+ | 5 | C |
| Trident | Internet Explorer 5.5 | Win 95+ | 5.5 | A |
| Trident | Internet Explorer 6 | Win 98+ | 6 | A |
| Trident | Internet Explorer 7 | Win XP SP2+ | 7 | A |
| Trident | AOL browser (AOL desktop) | Win XP | 6 | A |
| Gecko | Firefox 1.0 | Win 98+ / OSX.2+ | 1.7 | A |
| Gecko | Firefox  1.5 | Win 98+ / OSX.2+ | 1.8 | A |
| Gecko | Firefox 2.0 | Win 98+ / OSX.2+ | 1.8 | A |
| Gecko | Firefox 3.0 | Win 2k+ / OSX.3+ | 1.9 | A |
| Gecko | Camino 1.0 | OSX.2+ | 1.8 | A |
| Gecko | Camino 1.5 | OSX.3+ | 1.8 | A |
| Gecko | Netscape 7.2 | Win 95+ / Mac OS 8.6-9.2 | 1.7 | A |
| Gecko | Netscape Browser 8 | Win 98SE+ | 1.7 | A |
| Gecko | Netscape Navigator 9 | Win 98+ / OSX.2+ | 1.8 | A |
| Gecko | Mozilla 1.0 | Win 95+ / OSX.1+ | 1 | A |
| Gecko | Mozilla 1.1 | Win 95+ / OSX.1+ | 1.1 | A |
| Gecko | Mozilla 1.2 | Win 95+ / OSX.1+ | 1.2 | A |
| Gecko | Mozilla 1.3 | Win 95+ / OSX.1+ | 1.3 | A |
| Gecko | Mozilla 1.4 | Win 95+ / OSX.1+ | 1.4 | A |
| Gecko | Mozilla 1.5 | Win 95+ / OSX.1+ | 1.5 | A |
| Gecko | Mozilla 1.6 | Win 95+ / OSX.1+ | 1.6 | A |
| Gecko | Mozilla 1.7 | Win 98+ / OSX.1+ | 1.7 | A |
| Gecko | Mozilla 1.8 | Win 98+ / OSX.1+ | 1.8 | A |
| Gecko | Seamonkey 1.1 | Win 98+ / OSX.2+ | 1.8 | A |
| Gecko | Epiphany 2.20 | Gnome | 1.8 | A |
| Webkit | Safari 1.2 | OSX.3 | 125.5 | A |
| Webkit | Safari 1.3 | OSX.3 | 312.8 | A |
| Webkit | Safari 2.0 | OSX.4+ | 419.3 | A |
| Webkit | Safari 3.0 | OSX.4+ | 522.1 | A |
| Webkit | OmniWeb 5.5 | OSX.4+ | 420 | A |
| Webkit | iPod Touch / iPhone | iPod | 420.1 | A |
| Webkit | S60 | S60 | 413 | A |
| Presto | Opera 7.0 | Win 95+ / OSX.1+ | - | A |
| Presto | Opera 7.5 | Win 95+ / OSX.2+ | - | A |
| Presto | Opera 8.0 | Win 95+ / OSX.2+ | - | A |
| Presto | Opera 8.5 | Win 95+ / OSX.2+ | - | A |
| Presto | Opera 9.0 | Win 95+ / OSX.3+ | - | A |
| Presto | Opera 9.2 | Win 88+ / OSX.3+ | - | A |
| Presto | Opera 9.5 | Win 88+ / OSX.3+ | - | A |
| Presto | Opera for Wii | Wii | - | A |
| Presto | Nokia N800 | N800 | - | A |
| Presto | Nintendo DS browser | Nintendo DS | 8.5 | C/A |
| KHTML | Konqureror 3.1 | KDE 3.1 | 3.1 | C |
| KHTML | Konqureror 3.3 | KDE 3.3 | 3.3 | A |
| KHTML | Konqureror 3.5 | KDE 3.5 | 3.5 | A |
| Tasman | Internet Explorer 4.5 | Mac OS 8-9 | - | X |
| Tasman | Internet Explorer 5.1 | Mac OS 7.6-9 | 1 | C |
| Tasman | Internet Explorer 5.2 | Mac OS 8-X | 1 | C |
| Misc | NetFront 3.1 | Embedded devices | - | C |
| Misc | NetFront 3.4 | Embedded devices | - | A |
| Misc | Dillo 0.8 | Embedded devices | - | X |
| Misc | Links | Text only | - | X |
| Misc | Lynx | Text only | - | X |
| Misc | IE Mobile | Windows Mobile 6 | - | C |
| Misc | PSP browser | PSP | - | C |
| Other browsers | All others | - | - | U |

# Initialisation code

```
$(document).ready( function () {
	var oTable = $('#example').dataTable( {
		"sScrollY": "300px",
		"sScrollX": "100%",
		"sScrollXInner": "150%",
		"bScrollCollapse": true,
		"bPaginate": false
	} );

	new FixedColumns( oTable, {
		"iLeftWidth": 150,
		"fnDrawCallback": function ( left, right ) {
			var that = this, groupVal = null, matches = 0, heights = [], index = -1;
			
			/* Get the heights of the cells and remove redundant ones */
			$('tbody tr td', left.body).each( function ( i ) {
				var currVal = this.innerHTML;
				
				/* Reset values on new cell data. */
				if (currVal != groupVal) {
					groupVal = currVal;
					index++;
					heights[index] = 0;
					matches = 0;
				} else  {
					matches++;
				}
				
				heights[ index ] += $(this.parentNode).height();
				if ( currVal == groupVal && matches > 0 ) {
					this.parentNode.parentNode.removeChild(this.parentNode);
				}
			} );

			/* Now set the height of the cells which remain, from the summed heights */
			$('tbody tr td', left.body).each( function ( i ) {
				that.fnSetRowHeight( this.parentNode, heights[ i ] );
			} );
		}
	} );
} );
```

# Documentation

- Usage
- API / parameters

# Basic examples

- Basic demo of FixedColumns with zero initialisation
- Fix the left and right columns in place
- Index column attached to side of the table
- Index column, but with Y scrolling and no pagination
- Two columns fixed in place
- Fix the right column in place
- Speeding up row height matching with CSS
- jQuery UI themed table with FixedColumns

# Advanced examples

- Using ROWSPAN with FixedColumns
- Server-side processing with FixedColumns
- Individual column filtering
- Setting the fixed column width and resizing the table
- Setting the fixed column width with relative sizing
- Row grouping by grouping like rows in the fixed column
- Row grouping by inserting a grouping row

FixedColumns and DataTables © Allan Jardine 2011.
