# Supplementary material for: Molecule database framework: a framework for creating database applications with chemical structure search capability
Source: J Cheminform. 2013 Dec 11;5:48. doi: 10.1186/1758-2946-5-48 (PMC3892073; doi:10.1186/1758-2946-5-48)
Supplement: Additional file 4 — MDF simple web application source code of the mercurial changeset 16f39f4e447b. [file 1758-2946-5-48-S4.zip › src/main/webapp/resources/js/datatables/FixedColumns/x_y_scrolling.html]

FixedColumns example


FixedColumns example - X and Y scrolling

# Preamble

This example is basically the same as the index column example but in this case shows pagination disabled and Y scrolling in use.

# Live example

|  | Rendering engine | Browser | Platform(s) | Engine version | CSS grade |
| --- | --- | --- | --- | --- | --- |
| 1 | Trident | Internet Explorer 4.0 | Win 95+ | 4 | X |
| 2 | Trident | Internet Explorer 5.0 | Win 95+ | 5 | C |
| 3 | Trident | Internet Explorer 5.5 | Win 95+ | 5.5 | A |
| 4 | Trident | Internet Explorer 6 | Win 98+ | 6 | A |
| 5 | Trident | Internet Explorer 7 | Win XP SP2+ | 7 | A |
| 6 | Trident | AOL browser (AOL desktop) | Win XP | 6 | A |
| 7 | Gecko | Firefox 1.0 | Win 98+ / OSX.2+ | 1.7 | A |
| 8 | Gecko | Firefox 1.5 | Win 98+ / OSX.2+ | 1.8 | A |
| 9 | Gecko | Firefox 2.0 | Win 98+ / OSX.2+ | 1.8 | A |
| 10 | Gecko | Firefox 3.0 | Win 2k+ / OSX.3+ | 1.9 | A |
| 11 | Gecko | Camino 1.0 | OSX.2+ | 1.8 | A |
| 12 | Gecko | Camino 1.5 | OSX.3+ | 1.8 | A |
| 13 | Gecko | Netscape 7.2 | Win 95+ / Mac OS 8.6-9.2 | 1.7 | A |
| 14 | Gecko | Netscape Browser 8 | Win 98SE+ | 1.7 | A |
| 15 | Gecko | Netscape Navigator 9 | Win 98+ / OSX.2+ | 1.8 | A |
| 16 | Gecko | Mozilla 1.0 | Win 95+ / OSX.1+ | 1 | A |
| 17 | Gecko | Mozilla 1.1 | Win 95+ / OSX.1+ | 1.1 | A |
| 18 | Gecko | Mozilla 1.2 | Win 95+ / OSX.1+ | 1.2 | A |
| 19 | Gecko | Mozilla 1.3 | Win 95+ / OSX.1+ | 1.3 | A |
| 20 | Gecko | Mozilla 1.4 | Win 95+ / OSX.1+ | 1.4 | A |
| 21 | Gecko | Mozilla 1.5 | Win 95+ / OSX.1+ | 1.5 | A |
| 22 | Gecko | Mozilla 1.6 | Win 95+ / OSX.1+ | 1.6 | A |
| 23 | Gecko | Mozilla 1.7 | Win 98+ / OSX.1+ | 1.7 | A |
| 24 | Gecko | Mozilla 1.8 | Win 98+ / OSX.1+ | 1.8 | A |
| 25 | Gecko | Seamonkey 1.1 | Win 98+ / OSX.2+ | 1.8 | A |
| 26 | Gecko | Epiphany 2.20 | Gnome | 1.8 | A |
| 27 | Webkit | Safari 1.2 | OSX.3 | 125.5 | A |
| 28 | Webkit | Safari 1.3 | OSX.3 | 312.8 | A |
| 29 | Webkit | Safari 2.0 | OSX.4+ | 419.3 | A |
| 30 | Webkit | Safari 3.0 | OSX.4+ | 522.1 | A |
| 31 | Webkit | OmniWeb 5.5 | OSX.4+ | 420 | A |
| 32 | Webkit | iPod Touch / iPhone | iPod | 420.1 | A |
| 33 | Webkit | S60 | S60 | 413 | A |
| 34 | Presto | Opera 7.0 | Win 95+ / OSX.1+ | - | A |
| 35 | Presto | Opera 7.5 | Win 95+ / OSX.2+ | - | A |
| 36 | Presto | Opera 8.0 | Win 95+ / OSX.2+ | - | A |
| 37 | Presto | Opera 8.5 | Win 95+ / OSX.2+ | - | A |
| 38 | Presto | Opera 9.0 | Win 95+ / OSX.3+ | - | A |
| 39 | Presto | Opera 9.2 | Win 88+ / OSX.3+ | - | A |
| 40 | Presto | Opera 9.5 | Win 88+ / OSX.3+ | - | A |
| 41 | Presto | Opera for Wii | Wii | - | A |
| 42 | Presto | Nokia N800 | N800 | - | A |
| 43 | Presto | Nintendo DS browser | Nintendo DS | 8.5 | C/A |
| 44 | KHTML | Konqureror 3.1 | KDE 3.1 | 3.1 | C |
| 45 | KHTML | Konqureror 3.3 | KDE 3.3 | 3.3 | A |
| 46 | KHTML | Konqureror 3.5 | KDE 3.5 | 3.5 | A |
| 47 | Tasman | Internet Explorer 4.5 | Mac OS 8-9 | - | X |
| 48 | Tasman | Internet Explorer 5.1 | Mac OS 7.6-9 | 1 | C |
| 49 | Tasman | Internet Explorer 5.2 | Mac OS 8-X | 1 | C |
| 50 | Misc | NetFront 3.1 | Embedded devices | - | C |
| 51 | Misc | NetFront 3.4 | Embedded devices | - | A |
| 52 | Misc | Dillo 0.8 | Embedded devices | - | X |
| 53 | Misc | Links | Text only | - | X |
| 54 | Misc | Lynx | Text only | - | X |
| 55 | Misc | IE Mobile | Windows Mobile 6 | - | C |
| 56 | Misc | PSP browser | PSP | - | C |
| 57 | Other browsers | All others | - | - | U |

# Initialisation code

```
$(document).ready( function () {
	var oTable = $('#example').dataTable( {
		"sScrollY": "300px",
		"sScrollX": "100%",
		"sScrollXInner": "150%",
		"bScrollCollapse": true,
		"bPaginate": false,
		"fnDrawCallback": function ( oSettings ) {
			/* Need to redo the counters if filtered or sorted */
			if ( oSettings.bSorted || oSettings.bFiltered ) {
				for ( var i=0, iLen=oSettings.aiDisplay.length ; i<iLen ; i++ ) {
					var nTr = oSettings.aoData[ oSettings.aiDisplay[i] ].nTr;

					// Update the index column and do so without redrawing the table
					this.fnUpdate( i+1, nTr, 0, false, false );
				}
			}
		},
		"aoColumnDefs": [
			{ "bSortable": false, "sClass": "index", "aTargets": [ 0 ] }
		],
		"aaSorting": [[ 1, 'asc' ]]
	} );
	new FixedColumns( oTable );
} );
```

# Documentation

- Usage
- API / parameters

# Basic examples

- Basic demo of FixedColumns with zero initialisation
- Fix the left and right columns in place
- Index column attached to side of the table
- Index column, but with Y scrolling and no pagination
- Two columns fixed in place
- Fix the right column in place
- Speeding up row height matching with CSS
- jQuery UI themed table with FixedColumns

# Advanced examples

- Using ROWSPAN with FixedColumns
- Server-side processing with FixedColumns
- Individual column filtering
- Setting the fixed column width and resizing the table
- Setting the fixed column width with relative sizing
- Row grouping by grouping like rows in the fixed column
- Row grouping by inserting a grouping row

FixedColumns and DataTables © Allan Jardine 2011.
