# Supplementary material for: Molecule database framework: a framework for creating database applications with chemical structure search capability
Source: J Cheminform. 2013 Dec 11;5:48. doi: 10.1186/1758-2946-5-48 (PMC3892073; doi:10.1186/1758-2946-5-48)
Supplement: Additional file 4 — MDF simple web application source code of the mercurial changeset 16f39f4e447b. [file 1758-2946-5-48-S4.zip › src/main/webapp/resources/js/datatables/FixedHeader/top_bottom_left_right.html]

FixedHeader example


# FixedHeader example - top, bottom, left and right fixed

This example shows how FixedHeader can be made to look more like a spreadsheet application. Click 'View source' in your browser to see the set up.

|  | Rendering engine | Browser | Platform(s) | Engine version | CSS grade |  |
| --- | --- | --- | --- | --- | --- | --- |
|  | Trident | Internet Explorer 4.0 | Win 95+ | 4 | X |  |
|  | Trident | Internet Explorer 5.0 | Win 95+ | 5 | C |  |
|  | Trident | Internet Explorer 5.5 | Win 95+ | 5.5 | A |  |
|  | Trident | Internet Explorer 6 | Win 98+ | 6 | A |  |
|  | Trident | Internet Explorer 7 | Win XP SP2+ | 7 | A |  |
|  | Trident | AOL browser (AOL desktop) | Win XP | 6 | A |  |
|  | Gecko | Firefox 1.0 | Win 98+ / OSX.2+ | 1.7 | A |  |
|  | Gecko | Firefox 1.5 | Win 98+ / OSX.2+ | 1.8 | A |  |
|  | Gecko | Firefox 2.0 | Win 98+ / OSX.2+ | 1.8 | A |  |
|  | Gecko | Firefox 3.0 | Win 2k+ / OSX.3+ | 1.9 | A |  |
|  | Gecko | Camino 1.0 | OSX.2+ | 1.8 | A |  |
|  | Gecko | Camino 1.5 | OSX.3+ | 1.8 | A |  |
|  | Gecko | Netscape 7.2 | Win 95+ / Mac OS 8.6-9.2 | 1.7 | A |  |
|  | Gecko | Netscape Browser 8 | Win 98SE+ | 1.7 | A |  |
|  | Gecko | Netscape Navigator 9 | Win 98+ / OSX.2+ | 1.8 | A |  |
|  | Gecko | Mozilla 1.0 | Win 95+ / OSX.1+ | 1 | A |  |
|  | Gecko | Mozilla 1.1 | Win 95+ / OSX.1+ | 1.1 | A |  |
|  | Gecko | Mozilla 1.2 | Win 95+ / OSX.1+ | 1.2 | A |  |
|  | Gecko | Mozilla 1.3 | Win 95+ / OSX.1+ | 1.3 | A |  |
|  | Gecko | Mozilla 1.4 | Win 95+ / OSX.1+ | 1.4 | A |  |
|  | Gecko | Mozilla 1.5 | Win 95+ / OSX.1+ | 1.5 | A |  |
|  | Gecko | Mozilla 1.6 | Win 95+ / OSX.1+ | 1.6 | A |  |
|  | Gecko | Mozilla 1.7 | Win 98+ / OSX.1+ | 1.7 | A |  |
|  | Gecko | Mozilla 1.8 | Win 98+ / OSX.1+ | 1.8 | A |  |
|  | Gecko | Seamonkey 1.1 | Win 98+ / OSX.2+ | 1.8 | A |  |
|  | Gecko | Epiphany 2.20 | Gnome | 1.8 | A |  |
|  | Webkit | Safari 1.2 | OSX.3 | 125.5 | A |  |
|  | Webkit | Safari 1.3 | OSX.3 | 312.8 | A |  |
|  | Webkit | Safari 2.0 | OSX.4+ | 419.3 | A |  |
|  | Webkit | Safari 3.0 | OSX.4+ | 522.1 | A |  |
|  | Webkit | OmniWeb 5.5 | OSX.4+ | 420 | A |  |
|  | Webkit | iPod Touch / iPhone | iPod | 420.1 | A |  |
|  | Webkit | S60 | S60 | 413 | A |  |
|  | Presto | Opera 7.0 | Win 95+ / OSX.1+ | - | A |  |
|  | Presto | Opera 7.5 | Win 95+ / OSX.2+ | - | A |  |
|  | Presto | Opera 8.0 | Win 95+ / OSX.2+ | - | A |  |
|  | Presto | Opera 8.5 | Win 95+ / OSX.2+ | - | A |  |
|  | Presto | Opera 9.0 | Win 95+ / OSX.3+ | - | A |  |
|  | Presto | Opera 9.2 | Win 88+ / OSX.3+ | - | A |  |
|  | Presto | Opera 9.5 | Win 88+ / OSX.3+ | - | A |  |
|  | Presto | Opera for Wii | Wii | - | A |  |
|  | Presto | Nokia N800 | N800 | - | A |  |
|  | Presto | Nintendo DS browser | Nintendo DS | 8.5 | C/A^1^ |  |
|  | KHTML | Konqureror 3.1 | KDE 3.1 | 3.1 | C |  |
|  | KHTML | Konqureror 3.3 | KDE 3.3 | 3.3 | A |  |
|  | KHTML | Konqureror 3.5 | KDE 3.5 | 3.5 | A |  |
|  | Tasman | Internet Explorer 4.5 | Mac OS 8-9 | - | X |  |
|  | Tasman | Internet Explorer 5.1 | Mac OS 7.6-9 | 1 | C |  |
|  | Tasman | Internet Explorer 5.2 | Mac OS 8-X | 1 | C |  |
|  | Misc | NetFront 3.1 | Embedded devices | - | C |  |
|  | Misc | NetFront 3.4 | Embedded devices | - | A |  |
|  | Misc | Dillo 0.8 | Embedded devices | - | X |  |
|  | Misc | Links | Text only | - | X |  |
|  | Misc | Lynx | Text only | - | X |  |
|  | Misc | IE Mobile | Windows Mobile 6 | - | C |  |
|  | Misc | PSP browser | PSP | - | C |  |
|  | Other browsers | All others | - | - | U |  |
|  | Rendering engine | Browser | Platform(s) | Engine version | CSS grade |  |
| --- | --- | --- | --- | --- | --- | --- |
