# Supplementary material for: Molecule database framework: a framework for creating database applications with chemical structure search capability
Source: J Cheminform. 2013 Dec 11;5:48. doi: 10.1186/1758-2946-5-48 (PMC3892073; doi:10.1186/1758-2946-5-48)
Supplement: Additional file 4 — MDF simple web application source code of the mercurial changeset 16f39f4e447b. [file 1758-2946-5-48-S4.zip › src/main/webapp/resources/js/datatables/KeyTable/editing.html]

KeyTables editing example


KeyTable editing example

# Preamble

Possibly one of the most obvious applications of KeyTable is using it to navigate around a table, allowing a user to edit data as they go. This is an example of how that might be done through integration with the excellent jEditable plug-in for jQuery.

To edit data in the table, simply navigate to where you want to edit using the arrow keys, hit return and they make your edit. Hit return again to complete and submit the edit (note this example doesn't actually send the data to the server!) and then continue navigating.

# Live example

| Rendering engine | Browser | Platform(s) | Engine version | CSS grade |
| --- | --- | --- | --- | --- |
| Trident | Internet Explorer 4.0 | Win 95+ | 4 | X |
| Trident | Internet Explorer 5.0 | Win 95+ | 5 | C |
| Trident | Internet Explorer 5.5 | Win 95+ | 5.5 | A |
| Trident | Internet Explorer 6 | Win 98+ | 6 | A |
| Trident | Internet Explorer 7 | Win XP SP2+ | 7 | A |
| Trident | AOL browser (AOL desktop) | Win XP | 6 | A |
| Gecko | Firefox 1.0 | Win 98+ / OSX.2+ | 1.7 | A |
| Gecko | Firefox 1.5 | Win 98+ / OSX.2+ | 1.8 | A |
| Gecko | Firefox 2.0 | Win 98+ / OSX.2+ | 1.8 | A |
| Gecko | Firefox 3.0 | Win 2k+ / OSX.3+ | 1.9 | A |
| Gecko | Camino 1.0 | OSX.2+ | 1.8 | A |
| Gecko | Camino 1.5 | OSX.3+ | 1.8 | A |
| Gecko | Netscape 7.2 | Win 95+ / Mac OS 8.6-9.2 | 1.7 | A |
| Gecko | Netscape Browser 8 | Win 98SE+ | 1.7 | A |
| Gecko | Netscape Navigator 9 | Win 98+ / OSX.2+ | 1.8 | A |
| Gecko | Mozilla 1.0 | Win 95+ / OSX.1+ | 1 | A |
| Gecko | Mozilla 1.1 | Win 95+ / OSX.1+ | 1.1 | A |
| Gecko | Mozilla 1.2 | Win 95+ / OSX.1+ | 1.2 | A |
| Gecko | Mozilla 1.3 | Win 95+ / OSX.1+ | 1.3 | A |
| Gecko | Mozilla 1.4 | Win 95+ / OSX.1+ | 1.4 | A |
| Gecko | Mozilla 1.5 | Win 95+ / OSX.1+ | 1.5 | A |
| Gecko | Mozilla 1.6 | Win 95+ / OSX.1+ | 1.6 | A |
| Gecko | Mozilla 1.7 | Win 98+ / OSX.1+ | 1.7 | A |
| Gecko | Mozilla 1.8 | Win 98+ / OSX.1+ | 1.8 | A |
| Gecko | Seamonkey 1.1 | Win 98+ / OSX.2+ | 1.8 | A |
| Gecko | Epiphany 2.20 | Gnome | 1.8 | A |
| Webkit | Safari 1.2 | OSX.3 | 125.5 | A |
| Webkit | Safari 1.3 | OSX.3 | 312.8 | A |
| Webkit | Safari 2.0 | OSX.4+ | 419.3 | A |
| Webkit | Safari 3.0 | OSX.4+ | 522.1 | A |
| Webkit | OmniWeb 5.5 | OSX.4+ | 420 | A |
| Webkit | iPod Touch / iPhone | iPod | 420.1 | A |
| Webkit | S60 | S60 | 413 | A |
| Presto | Opera 7.0 | Win 95+ / OSX.1+ | - | A |
| Presto | Opera 7.5 | Win 95+ / OSX.2+ | - | A |
| Presto | Opera 8.0 | Win 95+ / OSX.2+ | - | A |
| Presto | Opera 8.5 | Win 95+ / OSX.2+ | - | A |
| Presto | Opera 9.0 | Win 95+ / OSX.3+ | - | A |
| Presto | Opera 9.2 | Win 88+ / OSX.3+ | - | A |
| Presto | Opera 9.5 | Win 88+ / OSX.3+ | - | A |
| Presto | Opera for Wii | Wii | - | A |
| Presto | Nokia N800 | N800 | - | A |
| Presto | Nintendo DS browser | Nintendo DS | 8.5 | C/A^1^ |
| KHTML | Konqureror 3.1 | KDE 3.1 | 3.1 | C |
| KHTML | Konqureror 3.3 | KDE 3.3 | 3.3 | A |
| KHTML | Konqureror 3.5 | KDE 3.5 | 3.5 | A |
| Tasman | Internet Explorer 4.5 | Mac OS 8-9 | - | X |
| Tasman | Internet Explorer 5.1 | Mac OS 7.6-9 | 1 | C |
| Tasman | Internet Explorer 5.2 | Mac OS 8-X | 1 | C |
| Misc | NetFront 3.1 | Embedded devices | - | C |
| Misc | NetFront 3.4 | Embedded devices | - | A |
| Misc | Dillo 0.8 | Embedded devices | - | X |
| Misc | Links | Text only | - | X |
| Misc | Lynx | Text only | - | X |
| Misc | IE Mobile | Windows Mobile 6 | - | C |
| Misc | PSP browser | PSP | - | C |
| Other browsers | All others | - | - | U |
| Rendering engine | Browser | Platform(s) | Engine version | CSS grade |
| --- | --- | --- | --- | --- |

# Initialisation code

```
$(document).ready( function () {
	var keys = new KeyTable( {
		"table": document.getElementById('example')
	} );
	
	/* Apply a return key event to each cell in the table */
	keys.event.action( null, null, function (nCell) {
		/* Block KeyTable from performing any events while jEditable is in edit mode */
		keys.block = true;
		
		/* Initialise the Editable instance for this table */
		$(nCell).editable( function (sVal) {
			/* Submit function (local only) - unblock KeyTable */
			keys.block = false;
			return sVal;
		}, { 
			"onblur": 'submit', 
			"onreset": function(){ 
				/* Unblock KeyTable, but only after this 'esc' key event has finished. Otherwise
				 * it will 'esc' KeyTable as well
				 */
				setTimeout( function () {keys.block = false;}, 0); 
			}
		} );
		
		/* Dispatch click event to go into edit mode - Saf 4 needs a timeout... */
		setTimeout( function () { $(nCell).click(); }, 0 );
	} );
} );
```

# Other examples

- Basic usage
- Editing a table
- Integration with an HTML form
- Integration with DataTables
- Using KeyTable with scrolling in DataTables

KeyTable © Allan Jardine 2009.  
Information in the table © U4EA Technologies 2007-2009.
