# Supplementary material for: Molecule database framework: a framework for creating database applications with chemical structure search capability
Source: J Cheminform. 2013 Dec 11;5:48. doi: 10.1186/1758-2946-5-48 (PMC3892073; doi:10.1186/1758-2946-5-48)
Supplement: Additional file 4 — MDF simple web application source code of the mercurial changeset 16f39f4e447b. [file 1758-2946-5-48-S4.zip › src/main/webapp/resources/js/datatables/KeyTable/form.html]

KeyTables example


KeyTable form integration example

# Preamble

One of possible use of KeyTable is to use a table as a form element (for example a calendar date selector). For this you want to be table to tab into and out of the table, as you would do with any other form element on the page.

The example shown below as a small table as it's third input element, and you can tab between input fields. When the 'focus' reaches the end of the table, hitting tab will take you into the next field.

# Live example

Input 1:

Input 2:

Input 3:

| Rendering engine | Browser | Platform(s) | Engine version | CSS grade |
| --- | --- | --- | --- | --- |
| Trident | Internet Explorer 4.0 | Win 95+ | 4 | X |
| Trident | Internet Explorer 5.0 | Win 95+ | 5 | C |
| Trident | Internet Explorer 5.5 | Win 95+ | 5.5 | A |
| Trident | Internet Explorer 6 | Win 98+ | 6 | A |
| Trident | Internet Explorer 7 | Win XP SP2+ | 7 | A |

Input 4:

Input 5:

# Initialisation code

```
$(document).ready( function () {
	var keys = new KeyTable( {
		"form": true
	} );
} );
```

# Other examples

- Basic usage
- Editing a table
- Integration with an HTML form
- Integration with DataTables
- Using KeyTable with scrolling in DataTables

KeyTable © Allan Jardine 2009.  
Information in the table © U4EA Technologies 2007-2009.
