# Supplementary material for: Molecule database framework: a framework for creating database applications with chemical structure search capability
Source: J Cheminform. 2013 Dec 11;5:48. doi: 10.1186/1758-2946-5-48 (PMC3892073; doi:10.1186/1758-2946-5-48)
Supplement: Additional file 4 — MDF simple web application source code of the mercurial changeset 16f39f4e447b. [file 1758-2946-5-48-S4.zip › src/main/webapp/resources/js/datatables/KeyTable/index.html]

KeyTables example


KeyTable example

# Preamble

KeyTable allows you to use keyboard navigation on an HTML table. The focused cell is shown through the CSS class ('focus') which in the case below is simply a blue border. Events such as 'focus', 'blur', 'esc' (the escape key) and 'return' (the return key) can be assigned event handling functions thought the API presented.

The example shown below has a few cells (selected at random, but near the top) with blur and focus events assigned to them. You can also see the navigation around the table using arrow keys.

# Live example

Event information:

| Rendering engine | Browser | Platform(s) | Engine version | CSS grade |
| --- | --- | --- | --- | --- |
| Trident | Internet Explorer 4.0 (focus once) | Win 95+ | 4 | X |
| Trident | Internet Explorer 5.0 | Win 95+ (blur) | 5 | C |
| Trident (focus) | Internet Explorer 5.5 | Win 95+ (action/bold) | 5.5 | A |
| Trident | Internet Explorer 6 (focus) | Win 98+ | 6 | A |
| Trident | Internet Explorer 7 | Win XP SP2+ (blur) | 7 | A |
| Trident | AOL browser (AOL desktop) | Win XP (action/italic) | 6 | A |
| Gecko | Firefox 1.0 | Win 98+ / OSX.2+ | 1.7 | A |
| Gecko (focus - row) | Firefox 1.5 | Win 98+ / OSX.2+ | 1.8 | A |
| Gecko | Firefox 2.0 | Win 98+ / OSX.2+ | 1.8 | A |
| Gecko | Firefox 3.0 | Win 2k+ / OSX.3+ | 1.9 | A |
| Gecko | Camino 1.0 | OSX.2+ | 1.8 | A |
| Gecko | Camino 1.5 | OSX.3+ | 1.8 | A |
| Gecko | Netscape 7.2 | Win 95+ / Mac OS 8.6-9.2 | 1.7 | A |
| Gecko | Netscape Browser 8 | Win 98SE+ | 1.7 | A |
| Gecko | Netscape Navigator 9 | Win 98+ / OSX.2+ | 1.8 | A |
| Gecko | Mozilla 1.0 | Win 95+ / OSX.1+ | 1 | A |
| Gecko | Mozilla 1.1 | Win 95+ / OSX.1+ | 1.1 | A |
| Gecko | Mozilla 1.2 | Win 95+ / OSX.1+ | 1.2 | A |
| Gecko | Mozilla 1.3 | Win 95+ / OSX.1+ | 1.3 | A |
| Gecko | Mozilla 1.4 | Win 95+ / OSX.1+ | 1.4 | A |
| Gecko | Mozilla 1.5 | Win 95+ / OSX.1+ | 1.5 | A |
| Gecko | Mozilla 1.6 | Win 95+ / OSX.1+ | 1.6 | A |
| Gecko | Mozilla 1.7 | Win 98+ / OSX.1+ | 1.7 | A |
| Gecko | Mozilla 1.8 | Win 98+ / OSX.1+ | 1.8 | A |
| Gecko | Seamonkey 1.1 | Win 98+ / OSX.2+ | 1.8 | A |
| Gecko | Epiphany 2.20 | Gnome | 1.8 | A |
| Webkit | Safari 1.2 | OSX.3 | 125.5 | A |
| Webkit | Safari 1.3 | OSX.3 | 312.8 | A |
| Webkit | Safari 2.0 | OSX.4+ | 419.3 | A |
| Webkit | Safari 3.0 | OSX.4+ | 522.1 | A |
| Webkit | OmniWeb 5.5 | OSX.4+ | 420 | A |
| Webkit | iPod Touch / iPhone | iPod | 420.1 | A |
| Webkit | S60 | S60 | 413 | A |
| Presto | Opera 7.0 | Win 95+ / OSX.1+ | - | A |
| Presto | Opera 7.5 | Win 95+ / OSX.2+ | - | A |
| Presto | Opera 8.0 | Win 95+ / OSX.2+ | - | A |
| Presto | Opera 8.5 | Win 95+ / OSX.2+ | - | A |
| Presto | Opera 9.0 | Win 95+ / OSX.3+ | - | A |
| Presto | Opera 9.2 | Win 88+ / OSX.3+ | - | A |
| Presto | Opera 9.5 | Win 88+ / OSX.3+ | - | A |
| Presto | Opera for Wii | Wii | - | A |
| Presto | Nokia N800 | N800 | - | A |
| Presto | Nintendo DS browser | Nintendo DS | 8.5 | C/A^1^ |
| KHTML | Konqureror 3.1 | KDE 3.1 | 3.1 | C |
| KHTML | Konqureror 3.3 | KDE 3.3 | 3.3 | A |
| KHTML | Konqureror 3.5 | KDE 3.5 | 3.5 | A |
| Tasman | Internet Explorer 4.5 | Mac OS 8-9 | - | X |
| Tasman | Internet Explorer 5.1 | Mac OS 7.6-9 | 1 | C |
| Tasman | Internet Explorer 5.2 | Mac OS 8-X | 1 | C |
| Misc | NetFront 3.1 | Embedded devices | - | C |
| Misc | NetFront 3.4 | Embedded devices | - | A |
| Misc | Dillo 0.8 | Embedded devices | - | X |
| Misc | Links | Text only | - | X |
| Misc | Lynx | Text only | - | X |
| Misc | IE Mobile | Windows Mobile 6 | - | C |
| Misc | PSP browser | PSP | - | C |
| Other browsers | All others | - | - | U |

# Initialisation code

```
function fnMessage ( sMesg )
{
	var n = document.getElementById('info');
	n.innerHTML += sMesg+"<br>";
	n.scrollTop = n.scrollHeight;
}

$(document).ready( function () {
	var keys = new KeyTable();
	
	/* Focus handler for all cells in last column */
	keys.event.focus( 4, null, function( nNode, x, y ) {
		fnMessage( "Cell "+x+","+y+" focused ('live' event - column)" );
	} );
	
	/* Focus handler for all cells in 8th row */
	keys.event.focus( null, 7, function( nNode, x, y ) {
		fnMessage( "Cell "+x+","+y+" focused ('live' event - row)" );
	} );
	
	/* Focus using coords. */
	keys.event.focus( 1, 0, function( nNode ) {
		keys.event.remove.focus( nNode );
		fnMessage( "Cell 1,0 focus - this event has now been removed" );
	} );
	
	keys.event.focus( 1, 3, function() {
		fnMessage( "Cell 1,3 focus" );
	} );
	
	/* focus with a node */
	keys.event.focus( $('#example tbody tr:eq(2) td:eq(0)')[0], function() {
		fnMessage( "Cell 0,2 focus" );
	} );
	
	/* Blur using a node */
	keys.event.blur( $('#example tbody tr:eq(1) td:eq(2)')[0], function() {
		fnMessage( "Cell 1,2 blur" );
	} );
	
	/* Blur using coords */
	keys.event.blur( 2, 4, function() {
		fnMessage( "Cell 2,4 blur" );
	} );
	
	/* Action */
	keys.event.action( 2, 2, function( nNode ) {
		fnMessage( "Cell 2,2 action" );
		if ( nNode.style.fontWeight == "" || nNode.style.fontWeight == "normal" ) {
			nNode.style.fontWeight = "bold";
		} else {
			nNode.style.fontWeight = "normal";
		}
	} );
	
	keys.event.action( 2, 5, function( nNode ) {
		fnMessage( "Cell 2,5 action" );
		if ( nNode.style.fontStyle == "" ) {
			nNode.style.fontStyle = "italic";
		} else {
			nNode.style.fontStyle = "";
		}
	} );
} );
```

# Other examples

- Basic usage
- Editing a table
- Integration with an HTML form
- Integration with DataTables
- Using KeyTable with scrolling in DataTables

KeyTable © Allan Jardine 2009.  
Information in the table © U4EA Technologies 2007-2009.
