# Supplementary material for: Molecule database framework: a framework for creating database applications with chemical structure search capability
Source: J Cheminform. 2013 Dec 11;5:48. doi: 10.1186/1758-2946-5-48 (PMC3892073; doi:10.1186/1758-2946-5-48)
Supplement: Additional file 4 — MDF simple web application source code of the mercurial changeset 16f39f4e447b. [file 1758-2946-5-48-S4.zip › src/main/webapp/resources/js/datatables/Scroller/api_scrolling.html]

DataTables example


Scroller API example

# Preamble

This example shows a trivial use of the Scroller API to scroll the table to row 1000 once the table has been loaded. Any number could be used here, and a small animated effect is used to provide visual feedback to the user (this can be disabled by passed false as a second parameter to fnScrollToRow).

# Live example

| ID | First name | Last name | ZIP | Country |
| --- | --- | --- | --- | --- |

# Initialisation code

```
$(document).ready(function() {
	$('#example').dataTable( {
		"sScrollY": "200px",
		"sAjaxSource": "media/data/2500.txt",
		"sDom": "frtiS",
		"bDeferRender": true,
		"fnInitComplete": function () {
			this.fnSettings().oScroller.fnScrollToRow( 1000 );
		}
	} );
} );
```

# Other examples

- Basic initialisation of Scroller
- State saving
- API - pragmatically move to a row
- Server-side processing with Scroller
- 50'000 rows in a table - client-side generated data
