# Supplementary material for: Molecule database framework: a framework for creating database applications with chemical structure search capability
Source: J Cheminform. 2013 Dec 11;5:48. doi: 10.1186/1758-2946-5-48 (PMC3892073; doi:10.1186/1758-2946-5-48)
Supplement: Additional file 4 — MDF simple web application source code of the mercurial changeset 16f39f4e447b. [file 1758-2946-5-48-S4.zip › src/main/webapp/resources/js/datatables/Scroller/index.html]

DataTables example


Scroller basic initialisation example

# Preamble

Scroller is a plug-in for DataTables which enhances the "infinite scrolling" feature that DataTables has built-in to make the scrolling feel more natural. The scrolling box shown on the page is the full height of the drawn table, but a virtual rendering technique is used to only render the part of the table that is actually viewable in the scrolling viewport in order to increase the speed of drawing.

Note that Scroller assumes that all rows are of the same height (in order to preform the required calculations. You can use 'td { white-space: nowrap; }' in your CSS to ensure that text in rows does not wrap.

This example shows how Scroller for DataTables can be initialised by simply including the character 'S' in sDom (note that the 'S' must come after the 't' in sDom). Deferred rendering an and Ajax data source are also used in this example.

# Live example

| ID | First name | Last name | ZIP | Country |
| --- | --- | --- | --- | --- |

# Initialisation code

```
$(document).ready(function() {
	var oTable = $('#example').dataTable( {
		"sScrollY": "200px",
		"sAjaxSource": "media/data/2500.txt",
		"sDom": "frtiS",
		"bDeferRender": true
	} );
} );
```

# Other examples

- Basic initialisation of Scroller
- State saving
- API - pragmatically move to a row
- Server-side processing with Scroller
- 50'000 rows in a table - client-side generated data
