# Supplementary material for: Molecule database framework: a framework for creating database applications with chemical structure search capability
Source: J Cheminform. 2013 Dec 11;5:48. doi: 10.1186/1758-2946-5-48 (PMC3892073; doi:10.1186/1758-2946-5-48)
Supplement: Additional file 4 — MDF simple web application source code of the mercurial changeset 16f39f4e447b. [file 1758-2946-5-48-S4.zip › src/main/webapp/resources/js/datatables/Scroller/large_js_source.html]

DataTables example


Scroller example - 50'000 rows

# Preamble

This example is completely artificial in that the data generated is created on the client-side by just looping around a Javascript array and then passing that to DataTables. However, it does show quite nicely that DataTables and Scroller can cope with large amounts of data on the client-side quite nicely. Typically data such as this would be Ajax sourced and server-side processing should be considered. Please be aware that the performance of this page will depend on your browser - for example IE6 will crawl!

# Live example

| Column 1 | Column 2 | Column 3 | Column 4 | Column 5 |
| --- | --- | --- | --- | --- |

# Initialisation code

```
$(document).ready(function() {
	var aaData = [];
	for ( var i=1 ; i<50000 ; i++ ) {
		aaData.push( [ i, i, i, i, i ] );
	}
	
	var oTable = $('#example').dataTable( {
		"sScrollY": "200px",
		"aaData": aaData,
		"sDom": "frtiS",
		"bDeferRender": true
	} );
} );
```

# Other examples

- Basic initialisation of Scroller
- State saving
- API - pragmatically move to a row
- Server-side processing with Scroller
- 50'000 rows in a table - client-side generated data
