# Supplementary material for: Molecule database framework: a framework for creating database applications with chemical structure search capability
Source: J Cheminform. 2013 Dec 11;5:48. doi: 10.1186/1758-2946-5-48 (PMC3892073; doi:10.1186/1758-2946-5-48)
Supplement: Additional file 4 — MDF simple web application source code of the mercurial changeset 16f39f4e447b. [file 1758-2946-5-48-S4.zip › src/main/webapp/resources/js/datatables/Scroller/media/docs/baed189d4a.html]

Namespace: s - documentation


# Namespace: s

## Ancestry: Scroller# » s

Scroller v1.1.0 documentation

## Navigation

- Overview
- Summary

  Properties
- Details

  Properties

Hiding private elements
(toggle)

Showing extended elements
(toggle)

Settings object which contains customisable information for the Scroller instance

### Extends

- Scroller.DEFAULTS

## Summary

### Properties

<static> autoHeight :bool
:   Auto row height or not indicator

<static> drawTO :int
:   setTimeout reference for the redraw, used when server-side processing is enabled in the
    DataTables in order to prevent DoSing the server

<static> dt :object
:   DataTables settings object

<static> rowHeight :int
:   Height of rows in the table

<static> stateTO :int
:   setTimeout reference for state saving, used when state saving is enabled in the DataTable
    and when the user scrolls the viewport in order to stop the cookie set taking too much
    CPU!

<static> tableBottom :int
:   Pixel location of the bottom of the drawn table in the viewport

<static> tableTop :int
:   Pixel location of the top of the drawn table in the viewport

<static> viewportHeight :int
:   Pixel height of the viewport

<static> viewportRows :int
:   Number of rows calculated as visible in the visible viewport

## Details

### Properties

<static> autoHeight :bool
:   Auto row height or not indicator

<static> drawTO :int
:   setTimeout reference for the redraw, used when server-side processing is enabled in the
    DataTables in order to prevent DoSing the server

<static> dt :object
:   DataTables settings object

<static> rowHeight :int
:   Height of rows in the table

<static> stateTO :int
:   setTimeout reference for state saving, used when state saving is enabled in the DataTable
    and when the user scrolls the viewport in order to stop the cookie set taking too much
    CPU!

<static> tableBottom :int
:   Pixel location of the bottom of the drawn table in the viewport

<static> tableTop :int
:   Pixel location of the top of the drawn table in the viewport

<static> viewportHeight :int
:   Pixel height of the viewport

<static> viewportRows :int
:   Number of rows calculated as visible in the visible viewport

Scroller: Copyright 2011-2012 Allan Jardine, all rights reserved  
Documentation generated by JSDoc 3 on
22th Jun 2012 - 08:22
with the DataTables template.
