# Supplementary material for: Molecule database framework: a framework for creating database applications with chemical structure search capability
Source: J Cheminform. 2013 Dec 11;5:48. doi: 10.1186/1758-2946-5-48 (PMC3892073; doi:10.1186/1758-2946-5-48)
Supplement: Additional file 4 — MDF simple web application source code of the mercurial changeset 16f39f4e447b. [file 1758-2946-5-48-S4.zip › src/main/webapp/resources/js/datatables/Scroller/media/docs/index.html]

Table of Contents - documentation


### Table of Contents

Scroller
:   Scroller is a virtual rendering plug-in for DataTables which allows large
    datasets to be drawn on screen every quickly. What the virtual rendering means
    is that only the visible portion of the table (and a bit to either side to make
    the scrolling smooth) is drawn, while the scrolling container gives the
    visual impression that the whole table is visible. This is done by making use
    of the pagination abilities of DataTables and moving the table around in the
    scrolling container DataTables adds to the page. The scrolling container is
    forced to the height it would be for the full table display using an extra
    element.   
      
    Note that rows in the table MUST all be the same height. Information in a cell
    which expands on to multiple lines will cause some odd behaviour in the scrolling.  
      
    Scroller is initialised by simply including the letter 'S' in the sDom for the
    table you want to have this feature enabled on. Note that the 'S' must come
    AFTER the 't' parameter in sDom.  
      
    Key features include:

    - Speed! The aim of Scroller for DataTables is to make rendering large data sets fast
    - Full compatibility with deferred rendering in DataTables 1.9 for maximum speed
    - Correct visual scrolling implementation, similar to "infinite scrolling" in DataTable core
    - Integration with state saving in DataTables (scrolling position is saved)
    - Easy to use

Scroller: Copyright 2011-2012 Allan Jardine, all rights reserved  
Documentation generated by JSDoc 3 on
22th Jun 2012 - 08:22
with the DataTables template.
