# Supplementary material for: Molecule database framework: a framework for creating database applications with chemical structure search capability
Source: J Cheminform. 2013 Dec 11;5:48. doi: 10.1186/1758-2946-5-48 (PMC3892073; doi:10.1186/1758-2946-5-48)
Supplement: Additional file 4 — MDF simple web application source code of the mercurial changeset 16f39f4e447b. [file 1758-2946-5-48-S4.zip › src/main/webapp/resources/js/datatables/Scroller/media/docs/Scroller.html]

Class: Scroller - documentation


# Class: Scroller

Scroller v1.1.0 documentation

## Navigation

- Overview
- Summary

  Namespaces | Properties | Methods
- Details

  Properties | Methods

Hiding private elements
(toggle)

Showing extended elements
(toggle)

new Scroller(oDT, oOpts)
:   Scroller is a virtual rendering plug-in for DataTables which allows large
    datasets to be drawn on screen every quickly. What the virtual rendering means
    is that only the visible portion of the table (and a bit to either side to make
    the scrolling smooth) is drawn, while the scrolling container gives the
    visual impression that the whole table is visible. This is done by making use
    of the pagination abilities of DataTables and moving the table around in the
    scrolling container DataTables adds to the page. The scrolling container is
    forced to the height it would be for the full table display using an extra
    element.   
      
    Note that rows in the table MUST all be the same height. Information in a cell
    which expands on to multiple lines will cause some odd behaviour in the scrolling.  
      
    Scroller is initialised by simply including the letter 'S' in the sDom for the
    table you want to have this feature enabled on. Note that the 'S' must come
    AFTER the 't' parameter in sDom.  
      
    Key features include:

    - Speed! The aim of Scroller for DataTables is to make rendering large data sets fast
    - Full compatibility with deferred rendering in DataTables 1.9 for maximum speed
    - Correct visual scrolling implementation, similar to "infinite scrolling" in DataTable core
    - Integration with state saving in DataTables (scrolling position is saved)
    - Easy to use

    ### Constructor

    ##### Parameters:

    |  | Name | Type | Attributes | Default | Description |
    | --- | --- | --- | --- | --- | --- |
    | 1 | oDT | object |  |  | DataTables settings object |
    | 2 | oOpts | object | Optional | {} | Configuration object for FixedColumns. Options are defined by Scroller.oDefaults |

    ##### Example:

    ```
    		$(document).ready(function() {
    			$('#example').dataTable( {
    				"sScrollY": "200px",
    				"sAjaxSource": "media/dataset/large.txt",
    				"sDom": "frtiS",
    				"bDeferRender": true
    			} );
    		} );
    ```

### Requires

- module:jQuery
- module:DataTables

## Summary

### Namespaces

dom
:   DOM elements used by the class instance

oDefaults
:   Scroller default settings for initialisation

s
:   Settings object which contains customisable information for the Scroller instance

### Properties

CLASS :String
:   Name of this class

<static> VERSION :String
:   Scroller version

### Methods

fnMeasure(bRedraw) → {void}
:   Calculate and store information about how many rows are to be displayed in the scrolling
    viewport, based on current dimensions in the browser's rendering. This can be particularly
    useful if the table is initially drawn in a hidden element - for example in a tab.

fnPixelsToRow(iPixels) → {int}
:   Calculate the row number that will be found at the given pixel position (y-scroll)

fnRowToPixels(iRow) → {int}
:   Calculate the pixel position from the top of the scrolling container for a given row

fnScrollToRow(iRow, bAnimate) → {void}
:   Calculate the row number that will be found at the given pixel position (y-scroll)

## Details

### Properties

CLASS :String
:   Name of this class

<static> VERSION :String
:   Scroller version

### Methods

fnMeasure(bRedraw) → {void}
:   Calculate and store information about how many rows are to be displayed in the scrolling
    viewport, based on current dimensions in the browser's rendering. This can be particularly
    useful if the table is initially drawn in a hidden element - for example in a tab.

    ##### Parameters:

    |  | Name | Type | Attributes | Default | Description |
    | --- | --- | --- | --- | --- | --- |
    | 1 | bRedraw | bool | Optional | true | Redraw the table automatically after the recalculation, with the new dimentions forming the basis for the draw. |

    ##### Example:

    ```
       $(document).ready(function() {
         // Make the example container hidden to throw off the browser's sizing
         document.getElementById('container').style.display = "none";
         var oTable = $('#example').dataTable( {
           "sScrollY": "200px",
           "sAjaxSource": "media/dataset/large.txt",
           "sDom": "frtiS",
           "bDeferRender": true,
           "fnInitComplete": function (o) {
             // Immediately scroll to row 1000
             o.oScroller.fnScrollToRow( 1000 );
           }
         } );
         
         setTimeout( function () {
           // Make the example container visible and recalculate the scroller sizes
           document.getElementById('container').style.display = "block";
           oTable.fnSettings().oScroller.fnMeasure();
         }, 3000 );
    ```

    fnPixelsToRow(iPixels) → {int}
    :   Calculate the row number that will be found at the given pixel position (y-scroll)

        ##### Parameters:

        |  | Name | Type | Attributes | Default | Description |
        | --- | --- | --- | --- | --- | --- |
        | 1 | iPixels | int |  |  | Offset from top to caluclate the row number of |

        ##### Returns:

        Row index

        ##### Example:

        ```
           $(document).ready(function() {
             $('#example').dataTable( {
               "sScrollY": "200px",
               "sAjaxSource": "media/dataset/large.txt",
               "sDom": "frtiS",
               "bDeferRender": true,
               "fnInitComplete": function (o) {
                 // Find what row number is at 500px
                 alert( o.oScroller.fnPixelsToRow( 500 ) );
               }
             } );
           } );
        ```

        fnRowToPixels(iRow) → {int}
        :   Calculate the pixel position from the top of the scrolling container for a given row

            ##### Parameters:

            |  | Name | Type | Attributes | Default | Description |
            | --- | --- | --- | --- | --- | --- |
            | 1 | iRow | int |  |  | Row number to calculate the position of |

            ##### Returns:

            Pixels

            ##### Example:

            ```
               $(document).ready(function() {
                 $('#example').dataTable( {
                   "sScrollY": "200px",
                   "sAjaxSource": "media/dataset/large.txt",
                   "sDom": "frtiS",
                   "bDeferRender": true,
                   "fnInitComplete": function (o) {
                     // Find where row 25 is
                     alert( o.oScroller.fnRowToPixels( 25 ) );
                   }
                 } );
               } );
            ```

            fnScrollToRow(iRow, bAnimate) → {void}
            :   Calculate the row number that will be found at the given pixel position (y-scroll)

                ##### Parameters:

                |  | Name | Type | Attributes | Default | Description |
                | --- | --- | --- | --- | --- | --- |
                | 1 | iRow | int |  |  | Row index to scroll to |
                | 2 | bAnimate | bool | Optional | true | Animate the transision or not |

                ##### Example:

                ```
                   $(document).ready(function() {
                     $('#example').dataTable( {
                       "sScrollY": "200px",
                       "sAjaxSource": "media/dataset/large.txt",
                       "sDom": "frtiS",
                       "bDeferRender": true,
                       "fnInitComplete": function (o) {
                         // Immediately scroll to row 1000
                         o.oScroller.fnScrollToRow( 1000 );
                       }
                     } );
                     
                     // Sometime later on use the following to scroll to row 500...
                         var oSettings = $('#example').dataTable().fnSettings();
                     oSettings.oScroller.fnScrollToRow( 500 );
                   } );
                ```

Scroller: Copyright 2011-2012 Allan Jardine, all rights reserved  
Documentation generated by JSDoc 3 on
22th Jun 2012 - 08:22
with the DataTables template.
