# Supplementary material for: Molecule database framework: a framework for creating database applications with chemical structure search capability
Source: J Cheminform. 2013 Dec 11;5:48. doi: 10.1186/1758-2946-5-48 (PMC3892073; doi:10.1186/1758-2946-5-48)
Supplement: Additional file 4 — MDF simple web application source code of the mercurial changeset 16f39f4e447b. [file 1758-2946-5-48-S4.zip › src/main/webapp/resources/js/datatables/Scroller/media/docs/Scroller.oDefaults.html]

Namespace: oDefaults - documentation


# Namespace: oDefaults

## Ancestry: Scroller. » oDefaults

Scroller v1.1.0 documentation

## Navigation

- Overview
- Summary

  Properties
- Details

  Properties

Hiding private elements
(toggle)

Showing extended elements
(toggle)

Scroller default settings for initialisation

## Summary

### Properties

<static> boundaryScale :float
:   Scroller uses the boundary scaling factor to decide when to redraw the table - which it
    typically does before you reach the end of the currently loaded data set (in order to
    allow the data to look continuous to a user scrolling through the data). If given as 0
    then the table will be redrawn whenever the viewport is scrolled, while 1 would not
    redraw the table until the currently loaded data has all been shown. You will want
    something in the middle - the default factor of 0.5 is usually suitable.

<static> displayBuffer :int
:   The display buffer is what Scroller uses to calculate how many rows it should pre-fetch
    for scrolling. Scroller automatically adjusts DataTables' display length to pre-fetch
    rows that will be shown in "near scrolling" (i.e. just beyond the current display area).
    The value is based upon the number of rows that can be displayed in the viewport (i.e.
    a value of 1), and will apply the display range to records before before and after the
    current viewport - i.e. a factor of 3 will allow Scroller to pre-fetch 1 viewport's worth
    of rows before the current viewport, the current viewport's rows and 1 viewport's worth
    of rows after the current viewport. Adjusting this value can be useful for ensuring
    smooth scrolling based on your data set.

<static> loadingIndicator :boolean
:   Show (or not) the loading element in the background of the table. Note that you should
    include the dataTables.scroller.css file for this to be displayed correctly.

<static> rowHeight :int|string
:   Scroller will attempt to automatically calculate the height of rows for it's internal
    calculations. However the height that is used can be overridden using this parameter.

<static> serverWait :int
:   When using server-side processing, Scroller will wait a small amount of time to allow
    the scrolling to finish before requesting more data from the server. This prevents
    you from DoSing your own server! The wait time can be configured by this parameter.

<static> trace :bool
:   Indicate if Scroller show show trace information on the console or not. This can be
    useful when debugging Scroller or if just curious as to what it is doing, but should
    be turned off for production.

## Details

### Properties

<static> boundaryScale :float
:   Scroller uses the boundary scaling factor to decide when to redraw the table - which it
    typically does before you reach the end of the currently loaded data set (in order to
    allow the data to look continuous to a user scrolling through the data). If given as 0
    then the table will be redrawn whenever the viewport is scrolled, while 1 would not
    redraw the table until the currently loaded data has all been shown. You will want
    something in the middle - the default factor of 0.5 is usually suitable.

    ##### Example

    ```
       var oTable = $('#example').dataTable( {
           "sScrollY": "200px",
           "sDom": "frtiS",
           "bDeferRender": true,
           "oScroller": {
             "boundaryScale": 0.75
           }
       } );
    ```

<static> displayBuffer :int
:   The display buffer is what Scroller uses to calculate how many rows it should pre-fetch
    for scrolling. Scroller automatically adjusts DataTables' display length to pre-fetch
    rows that will be shown in "near scrolling" (i.e. just beyond the current display area).
    The value is based upon the number of rows that can be displayed in the viewport (i.e.
    a value of 1), and will apply the display range to records before before and after the
    current viewport - i.e. a factor of 3 will allow Scroller to pre-fetch 1 viewport's worth
    of rows before the current viewport, the current viewport's rows and 1 viewport's worth
    of rows after the current viewport. Adjusting this value can be useful for ensuring
    smooth scrolling based on your data set.

    ##### Example

    ```
       var oTable = $('#example').dataTable( {
           "sScrollY": "200px",
           "sDom": "frtiS",
           "bDeferRender": true,
           "oScroller": {
             "displayBuffer": 10
           }
       } );
    ```

<static> loadingIndicator :boolean
:   Show (or not) the loading element in the background of the table. Note that you should
    include the dataTables.scroller.css file for this to be displayed correctly.

    ##### Example

    ```
       var oTable = $('#example').dataTable( {
           "sScrollY": "200px",
           "sDom": "frtiS",
           "bDeferRender": true,
           "oScroller": {
             "loadingIndicator": true
           }
       } );
    ```

<static> rowHeight :int|string
:   Scroller will attempt to automatically calculate the height of rows for it's internal
    calculations. However the height that is used can be overridden using this parameter.

    ##### Example

    ```
       var oTable = $('#example').dataTable( {
           "sScrollY": "200px",
           "sDom": "frtiS",
           "bDeferRender": true,
           "oScroller": {
             "rowHeight": 30
           }
       } );
    ```

<static> serverWait :int
:   When using server-side processing, Scroller will wait a small amount of time to allow
    the scrolling to finish before requesting more data from the server. This prevents
    you from DoSing your own server! The wait time can be configured by this parameter.

    ##### Example

    ```
       var oTable = $('#example').dataTable( {
           "sScrollY": "200px",
           "sDom": "frtiS",
           "bDeferRender": true,
           "oScroller": {
             "serverWait": 100
           }
       } );
    ```

<static> trace :bool
:   Indicate if Scroller show show trace information on the console or not. This can be
    useful when debugging Scroller or if just curious as to what it is doing, but should
    be turned off for production.

    ##### Example

    ```
       var oTable = $('#example').dataTable( {
           "sScrollY": "200px",
           "sDom": "frtiS",
           "bDeferRender": true,
           "oScroller": {
             "trace": true
           }
       } );
    ```

Scroller: Copyright 2011-2012 Allan Jardine, all rights reserved  
Documentation generated by JSDoc 3 on
22th Jun 2012 - 08:22
with the DataTables template.
