# Supplementary material for: Molecule database framework: a framework for creating database applications with chemical structure search capability
Source: J Cheminform. 2013 Dec 11;5:48. doi: 10.1186/1758-2946-5-48 (PMC3892073; doi:10.1186/1758-2946-5-48)
Supplement: Additional file 4 — MDF simple web application source code of the mercurial changeset 16f39f4e447b. [file 1758-2946-5-48-S4.zip › src/main/webapp/resources/js/datatables/Scroller/server-side_processing.html]

DataTables example


Scroller example - server-side processing

# Preamble

When using server-side processing, Scroller will wait a small amount of time to allow the scrolling to finish before requesting more data from the server (200mS by default). This prevents you from DoSing your own server! This example shows Scroller in action with a database of 10'000 records, although of course server-side processing can scale much larger if you require. In this example we also enable the *loadingIndicator* option of Scroller to show the end user what is happening when they scroll passed the currently loaded data.

# Live example

| ID | First name | Last name | ZIP | Country |
| --- | --- | --- | --- | --- |

# Initialisation code

```
$(document).ready(function() {
	var oTable = $('#example').dataTable( {
		"sScrollY": "200px",
		"sAjaxSource": "media/data/server_processing.php",
		"bServerSide": true,
		"sDom": "frtiS",
		"oScroller": {
			"loadingIndicator": true
		}
	} );
} );
```

# Other examples

- Basic initialisation of Scroller
- State saving
- API - pragmatically move to a row
- Server-side processing with Scroller
- 50'000 rows in a table - client-side generated data
