# Supplementary material for: Molecule database framework: a framework for creating database applications with chemical structure search capability
Source: J Cheminform. 2013 Dec 11;5:48. doi: 10.1186/1758-2946-5-48 (PMC3892073; doi:10.1186/1758-2946-5-48)
Supplement: Additional file 4 — MDF simple web application source code of the mercurial changeset 16f39f4e447b. [file 1758-2946-5-48-S4.zip › src/main/webapp/resources/js/datatables/Scroller/state_saving.html]

DataTables example


Scroller with state saving

# Preamble

Scroller will automatically integrate with DataTables in order to save the scrolling position of the table, if state saving is enabled in the DataTable (bStateSave). This example shows that in practice - to demonstrate, scroll the table and then reload the page.

# Live example

| ID | First name | Last name | ZIP | Country |
| --- | --- | --- | --- | --- |

# Initialisation code

```
$(document).ready(function() {
	var oTable = $('#example').dataTable( {
		"sScrollY": "200px",
		"sAjaxSource": "media/data/2500.txt",
		"sDom": "frtiS",
		"bDeferRender": true,
		"bStateSave": true
	} );
} );
```

# Other examples

- Basic initialisation of Scroller
- State saving
- API - pragmatically move to a row
- Server-side processing with Scroller
- 50'000 rows in a table - client-side generated data
