# Supplementary material for: Molecule database framework: a framework for creating database applications with chemical structure search capability
Source: J Cheminform. 2013 Dec 11;5:48. doi: 10.1186/1758-2946-5-48 (PMC3892073; doi:10.1186/1758-2946-5-48)
Supplement: Additional file 4 — MDF simple web application source code of the mercurial changeset 16f39f4e447b. [file 1758-2946-5-48-S4.zip › src/main/webapp/resources/js/datatables/TableTools/multi_instance.html]

TableTools example


TableTools example - multiple toolbars

# Preamble

Like all DataTables control elements, TableTools can have multiple instances specified in the *sDom* parameter of DataTables. This will create two TableTools toolbars next to the table, providing the same functions. An example of when this might be useful is to show the toolbar both above and below the table - as is done in this example.

# Live example

| Rendering engine | Browser | Platform(s) | Engine version | CSS grade |
| --- | --- | --- | --- | --- |
| Rendering engine | Browser | Platform(s) | Engine version | CSS grade |
| --- | --- | --- | --- | --- |
| Trident | Internet Explorer 4.0 | Win 95+ (Entity: &) | 4 | X |
| Trident | Internet Explorer 5.0 | Win 95+ | 5 | C |
| Trident | Internet Explorer 5.5 | Win 95+ | 5.5 | A |
| Trident | Internet Explorer 6 | Win 98+ | 6 | A |
| Trident | Internet Explorer 7 | Win XP SP2+ | 7 | A |
| Trident | AOL browser (AOL desktop) | Win XP | 6 | A |
| Gecko (UTF-8: $¢€) | Firefox 1.0 | Win 98+ / OSX.2+ | 1.7 | A |
| Gecko | Firefox 1.5 | Win 98+ / OSX.2+ | 1.8 | A |
| Gecko | Firefox 2.0 | Win 98+ / OSX.2+ | 1.8 | A |
| Gecko | Firefox 3.0 | Win 2k+ / OSX.3+ | 1.9 | A |
| Gecko | Camino 1.0 | OSX.2+ | 1.8 | A |
| Gecko | Camino 1.5 | OSX.3+ | 1.8 | A |
| Gecko | Netscape 7.2 | Win 95+ / Mac OS 8.6-9.2 | 1.7 | A |
| Gecko | Netscape Browser 8 | Win 98SE+ | 1.7 | A |
| Gecko | Netscape Navigator 9 | Win 98+ / OSX.2+ | 1.8 | A |
| Gecko | Mozilla 1.0 | Win 95+ / OSX.1+ | 1 | A |
| Gecko | Mozilla 1.1 | Win 95+ / OSX.1+ | 1.1 | A |
| Gecko | Mozilla 1.2 | Win 95+ / OSX.1+ | 1.2 | A |
| Gecko | Mozilla 1.3 | Win 95+ / OSX.1+ | 1.3 | A |
| Gecko | Mozilla 1.4 | Win 95+ / OSX.1+ | 1.4 | A |
| Gecko | Mozilla 1.5 | Win 95+ / OSX.1+ | 1.5 | A |
| Gecko | Mozilla 1.6 | Win 95+ / OSX.1+ | 1.6 | A |
| Gecko | Mozilla 1.7 | Win 98+ / OSX.1+ | 1.7 | A |
| Gecko | Mozilla 1.8 | Win 98+ / OSX.1+ | 1.8 | A |
| Gecko | Seamonkey 1.1 | Win 98+ / OSX.2+ | 1.8 | A |
| Gecko | Epiphany 2.20 | Gnome | 1.8 | A |
| Webkit | Safari 1.2 | OSX.3 | 125.5 | A |
| Webkit | Safari 1.3 | OSX.3 | 312.8 | A |
| Webkit | Safari 2.0 | OSX.4+ | 419.3 | A |
| Webkit | Safari 3.0 | OSX.4+ | 522.1 | A |
| Webkit | OmniWeb 5.5 | OSX.4+ | 420 | A |
| Webkit | iPod Touch / iPhone | iPod | 420.1 | A |
| Webkit | S60 | S60 | 413 | A |
| Presto | Opera 7.0 | Win 95+ / OSX.1+ | - | A |
| Presto | Opera 7.5 | Win 95+ / OSX.2+ | - | A |
| Presto | Opera 8.0 | Win 95+ / OSX.2+ | - | A |
| Presto | Opera 8.5 | Win 95+ / OSX.2+ | - | A |
| Presto | Opera 9.0 | Win 95+ / OSX.3+ | - | A |
| Presto | Opera 9.2 | Win 88+ / OSX.3+ | - | A |
| Presto | Opera 9.5 | Win 88+ / OSX.3+ | - | A |
| Presto | Opera for Wii | Wii | - | A |
| Presto | Nokia N800 | N800 | - | A |
| Presto | Nintendo DS browser | Nintendo DS | 8.5 | C/A^1^ |
| KHTML | Konqureror 3.1 | KDE 3.1 | 3.1 | C |
| KHTML | Konqureror 3.3 | KDE 3.3 | 3.3 | A |
| KHTML | Konqureror 3.5 | KDE 3.5 | 3.5 | A |
| Tasman | Internet Explorer 4.5 | Mac OS 8-9 | - | X |
| Tasman | Internet Explorer 5.1 | Mac OS 7.6-9 | 1 | C |
| Tasman | Internet Explorer 5.2 | Mac OS 8-X | 1 | C |
| Misc | NetFront 3.1 | Embedded devices | - | C |
| Misc | NetFront 3.4 | Embedded devices | - | A |
| Misc | Dillo 0.8 | Embedded devices | - | X |
| Misc | Links | Text only | - | X |
| Misc | Lynx | Text only | - | X |
| Misc | IE Mobile | Windows Mobile 6 | - | C |
| Misc | PSP browser | PSP | - | C |
| Other browsers | All others | - | - | U |

# Initialisation code

```
$(document).ready( function () {
	$('#example').dataTable( {
		"sDom": 'T<"clear">lfrtip<"clear spacer">T'
	} );
} );
```

# Other examples

- Basic initialisation
- Setting the SWF path
- Custom button arrangement
- Button text alteration
- Using button collections
- Using jQuery UI themes
- Styling with Twitter Bootstrap
- Multiple TableTools toolbars for a single table
- Multiple DataTables with a single initialisation
- User selectable rows (multiple rows)
- User selectable rows (single row)
- Alternative initialisation using 'new TableTools()'
- Altering the TableTools defaults
- TableTools plug-in buttons
- Customising the PDF output (adding text and orientation)

TableTools and DataTables © Allan Jardine 2009-2011.  
Flash 10 save / copy operations are performed by a modified version of Zero Clipboard.  
The icons used in the toolbar are modified from Oliver Twardowski's Flavours Icon set.
