# Supplementary material for: Molecule database framework: a framework for creating database applications with chemical structure search capability
Source: J Cheminform. 2013 Dec 11;5:48. doi: 10.1186/1758-2946-5-48 (PMC3892073; doi:10.1186/1758-2946-5-48)
Supplement: Additional file 4 — MDF simple web application source code of the mercurial changeset 16f39f4e447b. [file 1758-2946-5-48-S4.zip › src/main/webapp/resources/js/datatables/TableTools/tabs.html]

TableTools example


TableTools example

# Preamble

This example shows the basic initialisation of TableTools by simply including the 'T' marker in DataTables' sDom parameter. This tell DataTables to insert the TableTools toolbar in that location. Remember to include the Javascript and CSS source files as well!

It is worth noting that you might need to set the *sSwfPath* parameter to tell TableTools where to find the SWF file for copy and file save.

# Live example

- Trident browsers
- Gecko browsers

| Rendering engine | Browser | Platform(s) | Engine version | Grade |
| --- | --- | --- | --- | --- |
| Rendering engine | Browser | Platform(s) | Engine version | Grade |
| --- | --- | --- | --- | --- |
| Trident | Internet Explorer 4.0 | Win 95+ | 4 | X |
| Trident | Internet Explorer 5.0 | Win 95+ | 5 | C |
| Trident | Internet Explorer 5.5 | Win 95+ | 5.5 | A |
| Trident | Internet Explorer 6 | Win 98+ | 6 | A |
| Trident | Internet Explorer 7 | Win XP SP2+ | 7 | A |
| Trident | AOL browser (AOL desktop) | Win XP | 6 | A |

| Rendering engine | Browser | Platform(s) | Engine version | Grade |
| --- | --- | --- | --- | --- |
| Rendering engine | Browser | Platform(s) | Engine version | Grade |
| --- | --- | --- | --- | --- |
| Gecko | Firefox 1.0 | Win 98+ / OSX.2+ | 1.7 | A |
| Gecko | Firefox 1.5 | Win 98+ / OSX.2+ | 1.8 | A |
| Gecko | Firefox 2.0 | Win 98+ / OSX.2+ | 1.8 | A |
| Gecko | Firefox 3.0 | Win 2k+ / OSX.3+ | 1.9 | A |
| Gecko | Camino 1.0 | OSX.2+ | 1.8 | A |
| Gecko | Camino 1.5 | OSX.3+ | 1.8 | A |
| Gecko | Netscape 7.2 | Win 95+ / Mac OS 8.6-9.2 | 1.7 | A |
| Gecko | Netscape Browser 8 | Win 98SE+ | 1.7 | A |
| Gecko | Netscape Navigator 9 | Win 98+ / OSX.2+ | 1.8 | A |
| Gecko | Mozilla 1.0 | Win 95+ / OSX.1+ | 1 | A |
| Gecko | Mozilla 1.1 | Win 95+ / OSX.1+ | 1.1 | A |
| Gecko | Mozilla 1.2 | Win 95+ / OSX.1+ | 1.2 | A |
| Gecko | Mozilla 1.3 | Win 95+ / OSX.1+ | 1.3 | A |
| Gecko | Mozilla 1.4 | Win 95+ / OSX.1+ | 1.4 | A |
| Gecko | Mozilla 1.5 | Win 95+ / OSX.1+ | 1.5 | A |
| Gecko | Mozilla 1.6 | Win 95+ / OSX.1+ | 1.6 | A |
| Gecko | Mozilla 1.7 | Win 98+ / OSX.1+ | 1.7 | A |
| Gecko | Mozilla 1.8 | Win 98+ / OSX.1+ | 1.8 | A |
| Gecko | Seamonkey 1.1 | Win 98+ / OSX.2+ | 1.8 | A |
| Gecko | Epiphany 2.20 | Gnome | 1.8 | A |

# Initialisation code

```
$(document).ready( function () {
	$('#example').dataTable( {
		"sDom": 'T<"clear">lfrtip'
	} );
} );
```

# Other examples

- Basic initialisation
- Setting the SWF path
- Custom button arrangement
- Button text alteration
- Using button collections
- Using jQuery UI themes
- Styling with Twitter Bootstrap
- Multiple TableTools toolbars for a single table
- Multiple DataTables with a single initialisation
- User selectable rows (multiple rows)
- User selectable rows (single row)
- Alternative initialisation using 'new TableTools()'
- Altering the TableTools defaults
- TableTools plug-in buttons
- Customising the PDF output (adding text and orientation)

TableTools and DataTables © Allan Jardine 2009-2011.  
Flash 10 save / copy operations are performed by a modified version of Zero Clipboard.  
The icons used in the toolbar are modified from Oliver Twardowski's Flavours Icon set.
